# Developing diagnostic SNP panels for the identification of true fruit flies (Diptera: Tephritidae) within the limits of COI-based species delimitation

**DOI:** 10.1186/1471-2148-13-106

**Published:** 2013-05-29

**Authors:** Juerg E Frey, Larissa Guillén, Beatrice Frey, Joerg Samietz, Juan Rull, Martín Aluja

**Affiliations:** 1 Federal Department of Economic Affairs FDEA, Agroscope Changins-Wädenswil Research Station ACW, Department of Plant Protection, Schloss, P.O. 185, Wädenswil, 8820, Switzerland; 2 Instituto de Ecología, A.C, Apartado Postal 63, Xalapa, Veracruz, 91000, México

**Keywords:** Diagnostic SNPs, Single nucleotide polymorphisms, Species identification, Species delimitation, Quarantine pest insects, Tephritidae, Cytochrome oxidase I, COI

## Abstract

**Background:**

Rapid and reliable identification of quarantine pests is essential for plant inspection services to prevent introduction of invasive species. For insects, this may be a serious problem when dealing with morphologically similar cryptic species complexes and early developmental stages that lack distinctive characters useful for taxonomic identification. DNA based barcoding could solve many of these problems. The standard barcode fragment, an approx. 650 base pairs long sequence of the 5′end of the mitochondrial cytochrome oxidase I (COI), enables differentiation of a very wide range of arthropods. However, problems remain in some taxa, such as Tephritidae, where recent genetic differentiation among some of the described species hinders accurate molecular discrimination.

**Results:**

In order to explore the full species discrimination potential of COI, we sequenced the barcoding region of the COI gene of a range of economically important Tephritid species and complemented these data with all GenBank and BOLD entries for the systematic group available as of January 2012. We explored the limits of species delimitation of this barcode fragment among 193 putative Tephritid species and established operational taxonomic units (OTUs), between which discrimination is reliably possible. Furthermore, to enable future development of rapid diagnostic assays based on this sequence information, we characterized all single nucleotide polymorphisms (SNPs) and established “near-minimal” sets of SNPs that differentiate among all included OTUs with at least three and four SNPs, respectively.

**Conclusions:**

We found that although several species cannot be differentiated based on the genetic diversity observed in COI and hence form composite OTUs, 85% of all OTUs correspond to described species. Because our SNP panels are developed based on all currently available sequence information and rely on a minimal pairwise difference of three SNPs, they are highly reliable and hence represent an important resource for developing taxon-specific diagnostic assays. For selected cases, possible explanations that may cause composite OTUs are discussed.

## Background

Invasive species have been recognised as one of the most important threats to global biodiversity [1]. As a consequence, biosecurity, including the protection against the spread of pest species, is emerging as one of the most important issues facing the international community [2]. DNA barcoding has been developed as a powerful identification tool to contribute in the prevention of introduction, establishment and spread of invasive species [3]. However, it has been difficult so far to exploit this molecular tool to differentiate among species within the Diptera, particularly the economically important family Tephritidae [4]. Many species within Tephritidae cause severe economic damage and trigger trade restrictions [5], and as a result of expanded worldwide trade, are recurrently being detected across all continents [6-9].

Taxonomically, many quarantine species of this fly family are well described based on morphological characteristics of adults [10]. Nonetheless, the four genera of greatest economic importance (*Anastrepha*, *Bactrocera*, *Ceratitis* and *Rhagoletis*) include morphologically similar sibling species, complexes of cryptic species, host races, and morphotypes [11-17]. Distinguishing between species in cryptic species complexes is relevant because these include taxa of economic importance (e.g., *Bactrocera invadens*, *B*. *dorsalis*, *B*. *tryoni*), that could be confused with morphologically very similar non-pest species [12]. Additionally, different host affiliations of some morphotypes, such as within *Anastrepha fraterculus*, have important trade implications [18]. Furthermore, identification of larval stages has proven to be challenging due to the lack of distinctive, species specific morphological characters [19]. Since quarantine interceptions mostly contain larvae concealed within fruit, it is also important to develop tools for accurate identification of immature life stages of fruit flies.

Moreover, developing and fine tuning of molecular tools that can separate closely related sibling and incipient species is important for the study of evolutionary processes such as speciation. This is particularly interesting in the case of Tephritids because the family includes many species groups undergoing rapid radiation [12] some of which have been fostered as textbook examples of sympatric speciation [20]. More recently, phylogeographic studies have revealed the role of secondary contact and differential introgression in sympatric host race formation and speciation [21] in addition to cases of interspecies hybridization [22].

Within the frame of the Barcoding of Life Initiative (http://www.barcodeoflife.org), DNA sequence information of a standard fragment of the mitochondrial cytochrome oxidase I gene (COI) has been collected from a wide range of Tephritid fruit flies [10,23]. This molecular tool has been proven to be useful in identifying some species by analyzing a single larva, an egg, and even body fragments [24-26]. However, preliminary data suggested that the genetic diversity of the barcode fragment of COI is not large enough to enable unambiguous identification of all members of this important fly family [27]. This may be attributed to its young evolutionary history and the rapid radiation of many pest complexes [12,28]. Alternatively, it may be possible that several described species in fact represent a single interbreeding one.

The main aim of the present study was to use all sequence information currently available to define operational taxonomic units (OTUs) that reflect the maximum possible level of species discrimination provided by the COI gene, and furthermore, to describe near-minimal sets of single nucleotide polymorphisms (SNPs) that enable discrimination between all pairwise comparisons of these OTUs with at least three or four SNPs difference.

To accomplish this we sequenced ca. 1400 bp representing ca. 90% of the entire COI gene of 16 important Tephritid species/groups plus one undescribed species and we complemented this data set with all GenBank entries of Tephritid COI sequences available on January 2012. We used phylogenetic and character based methods to establish OTUs based on the 5′-barcoding region of COI and we compared these OTUs to the described species/species groups. The final goal was to provide diagnostic SNP panels for taxon identification that are composed of the minimal number of SNPs required to discriminate between all pairwise OTU comparisons with at least three to four SNPs. We used a custom script to first select candidate SNPs for each OTU and then screened the data space by testing one million randomly composed SNP sets of increasing size until at least one was found that fulfilled the conditions. The resulting diagnostic SNP panels may be exploited in the future for the development of rapid SNP-based diagnostic tests.

## Results

### In-house sequencing

We sequenced a total of 151 specimens (2–19 samples per species) representing 18 putative species (Table 1 GenBank accession numbers HQ677034–HQ677184, following the listing in this table). We sequenced an overall average of 1430 bp (range 626–1542 bp) of the COI gene with at least 1421 bp for over 92% of all individuals and a minimum consensus sequence of 1315 bp. Note that the Mexican morphotype of *Anastrepha fraterculus* is in all likelihood a different species than its South American relatives [15,18]. Established sequence data depended on the sequence match of the primers and, as these were not the same for all species due to target site variation, the readable sequences were found to be variable between species and individuals.

**Table 1:** Origin of samples sequenced in this study

**Species**	**Population (location**, **region**, **country)**	Host*	**N sequences**
*Anastrepha fraterculus* (*Afra*)	Teocelo, Veracruz, MEX	*Prunus persica*	2
	Xico, Veracruz, MEX	*Psidium guajava*	3
*Anastrepha ludens* (*Alud*)	Troncones, Nuevo León, MEX	*Citrus aurantium*	1
	Hualahuises, Nuevo León, MEX	*Casimiroa edulis*	2
	Hualahuises, Nuevo León, MEX	*Casimiroa greggi*	2
	Quimixtlán, Puebla, MEX	*Casimiroa edulis*	2
	Cuautla, Morelos, MEX	*Citrus paradisi*	2
	Pathe, Hidalgo, MEX	*Casimiroa edulis*	2
	Xalapa, Veracruz, MEX	*Casimiroa edulis*	2
	Miradores, Veracruz, MEX	*Citrus aurantium*	2
	Alborada, Veracruz, MEX	*Citrus paradisi*	1
	Tamaulipas, MEX	*Citrus paradisi*	2
	Teocelo, Veracruz, MEX	*Prunus persica*	1
*Anastrepha obliqua* (*Aobl*)	Jalcomulco, Veracruz, MEX	*Spondias mombin*	3
	Tapachula, Chiapas, MEX	*Mangifera indica*	3
*Anastrepha serpentina* (*Aser*)	Tapachula, Chiapas, MEX	*Chrisophillum caimito*	3
	Veracruz, Veracruz, MEX	*Manilkara sapota*	2
*Anastrepha striata* (*Astr*)	Actopan, Veracruz, MEX	*Psidium guajava*	3
	Cuautla, Morelos, MEX	*Psidium guajava*	3
	Tapachula, Chiapas, MEX	*Psidium guajava*	3
*Rhagoletis cingulata* (*Rcin*)	Cuapiaxtla, Tlaxcala, MEX	*Prunus serotina*	3
	Virginia, USA	*Prunus serotina*	3
	Los Lirios, Coahuila, MEX	*Prunus serotina*	3
	Allegan, USA	*Prunus serotina* (?)	2
	Traverse City, USA	*Prunus serotina* (?)	2
	Hart, USA	*Prunus serotina* (?)	4
*Rhagoletis completa* (*Rcom*)	San José Boquillas, Nuevo León, MEX	*Juglans spp*	3
	Valle de los Fantasmas, San Luis Potosí, MEX	*Juglans spp*	3
	Basel, Basel, CHE	*Juglans regia*	3
	Bern, Bern, CHE	*Juglans regia*	2
	Faido, Tessin, CHE	*Juglans regia*	1
	Château Neuf, Wallis, CHE	*Juglans regia*	2
	Jacala, Hidalgo, MEX	*Juglans spp*.	3
	Dombresson, Neuenburg, CHE	*Juglans regia*	1
	Rorschach, Sankt Gallen, CHE	*Juglans regia*	1
*Rhagoletis pomonella* (*Rpom*)	Cerro el Potosí, Nuevo León, MEX	*Crategus rosei rosei*	3
	El Sedeño, Veracruz, MEX	*Crataegus rosei parrayana*	3
*Rhagoletis solanophaga* (*Rsol*)	Xalapa, Veracruz, MEX	*Solanum spp*	3
*Rhagoletis turpiniae* (*Rtur*)	Xalapa, Veracruz, MEX	*Turpinia insignis*	2
*Rhagoletis zoqui* (*Rzoq*)	Acajete, Veracruz, MEX	*Juglans regia*	6
	El Madroño, Queretaro, MEX	*Juglans sp*.	3
	Cuapiaxtla, Tlaxcala, MEX	*Juglans regia*	3
	Tlacolulan, MEX	*Juglans sp*.	2
*Rhagoletis ramosae* (*Rram*)	Miichoacán, MEX	*Juglans sp*.	3
*Toxotrypana curvicauda* (*Tcur*)	Teocelo, Veracruz, MEX	*Carica papaya*	3
	Tamaulipas, MEX	*Carica papaya*	3
*Rhagoletis suavis* (*Rsua*)	West Virgina, EUA	*unknown*	3
*Anastrepha sp nov* (*Aspec*)	Tehuacan, Puebla, MEX	*Euphorbiacea tehuacana*	3
*Bactrocera oleae* (*Bole*)	Thessaloniki, GRC	*unknown*	8
*Rhagoletis cerasi* (*Rcer*)	Waedenswil, Zuerich, CHE	*Prunus avium*	5
	Dosenheim, DEU	*Prunus avium*	6
*Ceratitis capitata* (*Ccap*)	La Antigua, GTM	*Coffea arabica*	5
	Thessaloniki, GRC	*Unknown*	6

*) unknown for undetermined host species; (?) where species status not certain.

Indicated is the species, sampling location, region and country, host plant and the number of samples.

The data established in our laboratory were complemented with entries from GenBank and/or the BOLD database and the combined set of accessions was finally composed of 193 described species, with 1–47 individuals per species (see Additional file 1: Table S1).

### Cluster analysis

We used neighbour-joining (NJ), maximum likelihood (ML) and Bayesian (BA) methods to perform a phylogenetic clustering of the sequences. For the purpose of this study we were only interested in the terminal grouping and not so much in the phylogenetic histories of the putative species. With few exceptions, all three methods resulted in the same terminal groups, and these had in general above 90% bootstrap support (NJ, ML) and a posterior probability above 0.9 (BA) (see Additional file 2: Figure S1). In total, the number of described species studied here summed up to 192 plus AnaSP.

Although bootstrap values at deep levels are low, there is a trend to form “genus”-clusters, as for all genera, clusters containing multiple described species are observed (Additional file 2: Figure S1). In total, 92 clusters with high bootstrap support, (i.e., generally >90% ML and NJ, and > .9 posterior probability for BA) were obtained. Among these were 14 mixed clusters containing more than a single described species which results in 84.8% OTUs that match described species of Tephritidae (Additional file 2: Figure S1).

### Taxon delimitation

**Figure 1:**
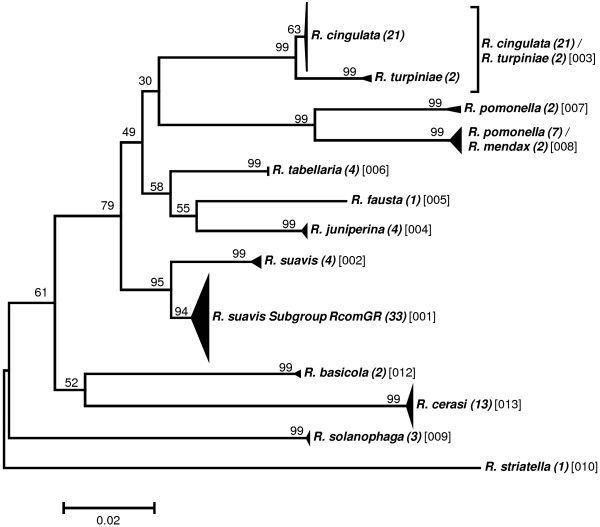
**Evolutionary relationships among *Rhagoletis *species.**

**Figure 2:**
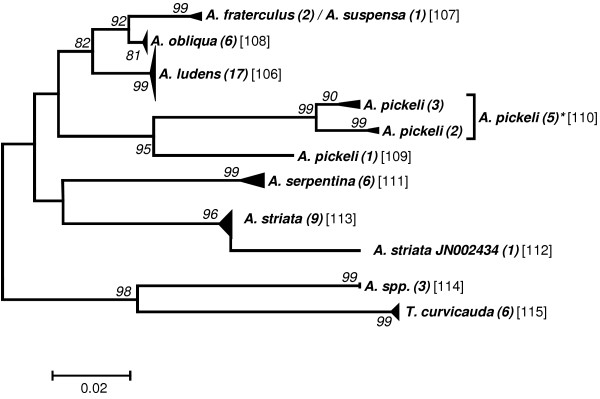
**Evolutionary relationships among *Anastrepha *species (including *Toxotrypana curvicauda*).**

**Figure 3:**
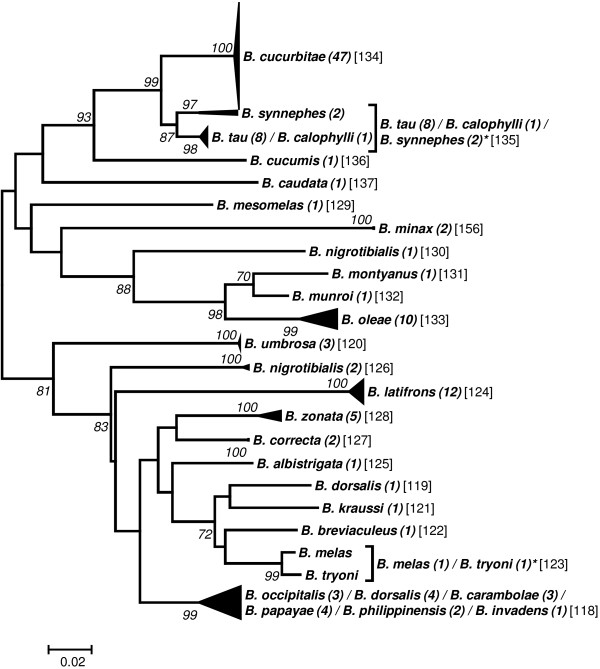
**Evolutionary relationships among *Bactrocera *species.**

**Figure 4:**
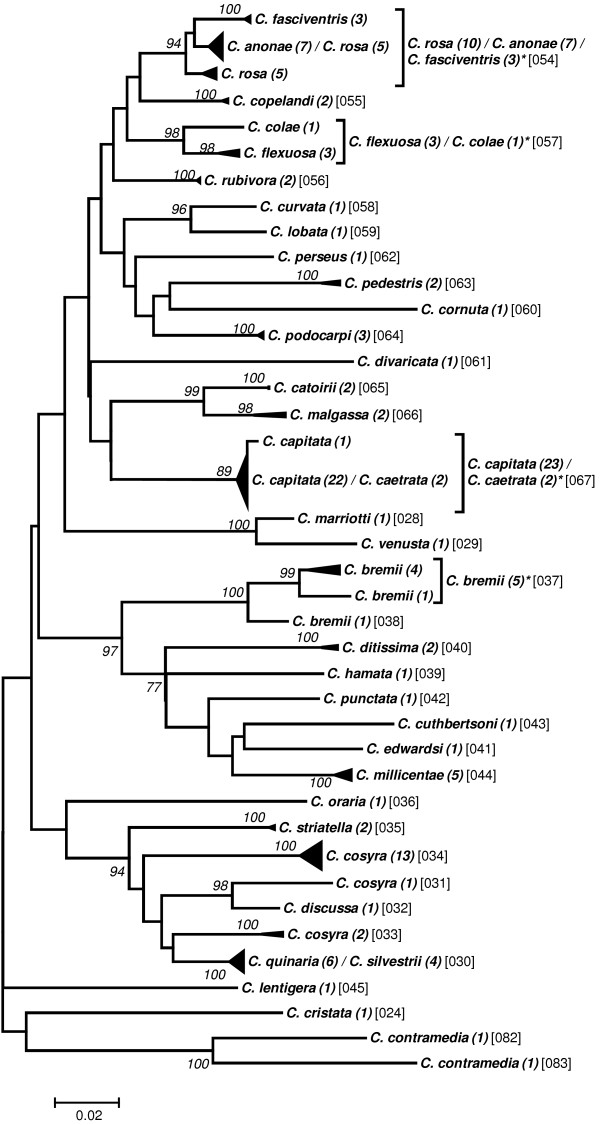
**Evolutionary relationships among *Ceratitis *species.**

**Figure 5:**
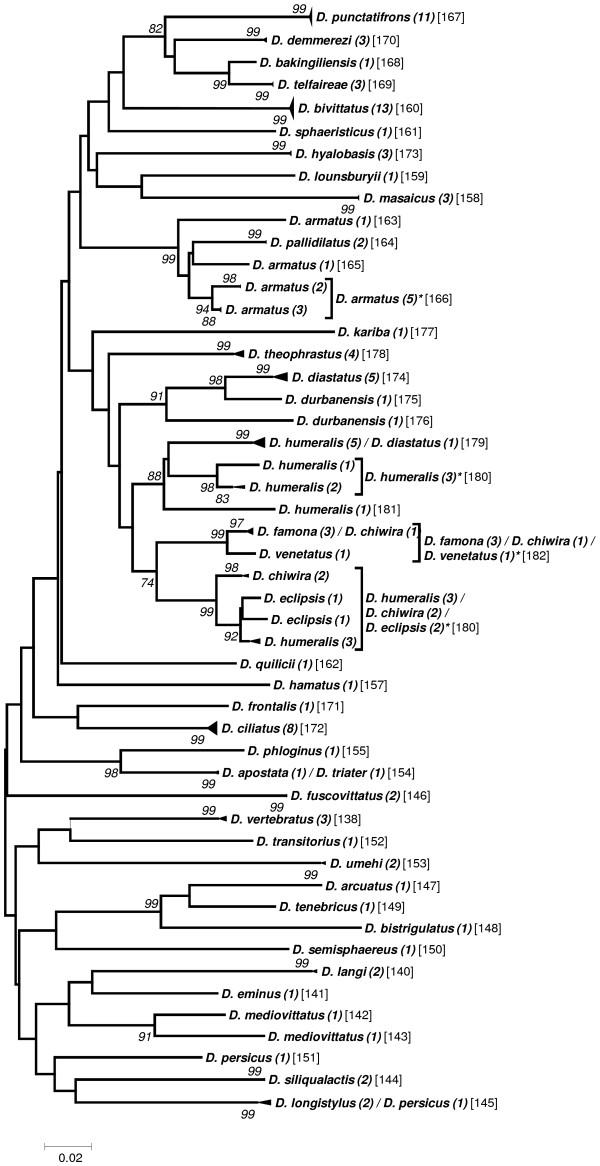
**Evolutionary relationships among *Dacus *species.**

**Figure 6:**
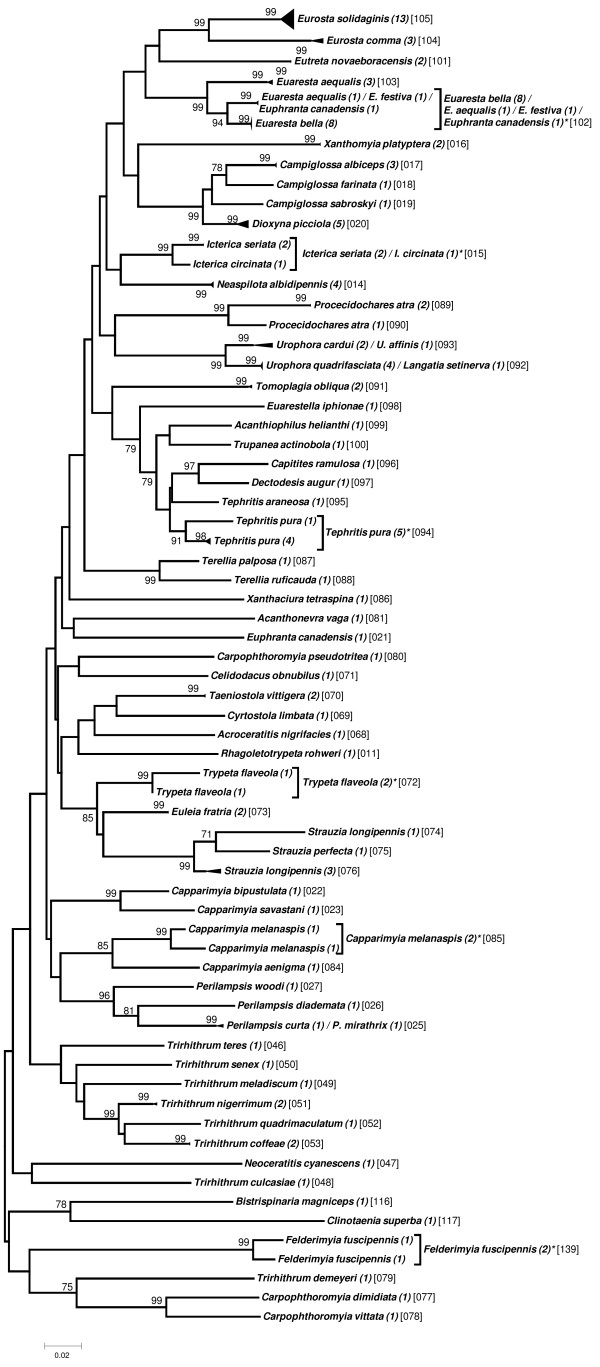
Evolutionary relationships among all other species.

Preliminary analyses to elaborate a taxon delimitation threshold showed that a single threshold for the entire data set would produce less than optimal results by splitting some seemingly homogeneous clusters while pooling other, obviously well separated clusters (data not shown). As the phylogenetic clustering showed a trend to form “genus”-groups we performed the taxon delimitation analyses using the five genera *Anastrepha* (including *T*. *curvicauda*), *Bactrocera*, *Ceratitis*, *Dacus*, and *Rhagoletis*. The remaining samples were pooled as the group “Others”. Our data set contains many singletons, i.e., described species represented by only a single sample (see Additional file 1: Table S1). Because this may affect the calculations of taxon delimitation thresholds in SPIDER [29], we performed the analyses both with and without taking these singletons into account. Our results confirm that singletons may have a considerable effect, decreasing the species delimitation threshold in some cases to half that obtained when singletons were excluded (e.g., from 2.53 to 1.20 in *Dacus*; see Table 2). Furthermore, considerable variation in the threshold level between the genera exists, ranging from a low of 1.81 (“Excluding Singletons”) in *Anastrepha* to 3.6 in *Ceratitis* (Table 2). The taxon delimitation thresholds establish OTUs that are in very good agreement with the clusters formed by the phylogenetic tree construction methods (Figures 1, 2, 3, 4, 5 and 6).

**Table 2:** Taxon delimitation thresholds including and excluding singletons for the six taxon groups

	**Including singletons**		**Excluding singletons**	
**Taxon**	**Threshold**	**N OTUs**	**Threshold**	**N OTUs**
*Anastrepha* *	1.70	11	1.81	10
*Bactrocera*	3.27	22	3.41	21
*Ceratitis*	1.90	39	3.60	35
*Dacus*	1.20	50	2.53	43
*Rhagoletis*	1.70**	12	1.97	12
Others	2.25	67	3.33	61
ALL	mix low	202	mix high	182

* (incl. *Toxotrypana curvicauda*).

**) using the empirically established threshold of 1.50 differentiates between *R*. *cingulata* and *R*. *turpiniae*, increasing the N OTUs of *Rhagoletis* ‘Including Singletons’ to 13.

“N OTUs” indicates the number of OTUs obtained using the corresponding threshold. “mix low”, “mix high”: the corresponding thresholds for each group were taken to establish the number of OTUs for the combined data set (All).

### Determining near-minimal diagnostic SNP panels

Once the OTUs are elaborated, the search for SNPs suitable to be included in diagnostic SNP panels can be started. The problem to be addressed searching for minimal sets of SNPs with an optimal pairwise differentiation among OTUs is often called “feature selection” (e.g., [30]) and is used to address similar questions, e.g., to find tag SNPs for capturing haplotype patterns used in studies on disease association and drug design (e.g., [31,32]; see [33] and references therein), or for computer-aided identification of polymorphism sets for bacteria and viruses [34]). However, none of these were designed for our exact purpose. To accommodate our workflow and our SNP candidate selection preference scheme we developed our own script. It selects the best 25 SNPs per OTU according to our preference scheme and then screens increasingly large sets of random combinations of these candidate SNPs to establish “near-minimal” diagnostic panels. We refer to the SNP panels found with our script as “near-minimal” because, out of the excessive number of possible combinations, we only screen one million sets for each set size. This number may be increased at the cost of longer runtimes.

Once diagnostic SNPs are found it may prove to be difficult to translate this information into a reliable SNP-based assay. Generally, about 25–50% of candidate SNPs will not perform as expected. We therefore set out to find sets of SNPs with a minimal differentiation between each pairwise comparison of three and four SNPs. At a translation success rate (DNA sequence data into SNP assay) of 50% this would leave a minimum of one to two SNPs difference between each pairwise comparison. We performed our script-based search for sets of diagnostic SNPs screening one million random combinations of SNP candidates that were selected, giving preference to non-silent SNPs and to those with higher degeneracy. The script is given an initial number of SNPs per set, e.g. ten SNPs, and if it does not find a set of ten SNPs that differentiate among all pairwise comparisons with at least three or four SNPs, respectively, it increases this number of SNPs per set by one to give, in our example, 11 SNPs for the next set, and starts the search again. The number of possibilities to screen the complete data set is extremely large (in excess of 10^28^) and hence, screening one million random combinations does not guarantee to find the minimal set. However, we are confident that our script finds “near-minimal” sets of SNP combinations. We used both thresholds, i.e., the singleton inclusive and exclusive ones, to do the SNP searches. For a minimal pairwise difference of four base pairs between the groups using the singleton inclusive and exclusive threshold we found in average 31.4 ± 13.4 and 23.8 ± 8.6 SNPs (range 16–46), respectively, and for three base pairs the corresponding set sizes were 22.2 ± 10.5 and 17.2 ± 6.4 (range 11–34; Table 3).

**Table 3:** **Number of SNPs per group to differentiate between each pair of OTUs within that group with at least three or four SNPs difference**, **respectively**, **based on the thresholds established including and excluding singletons**

**N SNPs**	**4**		**3**	
**Singletons:**	**including**	**excluding**	**including**	**excluding**
Anastrepha*	20	16	11	11
*Bactrocera*	20	18	14	13
*Ceratitis*	43	33	30	24
*Dacus*	46	25	31	17
*Rhagoletis*	19	16	13	12
Others	43	35	34	26
ALL	113	74	81	52

* (incl. *Toxotrypana curvicauda*).

## Discussion

Genetic variation is ubiquitous in nature and in general, the frequency distribution of variants is such that few are frequent and quite many are very rare (e.g., [35]). This means that to collect the full genetic variation of any taxon requires very large sample sizes (e.g., [36]). Yet it also means that much of the variation characterizing a taxon may be contained in a sample of reasonable size [37]. This may be among the main causes (together with the fast lineage-sorting characteristics of mitochondrial DNA) for the overall good success of COI based barcoding [38].

The broad aim of this study was to design, based on the barcoding fragment located in the 5′-region of the mitochondrial COI gene, diagnostic SNP panels with a pre-determined level of differentiation between all pairwise taxa for as many Tephritid species as possible. To enable this requires, as an initial step, to define operational taxonomic units (OTUs). We established OTUs through a phylogenetic clustering of the COI fragment followed by a taxon delimitation step using density depressions in genetic distances calculated on K2P distance matrices [29]. We find that a priori ca. 85% of all OTUs correspond with the taxonomic species level. Taking into account several cases which our results suggest to be misidentifications, this number even exceeds 90%. The remaining 10% are mostly composed of mixed groups containing closely related sister and cryptic species. Hence, the failure of COI to differentiate among such sister species may simply reflect a very recent ancestry, in specific cases below the species level, that did not yet result in complete lineage sorting (e.g., [39]).

Below we examine some of the grouping peculiarities of each genus and of the combined group “Others”:

### Rhagoletis

**Figure 7:**
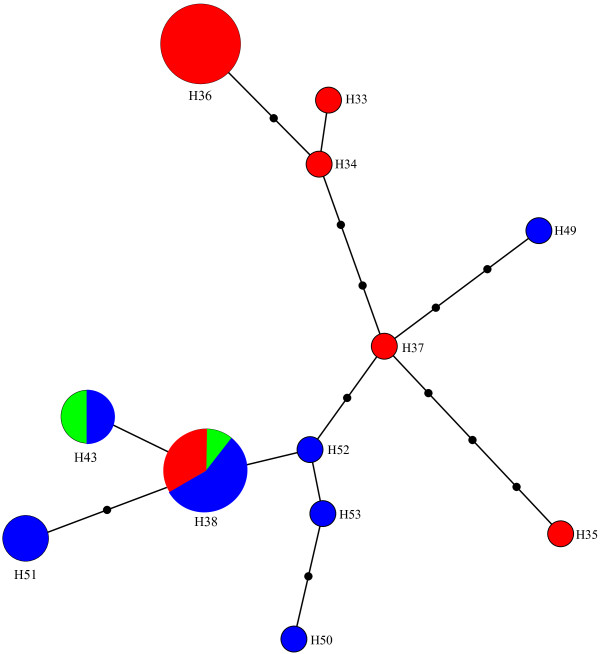
**Haplotype minimum-spanning network for the *Rhagoletis suavis *subgroup RcomGR. Circle sizes represent the number of haplotypes per haplogroup (indicated by H, followed by a number).** Red = *R*. *completa* (N = 16); blue = *R*. *zoqui* (N = 14); green = *R*. *ramosae* (N = 2). Haplotype H38 is shared among all three described species.

Both taxon delimitation thresholds, i.e., the high threshold established “Excluding Singletons” and the low threshold “Including Singletons”, produced the same OTUs (Figure 1). Twelve OTUs were obtained for the 16 described species included, of which nine represent single described species clusters and three are composite OTUs. Unlike the tree based approach, the taxon delimitation algorithm is unable to differentiate between *R*. *cingulata* and *R*. *turpiniae* using the threshold established based on the data. However, the empirically determined threshold of 1.5 would differentiate among these two species without having an effect on the other OTUs (Figure 1). The *R*. *pomonella* group is split into two genetically different units, one homogeneous group with four *R*. *pomonella* and a second composite OTU with seven *R*. *pomonella* and two *R*. *mendax*. These two species are very closely related sibling species [11,28,40,41] and hence it is not surprising that they cannot be differentiated by COI alone. However, the occurrence of two clearly distinct *R*. *pomonella* groups is noteworthy and deserves further investigation. The *R*. *suavis* subgroup RcomGR is the third composite OTU of this genus and is composed of the three species *R*. *completa*, *R*. *ramosae* and *R*. *zoqui*. All three are well-described species that share a common host, walnut (*Juglans* sp.) [42-44]. Furthermore, they have geographically partly overlapping distribution ranges and have been shown to hybridize in nature [22]. Provided the voltinism periods are at least partly overlapping, these are conditions that may favour mitochondrial introgression, i.e., the introduction of a mitochondrial genotype from another species by “accidental” pairing of two different species, a mechanism not uncommon in insects (e.g., [45-47]) and which should result in shared haplotypes. We therefore performed a haplotype network analysis to assess if the different taxa share identical haplotypes. With the exception of the *R*. *suavis* subgroup RcomGR, there were no shared haplotypes among taxa. The three taxa combined as the RcomGR subgroup, i.e., *R*. *ramosae*, *R*. *zoqui*, and *R*. *completa*, showed the only case of a shared haplotype (Figure 7). These results lend support to the mitochondria introgression hypothesis among the three species *R*. *completa*, *R*. *ramosae* and *R*. *zoqui*. It is clear that mitochondrial introgression precludes differentiation as the marker gene is identical among the taxa concerned and has recently been confirmed through finding of natural hybrids in a contact zone in northeastern Mexico [22]. In the genus *Rhagoletis*, the *suavis* group is the only one where all species have different wing patterns and body coloration, and three of them are sexually dimorphic for body and wing markings [11]. This evidence has suggested that these species have undergone several periods of sympatry and isolation where flies developed the color patterns to avoid maladaptive hybridization in sympatry [41].

Additionally, several examples of sympatric speciation through host plant shifts have been identified in the flies in the genus *Rhagoletis*[20], some of which occurred within the past 150 years [11]. Interestingly, results from our study may have highlighted a yet undiscovered similar event. *Rhagoletis turpinia* and *Rhagoletis cingulata* are two currently parapatric species that may have been in contact during glaciations when the pine-oak forest expanded its range in mountainous areas of Mexico [48]. The remarkable genetic similarity between these species revealed in our study could be due to a shift in host odour recognition producing immediate and strong prezygotic isolation among individuals exploiting different plants [20]. If so this could be confirmed by further studies exploring reproductive compatibility between these species. A similar explanation applies to *R*. *mendax* and *R*. *pomonella* in our study and again attests to the complexity of taxonomic and genetic characterization of Tephritidae.

### Anastrepha

Eleven OTUs are observed in this group of nine described species, of which one is a composite and two are split OTUs (Figure 2). The genetic differentiation in the barcoding fragment of COI seems to preclude separation of the two described species *A*. *fraterculus* and *A*. *suspensa*. None of the grouping methods was able to differentiate among these two species. An investigation of their sequences shows that six SNPs seem to exist, corresponding to just short of 1% of genetic differentiation. However, as only two *A*. *fraterculus* sequenced in-house and a single sequence of *A*. *suspensa* from GenBank containing a number of ambiguous base calls are contained in this data set, further data are clearly needed to clarify the situation in this OTU. The *fraterculus* species group of *Anastrepha* is composed of at least 29 species which include several sister and cryptic species that exploit different hosts in sympatry [16,49,50] and among which genetic exchange is still possible (e.g., [51]). These patterns may be a reflection of recent and rapid differentiation in the group and may explain the difficulties we had in separating *A*. *suspensa* from *A*. *fraterculus* in this study. In general, several species groups of Tephritids are also undergoing rapid radiation, a fact that renders taxonomic and genetic characterization particularly complicated. More analyses including *A*. *fraterculus* data from South America should be performed to separate Mexican and South American *A*. *fraterculus* from *A*. *suspensa*.

Another case of very limited genetic differentiation was observed in the species *Anastrepha ludens*. It is interesting to note that despite having collected six different hosts in markedly different environments (ranging from 400 to 1500 mm of annual precipitation) all of the *A*. *ludens* specimens examined here appeared to be genetically homogenous. This attests to the high phenotypic plasticity and polyphagous nature of the species which allows its members to exploit a wide range of environments without undergoing differentiation [52].

A contrasting picture is observed in the species *Anastrepha pickely*. This species seems to be genetically highly diverse with only 89.9% identical sites and a pairwise identity of 96.2%. As a consequence, three and two OTUs are formed with the “Including Singletons” and the “Excluding Singletons” threshold, respectively. This degree of differentiation may point to the existence of cryptic taxa, as was shown to occur frequently in insects (e.g., [53-55]). The species group *A*. *striata* is homogeneous but shows one outlier that clusters sister to this group but in a separate OTU. This outlier contributes most to the differentiation within this group that amounts to 3.1% different sites and 99.3% pairwise identity.

### Bactrocera

The two taxon delimiting thresholds established for *Bactrocera* produce essentially the same OTUs. The 27 described *Bactrocera* species included in our study clustered into 23 OTUs for the “Including Singletons” and 21 OTUs for the “Excluding Singletons” threshold (Figure 3). Only two composite groups are obtained using the lower “Including Singletons” threshold. One combines the two described species *B*. *tau* (8 sequences) and *B*. *calophylli* (one sequence). This grouping is highly disputable, because these two species are taxonomically quite different belonging to two different subgenera. *B*. *calophylli* belongs to the subgenus *Gymnodacus* which, using different genetic markers such as COI, COII and 16SrRNA, forms clusters far apart from those of *B*. *tau* that is a member of the subgenus *Zeugodacus*[56,57]. As the closely clustering *B*. *cucurbitae* also belongs to the subgenus *Zeugodacus*, *B*. *tau* seems to be correctly assigned. This suggests that the individual *B*. *calophylli* specimen is a false assignment and hence that this is also a homogeneous species group.

The second composite group is the *B*. *dorsalis* species complex containing, in our data set, six species that are assigned to this complex, i.e., *B*. *dorsalis*, *B*. *occipitalis*, *B*. *carambolae*, *B*. *papaya*, *B*. *invadens* and B. *philippinensis* (e.g., [12,58]. There is ongoing dispute about how many of these species should be joined into one. Krosch *et al*. [59], using microsatellites, found no significant structuring between *B*.*dorsalis* and *B*. *papayae* populations across the biogeographic barrier of the isthmus of Kra in Thailand, suggesting that these two species represent a panmictic population that was at origin the result of an incorrect taxonomical split. A second example in support of merging several putative species in the *B*. *dorsalis* complex into a single entity comes from Khamis *et al*. [60], who found, after morphometric analysis and barcoding, that *B*. *invadens*, *B*. *kandiensis*, and *B*. *dorsalis* essentially belong to a single cluster. Finally, using similar techniques, Schutze *et al*. [61] conclude that *B*. *dorsalis*, *B*. *papayae*, and *B*. *philippinensis* likely represent a single species. Importantly, our results also strongly support the notion that the *B*. *dorsalis* complex is more likely to be a specific entity than a complex of cryptic species. All the latter have important practical implications, as the designation of *B*. *invadens* has caused great harm to many least developed African countries who cannot export their fruit due to the purported presence of this “species”. Based on the unequivocal evidence accrued over the past five years (above cited references and our work), we propose that *B*. *invadens* should be synonymized with *B*. *dorsalis*.

Two OTUs are grouped together with the high “Excluding Singletons” threshold. One is the above discussed composite group *B*. *tau* / *B*. *calophylli* probably representing the species *B*. *tau* that is combined with the group *B*. *synnephes*. Little is known about the phylogenetic relationships of *B*. *synnephes* and hence this grouping is not discussed further. The second pair of OTUs that is combined by applying the threshold “Excluding Singletons” are the two species *B*. *melas* and *B*. *tryoni* which both belong to the *B*. *tryoni* species complex [62] (Figure 3).

One individual sequence of each of two species did not cluster together with the other members of this species with the same name, i.e., *B*. *nigrotibialis* and *B*. *dorsalis* (Figure 3). Four sequences of the latter species cluster, as expected, with the *B*. *dorsalis* complex whereas a single sequence groups loosely with *B*. *kraussi* which is not a member of this complex. Therefore, this individual *B*. *dorsalis* sequence may again represent a misidentification. Two of the three sequences of *B*. *nigrotibialis* form a small cluster associated to *B*. *latifrons* and *B*. *correcta*, and these two specimens were identified and provided to us by a taxonomist [63]. The third sequence forms an own OTU and seems to be another case of a misidentification.

### Ceratitis

The 36 described species included in this analysis form 40 OTUs with the low “Singleton Including” threshold, indicating the existence of split species clusters (Figure 4). Two different types of split groups are observed, one represented by *C*. *bremii*, an obviously quite divergent species; the other type is represented by *C*. *cosyra* which occurs in one large OTU with 13 individual sequences, a second one with two sequences associated with one of three composite groups, i.e., *C*. *quinaria* / *C*. *silvestrii*, and the third OTU is formed by a single sequence associated with *C*. *discussa*. The most likely candidate for the true species is OTU one with 13 sequences. Thus, the two other OTUs may be misidentifications.

In total, three composite clusters are observed in *Ceratitis* using the “Singleton Excluding” threshold: The one mentioned above composed of *C*. *quinaria* and *C*. *silvestrii*, the two described species *C*. *anonae* together with *C*. *rosa*, and *C*. *capitata* with *C*. *caetrata*. Whereas we have too little information to discuss the first group, evidence from the mitochondrial ND6 gene supports the close taxonomic relationships among *C*. *rosa* / *C*. *anonae* and *C*. *capitata* / *C*. *caetrata*[19]. Using the “Singleton Excluding” criterion, the composite group *C*. *rosa* / *C*. *anonae* is merged with the closely related *C*. *fasciventris*. These two taxa were described as the CAP-CAET and the FAS taxon, respectively, by Barr *et al*. [64], who showed, using different analytical methods, that discrimination based on COI alone is impossible.

Only two other new composite clusters are formed using the “Singleton Excluding” threshold. The two closely related *C*. *colae* and *C*. *flexuosa* are merged and the composite group *C*. *capitata* / *C*. *caetrata* now includes also the “outlier” specimen of *C*. *capitata*. Only one of the two “outlier” specimens of the diverse species *C*. *bremii* is included in the OTU with this high threshold. Finally and independent of the threshold used, the species *C*. *contramedia* splits in two OTUs which suggests that this described species may also be composed of different subgroups.

### Dacus

Our results suggest that, among the five genera assessed here, *Dacus* seems to be the most difficult with respect to species identification. The 41 described species cluster into 50 OTUs with the threshold “Including Singletons” (Figure 5). This again indicates the existence of split groups of which seven were found. Their composition is partly complex. The species *D*. *armatus* forms a cluster with four OTUs and includes a second species, *D*. *pallidilatus*, as a fifth OTU. The species *D*. *durbaniensis* forms to OTUs, one of which is closer to the species OTU *D*. *diastatus* than to the second *D*. *durbaniensis* OTU. The third split group is *D*. *diastatus* that forms the OTU mentioned above, composed of five individual specimens. A sixth specimen is included in one of five different OTUs of *D*. *humeralis*. As that OTU contains five *D*. *humeralis* and only this one “outlier” specimen of *D*. *diastatus* it may safely be assumed that this *D*. *diastatus* specimen is another case of misidentification. The fourth split group is the above mentioned *D*. *humeralis* which forms five OTUs, three of which contain five, three and two individual specimens, respectively, whereas the other two are composed of singletons. One of these singletons is merged with the *D*. *humeralis* OTU with two sequences when using the threshold “Excluding Singletons”. Even then, there are still three *D*. *humeralis* OTUs left that group together, suggesting it to be another of the highly diverse groups. The fifth *D*. *humeralis* OTU containing three specimens is associated with *D*. *eclipsis* in another subcluster of the phylogenetic tree and hence, if weight is placed on group size, this may be due to misidentification. The fifth case of split group is *D*. *chiwira* that forms two OTUs, one composed of two sequences and sister to the probably misidentified fifth *D*. *humeralis* OTU that is associated with *D*. *eclipsis*. The second OTU of *D*. *chiwira* is a single specimen clustering together with *D*. *famona* in a composite OTU with three *D*. *famona* specimens, a pattern that we think suggests misidentification. The sixth case of split OTU is *D*. *mediovittatus* that does not fall into a single OTU independent of the threshold applied. Finally, the last split group is *D*. *persicus*, forming a singleton OTU associated with *D*. *siliqualactis* and *D*. *longistylus*, whereas the second sequence of *D*. *persicus* in our data set clusters together with two sequences of *D*. *longistylus*. Although this pattern suggests that the sequence in the *D*. *longistylus* cluster is a misidentification, the issue cannot be settled with the current data set. In addition to the three composite OTUs discussed above, there is a fourth composite OTU formed by *D*. *apostata* and *D*. *triater*. As both contain only a single specimen more data have to be collected to assess this case.

### Others

This group is composed of 63 different species originating from 37 different genera (Figure 6). They are distributed into 67 OTUs with only two of them being composite.

Despite the challenging cases discussed here, the resolution provided by COI for taxon discrimination is generally considered useful (e.g., [64]), a view that we share, since overall, the success of COI for species delimitation is quite overwhelming (see for example the Barcode of Life Initiative, http://www. barcodeoflife.org).

The main problem in the process of determining diagnostic SNP panels is that the available data will never include the complete genetic diversity of the system. Hence, each new sample that is added to the data set may eliminate one or more of the SNPs of the diagnostic set established without that taxon (e.g., [65]). For example, if a new sample that is added to an existing SNP database shows a different nucleotide at any of the so far diagnostic SNP positions and if this new nucleotide is shared with another OTU, this SNP is lost as it is no longer diagnostic. Hence it would be very important to dispose of a large enough sample of each taxon, preferably collected over a large part of its distribution range, to establish robust OTUs and, based on this, diagnostic assays. Unfortunately, this will not be feasible for the foreseeable future. Therefore, the efforts to build OTUs that are close to the real world have to continue and it is highly likely that some of the “diagnostic” assays developed so far will have to be modified to mirror new knowledge. For example, Chua *et al*. [66] describe an allele-specific diagnostic PCR-assay for *Bactrocera carambolae* and *B*. *papayae* based on COI. They used ten individuals of *B*. *carambolae* from two different sample locations and nine individuals of *B*. *papayae* from three sample locations. They found three regions with putative species specific SNPs which they used for their design of allele-specific PCR primers. Their forward primer contained three and the two reverse primers four and two SNPs, respectively. Unfortunately, none of these nine SNPs could be confirmed with the samples we used in our study (see Additional file 1: Table S1). Hence, their allele-specific assay will most probably not be specific if used on populations other than those the authors used.

A similar problem can be observed in the panels we established with our script for the individual genera. For example, for the genus *Bactrocera*, our script finds “near-minimal” SNP panels of just 14 SNPs for a pairwise differentiation of three SNPs. However, the number of different SNPs of this panel decreases in pairwise comparisons beyond this genus. For example, using this SNP panel in a comparison between *B*. *alba* and *Ceratitis bremii*, the difference drops to just a single SNP, or to two between *B*. *cucumis* and *Dacus hamatus* (data not shown).

Three strategies may be used to compensate at least partly for this problem. The first is, as mentioned above, to increase the sample number of each taxon to a level at which most of the taxon-specific genetic diversity is contained in the data base. However, by increasing the number of species/OTUs that should be included in a diagnostic assay, the likelihood that this can be performed decreases-too many specimens would have to be included.

The second strategy is to increase data redundancy by increasing the number of SNPs used to differentiate between pairwise comparisons, for example from the three to four used in our analysis to maybe six or more. This will increase the number of SNPs of panels used to differentiate between the taxa (see below) yet it will generally lower the chance for false positive identification.

The third strategy is to use all available data of, e.g., an entire family and establish the “near-minimal” panel of SNPs required to differentiate between all pairwise comparisons with the desired number of SNPs. This will result in larger SNP panels but it will greatly reduce the chance for false positives. If required, the number of SNPs to be used may then be reduced by removing SNPs that are monomorphic within the taxon of interest from the panel. Following this strategy, we performed our script-based search with the entire data set of 202 OTUs with the threshold “Including Singletons” and we found several “near-minimal” SNP panels composed of 113 SNPs for the four and composed of 81 SNPs for the three base pair differentiation, respectively. The same search using the singleton excluding threshold is based on 182 OTUs and found panels of 74 SNPs for the four base pair differentiation and of 52 SNPs for the three base pair differentiation level.

Our script is quite effective in its search of the data space, as the number of SNPs found using the entire data set is much lower than if one would simply combine the SNP sets found for the genus groups mentioned above. For example, the sum of SNPs over all genus groups is 191 for the four base pair differentiation with the “Including Singletons” threshold. For this scenario, the search using all taxa found sets of only 113 SNPs which is a 40% reduction and hence a considerable improvement even in cases of partial overlap of the set compositions. As our script designs panels of random composition of candidate SNPs and screens only a small fraction of all possible combinations, we cannot expect that it finds the true minimal set of SNPs. However, as most of our repeat runs came up with identical SNP panels it should be safe to state that the script finds “near-minimal” panels.

One near-minimal SNP panel resulting from the analysis using the “Excluding Singletons” threshold (i.e., based on 182 OTUs) and the three SNP difference criterion containing only 52 SNPs is shown in Additional file 3: Table S3. The corresponding numbers of pairwise differences are summarized in Table 4 (the full information is given in the Additional file 4: Table S2). As may be expected, the average number of differentiating SNPs in pairwise comparisons is smaller within groups (i.e., the five genera *Anastrepha* (including *T*. *curvicauda*), *Bactrocera*, *Ceratitis*, *Dacus*, and *Rhagoletis* and the mixed group “Others”) than between groups (students T-Test, t < −5.3, *P* < 0.001; Additional file 3: Table S3). Pairwise comparisons among all OTUs using this panel of 52 SNPs results in an average number of 19.8 ± 4.15 differentiating SNPs. This reflects the fact that the minimal number of three differentiating SNPs, for which this panel search was performed, only occurs rarely, i.e., in only eight instances out of 16471 pairwise comparisons, and, in fact, mostly among closely related species (see Additional file 4: Table S2).

**Table 4:** **Average number of SNPs between** (**above diagonal**) **and within groups** (**on diagonal**, **bold**) **and the respective range** (**below diagonal**) **using a panel of 52 SNPs that was designed to produce an at least three SNPs difference in each pairwise comparison**

**Groups**	***Anastrepha***	***Bactrocera***	***Ceratitis***	***Dacus***	***Rhagoletis***	**Others**
*Anastrepha* (4–26)	**14**.**53**	21.58	20.05	23.40	20.07	20.68
*Bactrocera* (4–26)	4–25	**16**.**35**	18.70	21.68	19.71	19.72
*Ceratitis* (4–26)	10–28	4–26	**14**.**61**	21.38	17.62	18.21
*Dacus* (3–29)	13–29	11–32	3–30	**18**.**82**	23.64	22.53
*Rhagoletis* (3–22)	13–28	13–28	9–32	3–33	**12**.**36**	18.50
Others (3–29)	11–31	7–28	8–34	6–34	3–27	**17**.**51**

The within-group range is indicated in parentheses in the groups column. All within-group means are significantly lower than the among-group means (t-test, t < −5.3, *P* < 0.001).

SNP panels represent subsets of the total COI barcoding fragment and hence it must be assumed that using them will reduce the level of differentiation among OTUs. To assess the information content of this SNP panel of 52 SNPs we performed a Mantel test [67] on the concatenated SNP sequence vs. the full sequence of 699 bp, based on the patristic pairwise distance matrices. Despite a 13-fold reduction in markers (base pairs), the distance matrices are highly correlated (Spearman correlation, α = 0.05; r(A,B) = 0.487, *P*(two-tailed) < 0.0001) and only 6 of 92 terminal OTUs (excluding singleton groups) could not be clearly recovered in the ML tree of the 52 SNP panel. As the SNP panel is designed with a threefold redundancy, these results suggest that this diagnostic SNP panel is highly robust and well suited for practical use in diagnosing the species/OTUs of Tephritidae presented here.

## Conclusions

We show that the degree of taxon differentiation of COI in Tephritids enables differentiation at the species level in many cases. In others, two or rarely three described species are grouped into genetically identical OTUs, some of which may be caused by mistakes in taxonomic assignment. We developed COI-based SNP panels that enable discrimination of all OTUs established in this study. Because they are based on a minimum pairwise differentiation of at least three SNPs and this discrimination also includes all singleton species, these panels are highly robust and hence provide an important resource for developing identification assays for Tephritid fruit flies.

## Methods

### Samples

The species, numbers, origin, host and life stages of the samples for which we generated the sequence information for our study in-house are summarized in Table 1. We collected a total of 18 putative species of 5 genera. The number of individuals per species ranged from two to 20. Adults were identified to species level based on morphological characteristics. Larvae and pupae were tentatively identified based on the sampling location and host species.

### DNA-extraction, PCR and sequencing

Genomic DNA was extracted either by the protocol of the Gene Elute Mammalian Kit (Sigma) for tissue extraction or by means of a Proteinase K buffer (10 mM Tris HCl, 1 mM EDTA, 0.5% Tween 20, 50 μg/ml Proteinase K, pH 8.0; [68]) for sample homogenization and DNA elution, as described in [63].

The COI gene was amplified with 4 primers described in [69] and [70] in a nested design. A modified version of the forward primer TY-J-1460 (TY-J-1460mod: 5′-TACARTCTATYGCCTAAACTTCAGC-3′; [63]) and TL2-N3014 (5′-CATTGCACTAWTCTGCCATATTAGA-3′) as the reverse primer produced a fragment of ca. 1550 bp. If the sequencing reaction did not allow to join the two ends, the nested forward primer C1-J-1751 (5′-GGATCACCTGATATAGCATTCCC-3′) (alias Ron) was used with the reverse primer C1-N2353 (5′-GCTCGTGTATCAACGTCTATWCC-3′) to produce a 647 bp fragment spanning the missing part.

The polymerase chain reaction (PCR) was conducted in a total volume of 20 μl containing 1 μl of DNA extract (direct or 1/10–1/100 diluted), 6–12 μM of each oligonucleotide primer and 10 μl of HotStar Taq^TM^ MasterMix (Qiagen, Basel, Switzerland). Amplification was generally conducted using a ramping protocol on a Perkin Elmer GeneAmp 9600 (Applied Biosystems, Rotkreuz, Switzerland) or on the models Techne Genius or TC-412 (Witec, Littau, Switzerland), where an initial denaturation step at 95°C for 15 min was followed by 40–45 cycles of 40 sec at 94°C, 15 sec at 45°C, 1 min ramping to 60°C, 2 min elongation at 72°C and final extension at 72°C for 7 min. PCR products and negative controls were checked on a 1.2% agarose gel and amplification products were purified with NucleoFast® 96 PCR Plates following the protocol of the manufacturer (Macherey-Nagel, Oensingen, Switzerland). The amount of purified PCR product was measured with a NanoDrop ND-1000 spectrophotometer (Fisher Scientific AG, Wohlen, Switzerland). 20–30 ng of the DNA was used for each sequencing reaction (8 μl) together with 2 μl BigDye^TM^ Terminator Cycle Sequencing Ready Reaction Kit V.1.1 (Applied Biosystems, Rotkreuz, Switzerland) and 4 μM of each primer. Sequencing reaction products were cleaned with DyeEx™ 96 Kit (Qiagen, Basel, Switzerland) and sequenced on an ABI Prism 3130×l Genetic Analyser (Applied Biosystems, Rotkreuz, Switzerland).

### Data analysis

To complement our data set all Tephritid COI sequences available by January 2012 on GenBank (http://www.ncbi.nlm.nih.gov/genbank/) and on the “barcoding of life database” (BOLD; http://barcodinglife.com/) were downloaded and those covering at least 85% of our target fragment (i.e., >600 bp) were used (Additional file 1: Table S1, including our own sequences). Alignment and manual editing of the sequences to remove remaining gaps was performed with geneious Pro version 5.6.3 (Biomatters Ltd, Auckland, New Zealand; [71]).

#### Cluster analysis

To group individuals into “operational taxonomic units” (OTUs) we assessed the genetic relationships among the individual sequences by establishing phylogenetic trees. The evolutionary relationships for the genus trees (Figures 1-6) are inferred using the Neighbor-Joining method based on the genetic distance model of Tamura-Nei (+G+I) using 500 bootstrap replicates (bootstrap values >70% are indicated). The OTUs are established excluding singletons (explained below; see Table 3 for the thresholds of each group). Few taxa that are split when using the lower (“Including Singletons”) threshold are indicated by a square bracket and a star behind the taxon. The number of specimens per taxon is indicated in round brackets, the OTUs are given in squared brackets. The clusters of A. striata and C. capitata each contain an individual sequence that is not grouped with one or both thresholds. For the combined data set, using the corrected AIC value (Akaike Information Criterion), the software MEGA v. 5.0 [72] defined as the substitution model that fits best our data the Tamura-Nei model [73] (G = 0.68; I = 0.40; Bayesian information criterion = 85616.272). We used three different methods for tree building: i) the distance based neighbour-joining method [74] and ii) the character based maximum likelihood method as implemented in MEGA v.5.0, both using 1000 bootstrap replications [75] for calculation of node supports, and iii) Bayesian inference as implemented in the plugin MrBayes [76] in geneious Pro v.5.6.3, using *Acanthiophilus helianthi* as the outgroup. Parameters for establishing posterior probability of the Bayesian clusters were based on a chain length of 1′100′000, with a burn-in length of 100′000 and a subsampling frequency of 200.

### Taxon delimitation

To establish the optimal threshold for taxon delimitation we used the function localMinima of the SPIDER software (Species Identity and Evolution in R) v. 1.1.2 [29]. This method uses the Kimura-2-Parameter distance matrix to create a density object from which it infers the transition between intra- and inter-group (i.e., species or other genetically oligomorphic groups) distances from a depression in density of the genetic distances [29]. The advantage of this method is that it does not require a priori knowledge of the identity of the taxon, i.e., species identity. Because the occurrence of singletons, i.e., taxa represented by only one individual, may cause problems in this analysis [29,77] we run the analyses both including and “Excluding Singletons”.

OTU’s were then defined with the module “Cluster” of the software TaxonDNA/SpeciesIdentifier v.1.7.7-dev3 [4] using the thresholds established with SPIDER. Comparing the results of preliminary analyses to the terminal groups obtained by the phylogenetic trees showed a generally close correspondence between the OTU’s proposed by TaxonDNA and the terminal groups of the phylogenetic trees. However, the correspondence was best if the analysis was performed on subsets rather than the entire data set, indicating differences in threshold levels between taxonomic groups as was demonstrated before for other taxa (e.g., [78-80]. In general, genera clustered into one single or few groups. We therefore performed the analyses with six groups, the five genera for which we had more than 15 species each, i.e., *Anastrepha* (including the single group of the closely related genus *Toxotrypana*, *T*. *curvicauda*), *Bactrocera*, *Ceratitis*, *Dacus*, *Rhagoletis*, and the remaining samples were pooled as the group “Others”. Because the terminal groups were in almost all cases identical between the three methods the NJ method was used with the same parameters as described under “Cluster analysis” to build the trees for the six groups mentioned above.

#### Determining near-minimal diagnostic SNP sets

In a further step, we elaborated OTU-diagnostic sets of single nucleotide polymorphisms (SNPs). Each position in the COI alignment that was not identical in all OTUs was polymorphic and hence the corresponding nucleotide of each taxon was considered a SNP. The problem was to find the minimum number of any combination of SNPs that differentiates each taxon pair by at least one or, preferably, several SNPs. The number of possible SNP combinations that have to be checked to find the minimal number of SNPs differentiating each taxon from every other by at least, say, four SNPs, well exceeds 10^28^ and hence cannot be exhaustively explored. We therefore designed a Visual Basic script to screen one million different sets of random composition of candidate SNPs for those that fulfill the requirements (i.e., at least three or four SNPs of difference among all taxa). To enable this, we first generated a consensus sequence of each of the OTU’s established with TaxonDNA in geneious using IUB codes for ambiguous bases. The consensus sequences were then used as input files for the script. A maximum of twenty-five SNP’s of each taxon were chosen for the selection procedure, with preference given to non-silent SNP’s and to SNP’s with higher variance, i.e., the occurrence of more than one alternative base at that position of the alignment. The script is available from JEF upon request.

The agreement between the clusters obtained with the full-length barcode fragment and the concatenated sequence composed of the SNPs (of the panel designed with 3 SNP pairwise difference and “Excluding Singletons”) was tested by a Mantel test [67] implemented in the Excel addin XLstat v.2011.2.04 (Addinsoft), performed on the patristic distances of the NJ trees computed in geneious.

#### Case study: assessing the status of the R. suavis subgroup RcomGR

The *Rhagoletis suavis* group shows two distinct clusters. The first represents *R*. *suavis* sensu stricto, whereas the second is composed of three described species, *R*. *completa*, *R*. *ramosae* and *R*. *zoqui*. The occurrence of shared haplotypes in this *Rhagoletis suavis* subgroup that we call RcomGR, was tested by constructing a haplotype network based on a 1288 bp fragment of the subset of individuals for which this fragment was available in full. The minimum spanning network is embedding all minimum spanning trees computed based on the matrix of pairwise distances that is calculated between all pairs of haplotypes [81]. The network was established using the software Arlequin v. 3.1 [82] and visualized with the software HapStar v.0.6. [83].

## Abbreviations

COI: Cytochrome oxidase I; OTU: Operational taxonomic unit; SNP: Single nucleotide polymorphism.

## Competing interests

The authors declare that they have no competing interests.

## Authors’ contributions

JEF conceived the study, developed the script, performed data analyses and drafted the manuscript, LG and BF performed molecular genetic and data analyses, LG, JS, JR and MA participated in study design and manuscript writing. All authors read and approved the final manuscript.

## Supplementary Material

Additional file 1: Table S1 Samples used in this study. Indicated are the species name, the code used in our analyses, the origin of the data (this study: specimen collected and sequenced by authors; F&P: data from Frey and Pfunder, 2006; GenBank and BOLD: samples downloaded from the respective database on January 13, 2012), membership of the species in the European Plant Protection Organisation (EPPO) A1 or A2 list for European quarantine organisms. Click here for file

Additional file 2: Figure S1 Evolutionary relationships among taxa and OTUs for each taxon. Evolutionary relationships among taxa are based on 699 base pairs of the 5′-region of the mitochondrial COI gene. The relationships are inferred using the Maximum Likelihood method based on the genetic distance model of Tamura-Nei (Gamma distribution = 0.6636, with 40.03% invariable sites) using 1000 bootstrap replicates. The tree with the highest log likelihood of −33920.66 is shown. The numbered bars indicate OTUs representing groups established based on taxon delimitation levels suggested by the software SPIDER when excluding singletons (see Table 3 for the thresholds of each group). Bootstrap support values and Bayesian posterior probabilities are indicated for each OTU as Maximum Likelihood / Neighbour Joining / Bayesian posterior probability. Click here for file

Additional file 3: Table S3 SNP panel established with the singleton excluding thresholds (182 OTUs) and with a minimum of three SNPs difference in all pairwise comparisons. The table is split into the five genera *Anastrepha*, *Bactrocera*, *Ceratitis*, *Dacus* and *Rhagoletis* and in a sixth group containing all other species, but the analysis was performed over all data, i.e., the three SNPs difference holds for pairwise comparisons of all OTUs. Variable SNPs for each group are in bold. OTU Nr is the same as in the Additional file 2: Figure S1. Click here for file

Additional file 4: Table S2 The number of pairwise SNP differences between all OTUs. OTU Nr corresponds to that in Additional file 2: Figure S1. See text for explanations. Click here for file
