# Supplementary material for: Developing diagnostic SNP panels for the identification of true fruit flies (Diptera: Tephritidae) within the limits of COI-based species delimitation
Source: BMC Evol Biol. 2013 May 29;13:106. doi: 10.1186/1471-2148-13-106 (PMC3682933; doi:10.1186/1471-2148-13-106)
Supplement: Additional file 2: Figure S1 — Evolutionary relationships among taxa and OTUs for each taxon. Evolutionary relationships among taxa are based on 699 base pairs of the 5′-region of the mitochondrial COI gene. The relationships are inferred using the Maximum Likelihood method based on the genetic distance model of Tamura-Nei (Gamma distribution = 0.6636, with 40.03% invariable sites) using 1000 bootstrap replicates. The tree with the highest log likelihood of −33920.66 is shown. The numbered bars indicate OTUs representing groups established based on taxon delimitation levels suggested by the software SPIDER when excluding singletons (see Table 3 for the thresholds of each group). Bootstrap support values and Bayesian posterior probabilities are indicated for each OTU as Maximum Likelihood / Neighbour Joining / Bayesian posterior probability. [file 1471-2148-13-106-S2.pdf]

Supplementary Figure 1

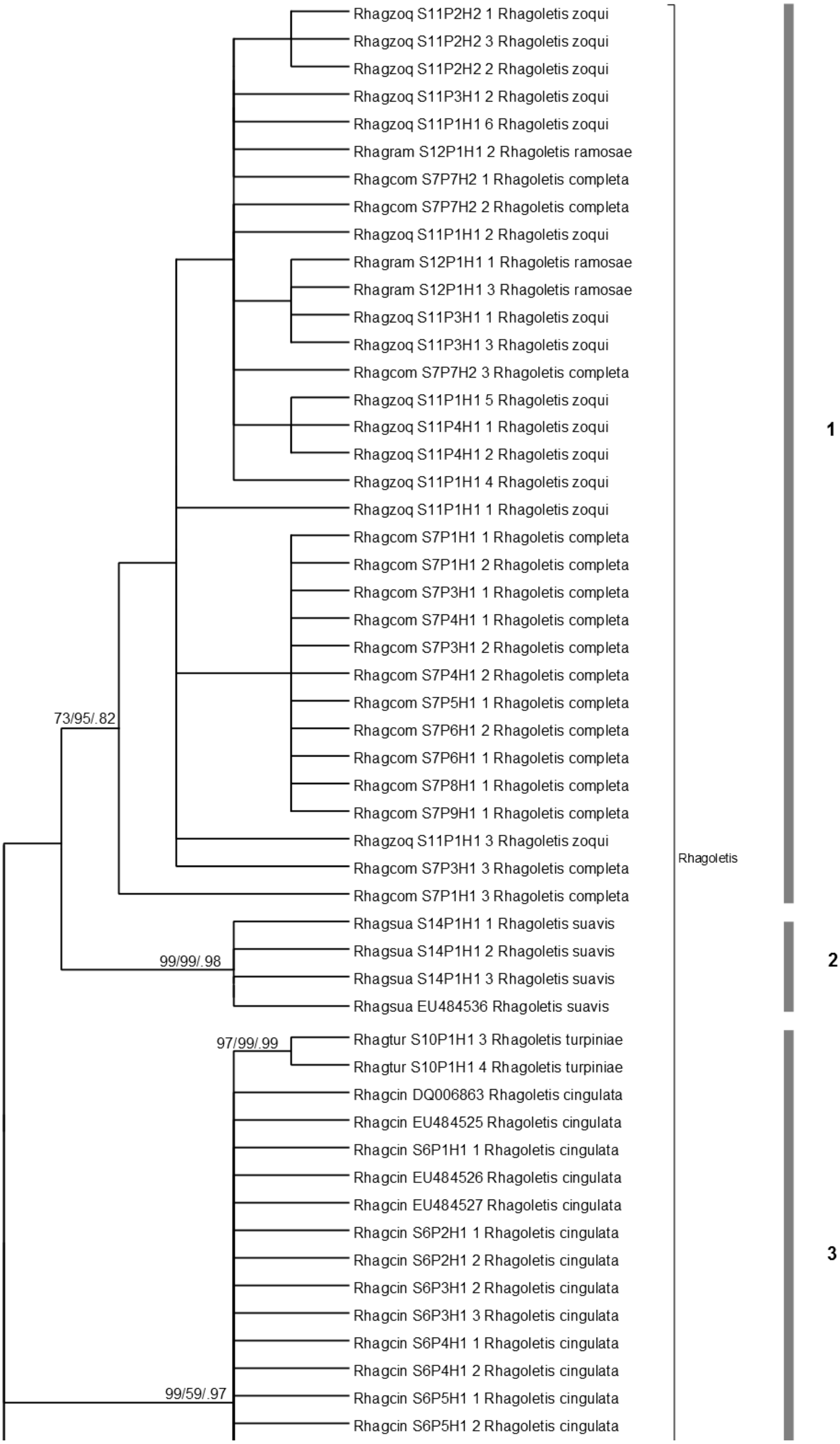

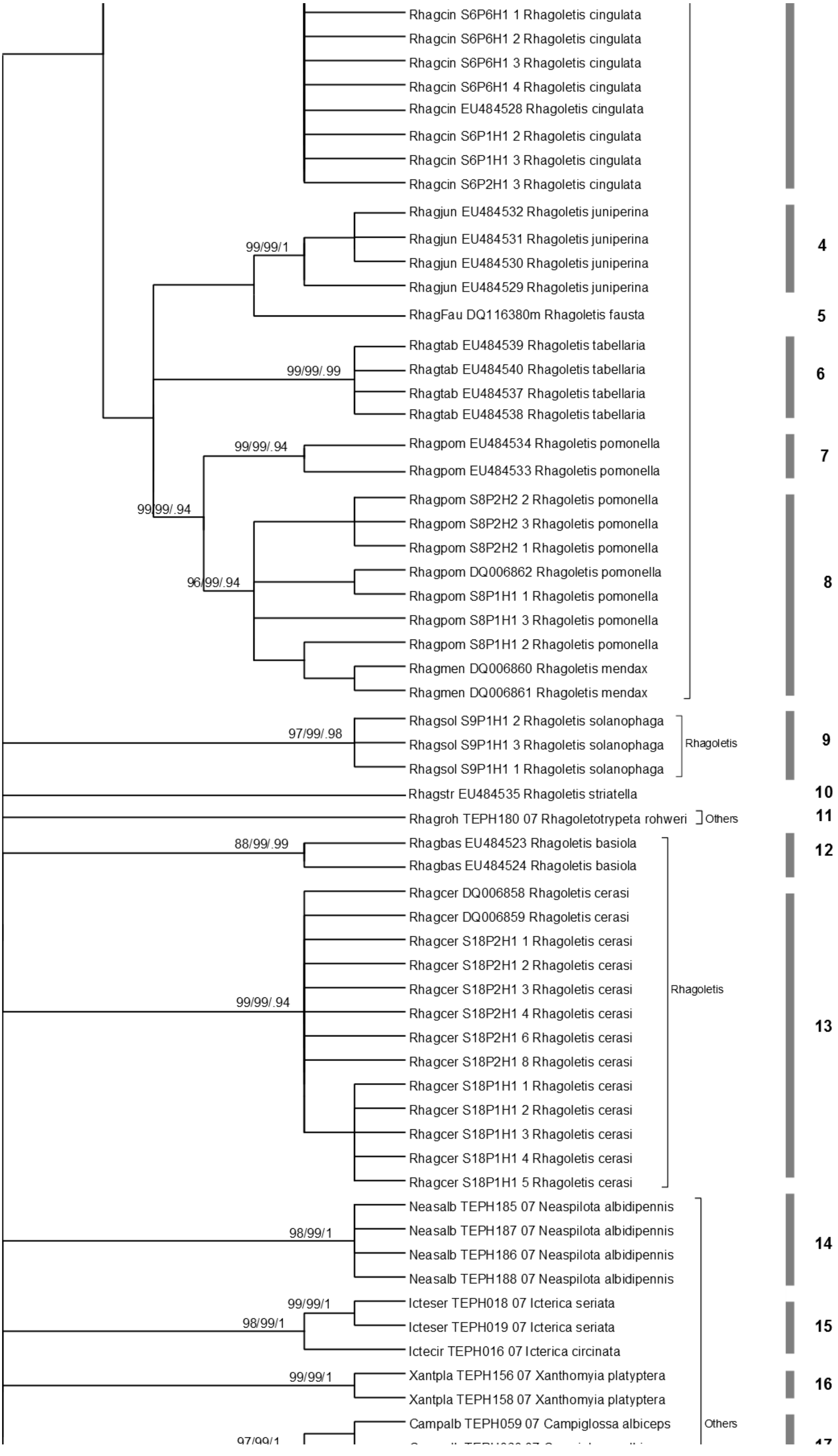
Rhagcin S6P6H1 1 Rhagoletis cingulata
Rhagcin S6P6H1 2 Rhagoletis cingulata
Rhagcin S6P6H1 3 Rhagoletis cingulata
Rhagcin S6P6H1 4 Rhagoletis cingulata
Rhagcin EU484528 Rhagoletis cingulata
Rhagcin S6P1H1 2 Rhagoletis cingulata
Rhagcin S6P1H1 3 Rhagoletis cingulata
Rhagcin S6P2H1 3 Rhagoletis cingulata
99/99/1
Rhagjun EU484532 Rhagoletis juniperina
Rhagjun EU484531 Rhagoletis juniperina
Rhagjun EU484530 Rhagoletis juniperina
Rhagjun EU484529 Rhagoletis juniperina
4
RhagFau DQ116380m Rhagoletis fausta
5
99/99/.99
Rhagtab EU484539 Rhagoletis tabellaria
Rhagtab EU484540 Rhagoletis tabellaria
Rhagtab EU484537 Rhagoletis tabellaria
Rhagtab EU484538 Rhagoletis tabellaria
6
99/99/.94
Rhagpom EU484534 Rhagoletis pomonella
Rhagpom EU484533 Rhagoletis pomonella
7
99/99/.94
96/99/.94
Rhagpom S8P2H2 2 Rhagoletis pomonella
Rhagpom S8P2H2 3 Rhagoletis pomonella
Rhagpom S8P2H2 1 Rhagoletis pomonella
Rhagpom DQ006862 Rhagoletis pomonella
Rhagpom S8P1H1 1 Rhagoletis pomonella
Rhagpom S8P1H1 3 Rhagoletis pomonella
Rhagpom S8P1H1 2 Rhagoletis pomonella
Rhagmen DQ006860 Rhagoletis mendax
Rhagmen DQ006861 Rhagoletis mendax
8
97/99/.98
Rhagsol S9P1H1 2 Rhagoletis solanophaga
Rhagsol S9P1H1 3 Rhagoletis solanophaga
Rhagsol S9P1H1 1 Rhagoletis solanophaga
Rhagoletis
9
Rhagstr EU484535 Rhagoletis striatella
10
Rhagroh TEPH180 07 Rhagoletotrypeta rohweri
Others
11
88/99/.99
Rhagbas EU484523 Rhagoletis basiola
Rhagbas EU484524 Rhagoletis basiola
12
99/99/.94
Rhagcer DQ006858 Rhagoletis cerasi
Rhagcer DQ006859 Rhagoletis cerasi
Rhagcer S18P2H1 1 Rhagoletis cerasi
Rhagcer S18P2H1 2 Rhagoletis cerasi
Rhagcer S18P2H1 3 Rhagoletis cerasi
Rhagcer S18P2H1 4 Rhagoletis cerasi
Rhagcer S18P2H1 6 Rhagoletis cerasi
Rhagcer S18P2H1 8 Rhagoletis cerasi
Rhagcer S18P1H1 1 Rhagoletis cerasi
Rhagcer S18P1H1 2 Rhagoletis cerasi
Rhagcer S18P1H1 3 Rhagoletis cerasi
Rhagcer S18P1H1 4 Rhagoletis cerasi
Rhagcer S18P1H1 5 Rhagoletis cerasi
Rhagoletis
13
98/99/1
Neasalb TEPH185 07 Neaspilota albidipennis
Neasalb TEPH187 07 Neaspilota albidipennis
Neasalb TEPH186 07 Neaspilota albidipennis
Neasalb TEPH188 07 Neaspilota albidipennis
14
99/99/1
98/99/1
Icteser TEPH018 07 Icterica seriata
Icteser TEPH019 07 Icterica seriata
Ictecir TEPH016 07 Icterica circinata
15
99/99/1
Xantpla TEPH156 07 Xanthomyia platyptera
Xantpla TEPH158 07 Xanthomyia platyptera
16
97/99/1
Campalb TEPH059 07 Campiglossa albiceps
Others

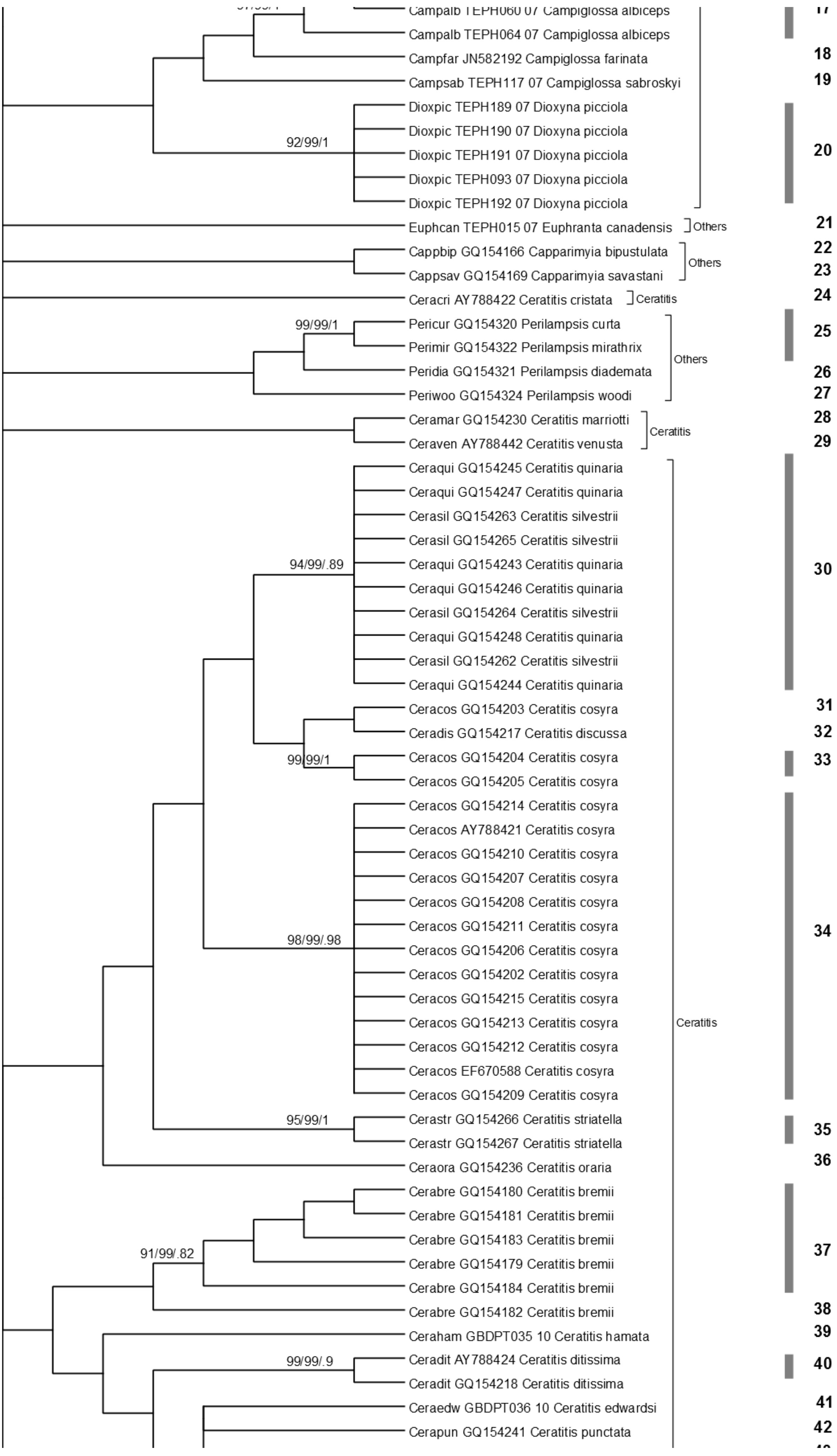

Campalb TEPH060 07 Campiglossa albiceps
Campalb TEPH064 07 Campiglossa albiceps
Campfar JN582192 Campiglossa farinata
Campsab TEPH117 07 Campiglossa sabroskyi
92/99/1
Dioxpic TEPH189 07 Dioxyna picciola
Dioxpic TEPH190 07 Dioxyna picciola
Dioxpic TEPH191 07 Dioxyna picciola
Dioxpic TEPH093 07 Dioxyna picciola
Dioxpic TEPH192 07 Dioxyna picciola
Euphcan TEPH015 07 Euphranta canadensis
Others
Cappbip GQ154166 Capparimyia bipustulata
Cappsav GQ154169 Capparimyia savastani
Others
Ceracri AY788422 Ceratitis cristata
Ceratitis
99/99/1
Pericur GQ154320 Perilampsis curta
Perimir GQ154322 Perilampsis mirathrix
Peridia GQ154321 Perilampsis diademata
Periwoo GQ154324 Perilampsis woodi
Others
Ceramar GQ154230 Ceratitis marriotti
Ceraven AY788442 Ceratitis venusta
Ceratitis
94/99/.89
Ceraqui GQ154245 Ceratitis quinaria
Ceraqui GQ154247 Ceratitis quinaria
Cerasil GQ154263 Ceratitis silvestrii
Cerasil GQ154265 Ceratitis silvestrii
Ceraqui GQ154243 Ceratitis quinaria
Ceraqui GQ154246 Ceratitis quinaria
Cerasil GQ154264 Ceratitis silvestrii
Ceraqui GQ154248 Ceratitis quinaria
Cerasil GQ154262 Ceratitis silvestrii
Ceraqui GQ154244 Ceratitis quinaria
Ceracos GQ154203 Ceratitis cosyra
Ceradis GQ154217 Ceratitis discussa
99/99/1
Ceracos GQ154204 Ceratitis cosyra
Ceracos GQ154205 Ceratitis cosyra
98/99/.98
Ceracos GQ154214 Ceratitis cosyra
Ceracos AY788421 Ceratitis cosyra
Ceracos GQ154210 Ceratitis cosyra
Ceracos GQ154207 Ceratitis cosyra
Ceracos GQ154208 Ceratitis cosyra
Ceracos GQ154211 Ceratitis cosyra
Ceracos GQ154206 Ceratitis cosyra
Ceracos GQ154202 Ceratitis cosyra
Ceracos GQ154215 Ceratitis cosyra
Ceracos GQ154213 Ceratitis cosyra
Ceracos GQ154212 Ceratitis cosyra
Ceracos EF670588 Ceratitis cosyra
Ceracos GQ154209 Ceratitis cosyra
95/99/1
Cerastr GQ154266 Ceratitis striatella
Cerastr GQ154267 Ceratitis striatella
Ceraora GQ154236 Ceratitis oraria
91/99/.82
Cerabre GQ154180 Ceratitis bremii
Cerabre GQ154181 Ceratitis bremii
Cerabre GQ154183 Ceratitis bremii
Cerabre GQ154179 Ceratitis bremii
Cerabre GQ154184 Ceratitis bremii
Cerabre GQ154182 Ceratitis bremii
Ceraham GBDPT035 10 Ceratitis hamata
99/99/.9
Ceradit AY788424 Ceratitis ditissima
Ceradit GQ154218 Ceratitis ditissima
Ceraedw GBDPT036 10 Ceratitis edwardsi
Cerapun GQ154241 Ceratitis punctata
Ceratitis
18
19
20
21
22
23
24
25
26
27
28
29
30
31
32
33
34
35
36
37
38
39
40
41
42

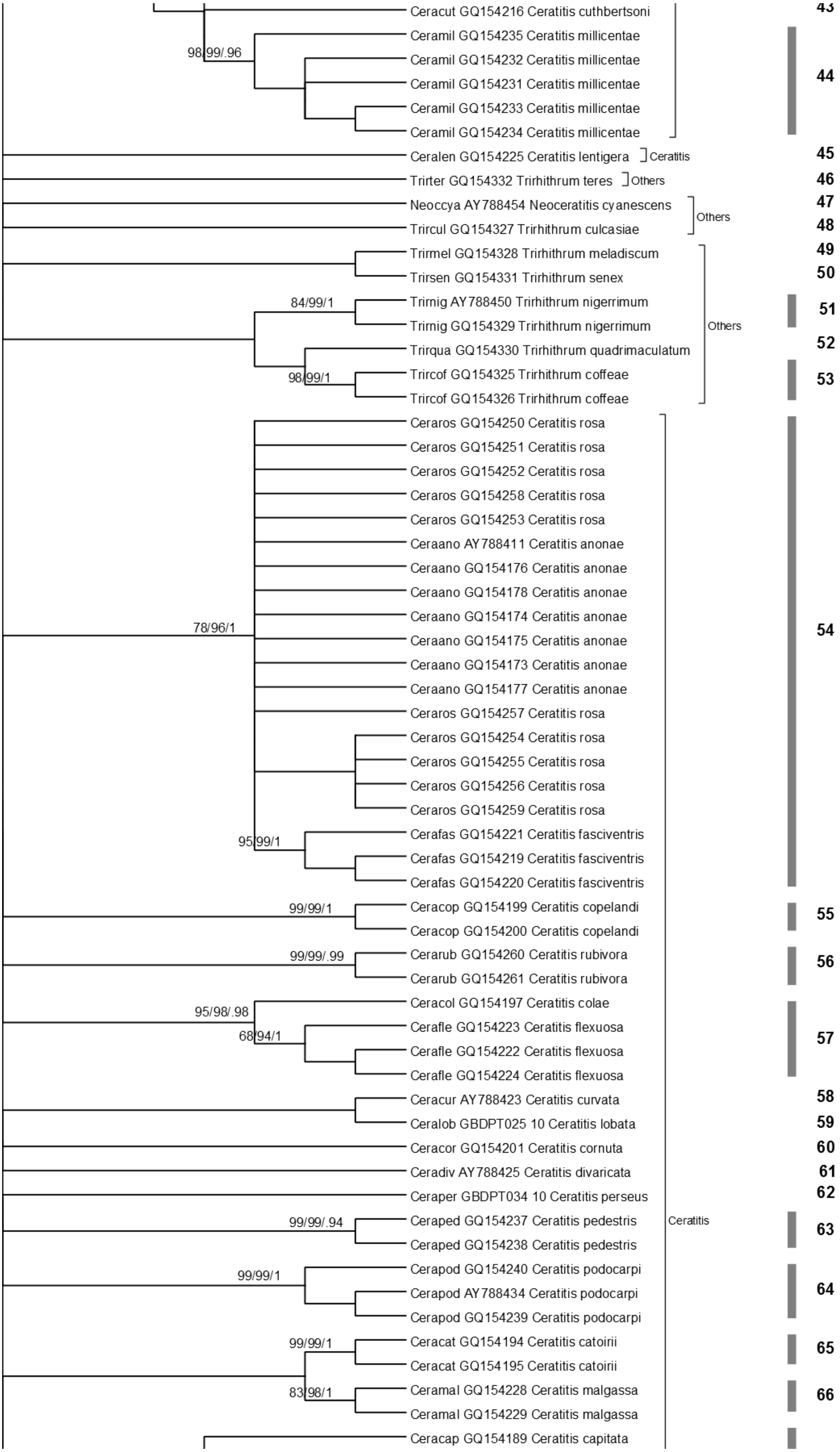
Ceracut GQ154216 Ceratitis cuthbertsoni
43
98/99/.96
Ceramil GQ154235 Ceratitis millicentae
Ceramil GQ154232 Ceratitis millicentae
Ceramil GQ154231 Ceratitis millicentae
Ceramil GQ154233 Ceratitis millicentae
Ceramil GQ154234 Ceratitis millicentae
44
Ceralen GQ154225 Ceratitis lentigera
Ceratitis
45
Trirter GQ154332 Trirhithrum teres
Others
46
Neoccya AY788454 Neoceratitis cyanescens
47
Tricul GQ154327 Trirhithrum culcasiae
Others
48
Trirmel GQ154328 Trirhithrum meladiscum
49
Trirsen GQ154331 Trirhithrum senex
50
84/99/1
Trirnig AY788450 Trirhithrum nigerrimum
Trirnig GQ154329 Trirhithrum nigerrimum
51
Others
Trirqua GQ154330 Trirhithrum quadrimaculatum
52
98/99/1
Trircof GQ154325 Trirhithrum coffeae
Trircof GQ154326 Trirhithrum coffeae
53
Ceraros GQ154250 Ceratitis rosa
Ceraros GQ154251 Ceratitis rosa
Ceraros GQ154252 Ceratitis rosa
Ceraros GQ154258 Ceratitis rosa
Ceraros GQ154253 Ceratitis rosa
Ceraano AY788411 Ceratitis anonae
Ceraano GQ154176 Ceratitis anonae
Ceraano GQ154178 Ceratitis anonae
Ceraano GQ154174 Ceratitis anonae
78/96/1
Ceraano GQ154175 Ceratitis anonae
54
Ceraano GQ154173 Ceratitis anonae
Ceraano GQ154177 Ceratitis anonae
Ceraros GQ154257 Ceratitis rosa
Ceraros GQ154254 Ceratitis rosa
Ceraros GQ154255 Ceratitis rosa
Ceraros GQ154256 Ceratitis rosa
Ceraros GQ154259 Ceratitis rosa
95/99/1
Cerafas GQ154221 Ceratitis fasciventris
Cerafas GQ154219 Ceratitis fasciventris
Cerafas GQ154220 Ceratitis fasciventris
99/99/1
Ceracop GQ154199 Ceratitis copelandi
55
Ceracop GQ154200 Ceratitis copelandi
99/99/.99
Cerarub GQ154260 Ceratitis rubivora
56
Cerarub GQ154261 Ceratitis rubivora
95/98/.98
Ceracol GQ154197 Ceratitis colae
Cerafle GQ154223 Ceratitis flexuosa
68/94/1
Cerafle GQ154222 Ceratitis flexuosa
57
Cerafle GQ154224 Ceratitis flexuosa
Ceracur AY788423 Ceratitis curvata
58
Ceralob GBDPT025 10 Ceratitis lobata
59
Ceracor GQ154201 Ceratitis cornuta
60
Ceradiv AY788425 Ceratitis divaricata
61
Ceraper GBDPT034 10 Ceratitis perseus
62
99/99/.94
Ceraped GQ154237 Ceratitis pedestris
Ceratitis
63
Ceraped GQ154238 Ceratitis pedestris
99/99/1
Cerapod GQ154240 Ceratitis podocarpi
Cerapod AY788434 Ceratitis podocarpi
64
Cerapod GQ154239 Ceratitis podocarpi
99/99/1
Ceracat GQ154194 Ceratitis catoirii
65
Ceracat GQ154195 Ceratitis catoirii
83/98/1
Ceramal GQ154228 Ceratitis malgassa
66
Ceramal GQ154229 Ceratitis malgassa
Ceracap GQ154189 Ceratitis capitata

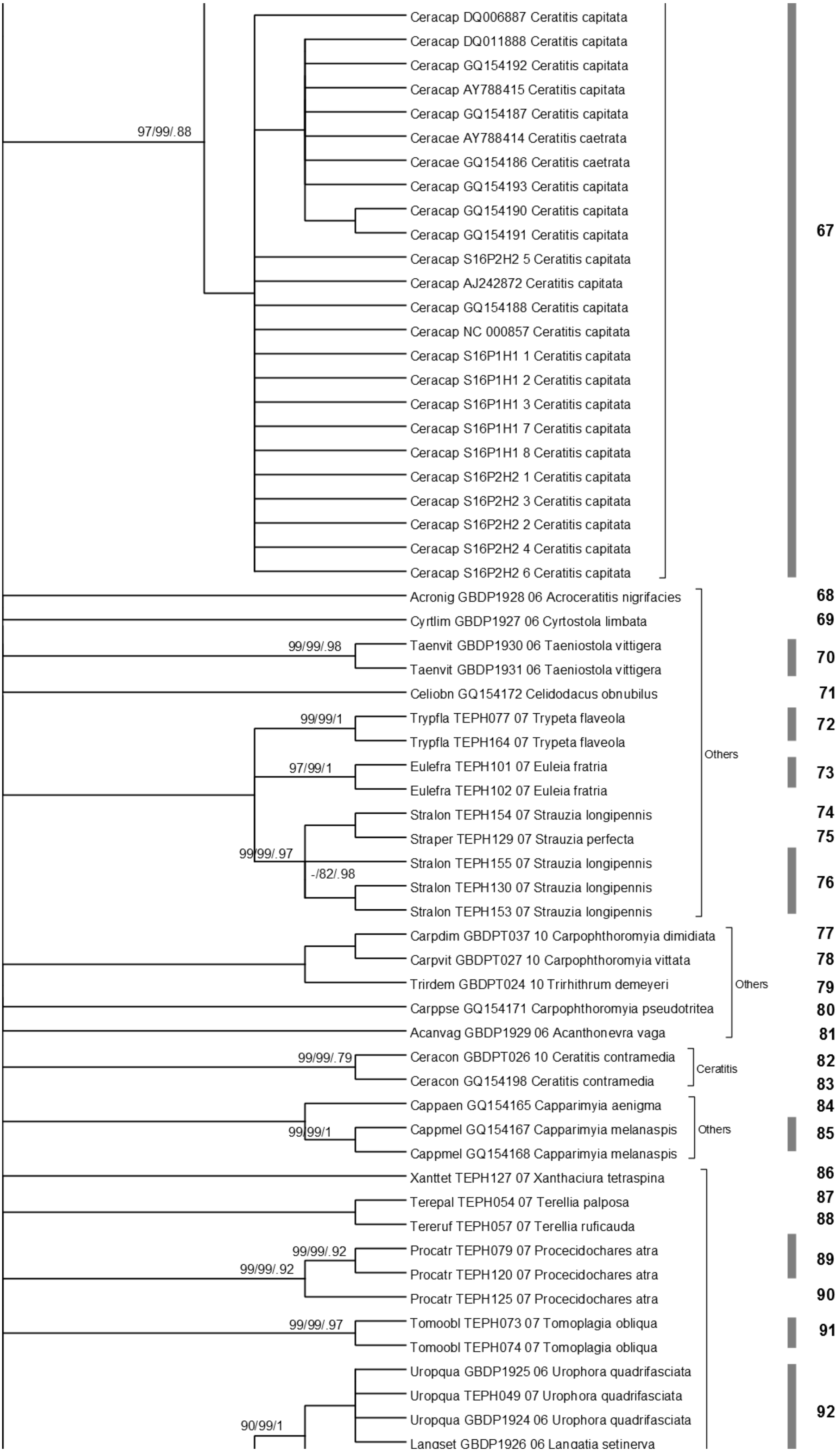

97/99/.88
Ceracap DQ006887 Ceratitis capitata
Ceracap DQ011888 Ceratitis capitata
Ceracap GQ154192 Ceratitis capitata
Ceracap AY788415 Ceratitis capitata
Ceracap GQ154187 Ceratitis capitata
Ceracae AY788414 Ceratitis caetrata
Ceracae GQ154186 Ceratitis caetrata
Ceracap GQ154193 Ceratitis capitata
Ceracap GQ154190 Ceratitis capitata
Ceracap GQ154191 Ceratitis capitata
Ceracap S16P2H2 5 Ceratitis capitata
Ceracap AJ242872 Ceratitis capitata
Ceracap GQ154188 Ceratitis capitata
Ceracap NC 000857 Ceratitis capitata
Ceracap S16P1H1 1 Ceratitis capitata
Ceracap S16P1H1 2 Ceratitis capitata
Ceracap S16P1H1 3 Ceratitis capitata
Ceracap S16P1H1 7 Ceratitis capitata
Ceracap S16P1H1 8 Ceratitis capitata
Ceracap S16P2H2 1 Ceratitis capitata
Ceracap S16P2H2 3 Ceratitis capitata
Ceracap S16P2H2 2 Ceratitis capitata
Ceracap S16P2H2 4 Ceratitis capitata
Ceracap S16P2H2 6 Ceratitis capitata
67
Acronig GBDP1928 06 Acroceratitis nigrifacies
68
Cyrtlim GBDP1927 06 Cyrtostola limbata
69
99/99/.98
Taenvit GBDP1930 06 Taeniostola vittigera
Taenvit GBDP1931 06 Taeniostola vittigera
70
Celiobn GQ154172 Celidodacus obnubilus
71
99/99/1
Trypfla TEPH077 07 Trypeta flaveola
Trypfla TEPH164 07 Trypeta flaveola
72
97/99/1
Eulefra TEPH101 07 Euleia fratria
Eulefra TEPH102 07 Euleia fratria
73
Stralon TEPH154 07 Strauzia longipennis
74
Straper TEPH129 07 Strauzia perfecta
75
99/99/.97
Stralon TEPH155 07 Strauzia longipennis
-/82/.98
Stralon TEPH130 07 Strauzia longipennis
Stralon TEPH153 07 Strauzia longipennis
76
Others
Carpdim GBDPT037 10 Carpophthoromyia dimidiata
77
Carpvit GBDPT027 10 Carpophthoromyia vittata
78
Trirdem GBDPT024 10 Trirhithrum demeyeri
79
Carppse GQ154171 Carpophthoromyia pseudotritea
80
Acanvag GBDP1929 06 Acanthonevra vaga
81
Others
99/99/.79
Ceracon GBDPT026 10 Ceratitis contramedia
82
Ceracon GQ154198 Ceratitis contramedia
83
Ceratitis
Cappaen GQ154165 Capparimyia aenigma
84
99/99/1
Cappmel GQ154167 Capparimyia melanaspis
85
Cappmel GQ154168 Capparimyia melanaspis
Others
Xanttet TEPH127 07 Xanthaciura tetraspina
86
Terepal TEPH054 07 Terellia palposa
87
Tereruf TEPH057 07 Terellia ruficauda
88
99/99/.92
Procatr TEPH079 07 Procecidochares atra
99/99/.92
Procatr TEPH120 07 Procecidochares atra
89
Procatr TEPH125 07 Procecidochares atra
90
99/99/.97
Tomoobl TEPH073 07 Tomoplagia obliqua
Tomoobl TEPH074 07 Tomoplagia obliqua
91
Uropqua GBDP1925 06 Urophora quadrifasciata
Uropqua TEPH049 07 Urophora quadrifasciata
Uropqua GBDP1924 06 Urophora quadrifasciata
90/99/1
Langset GBDP1926 06 Langatia setinerva
92

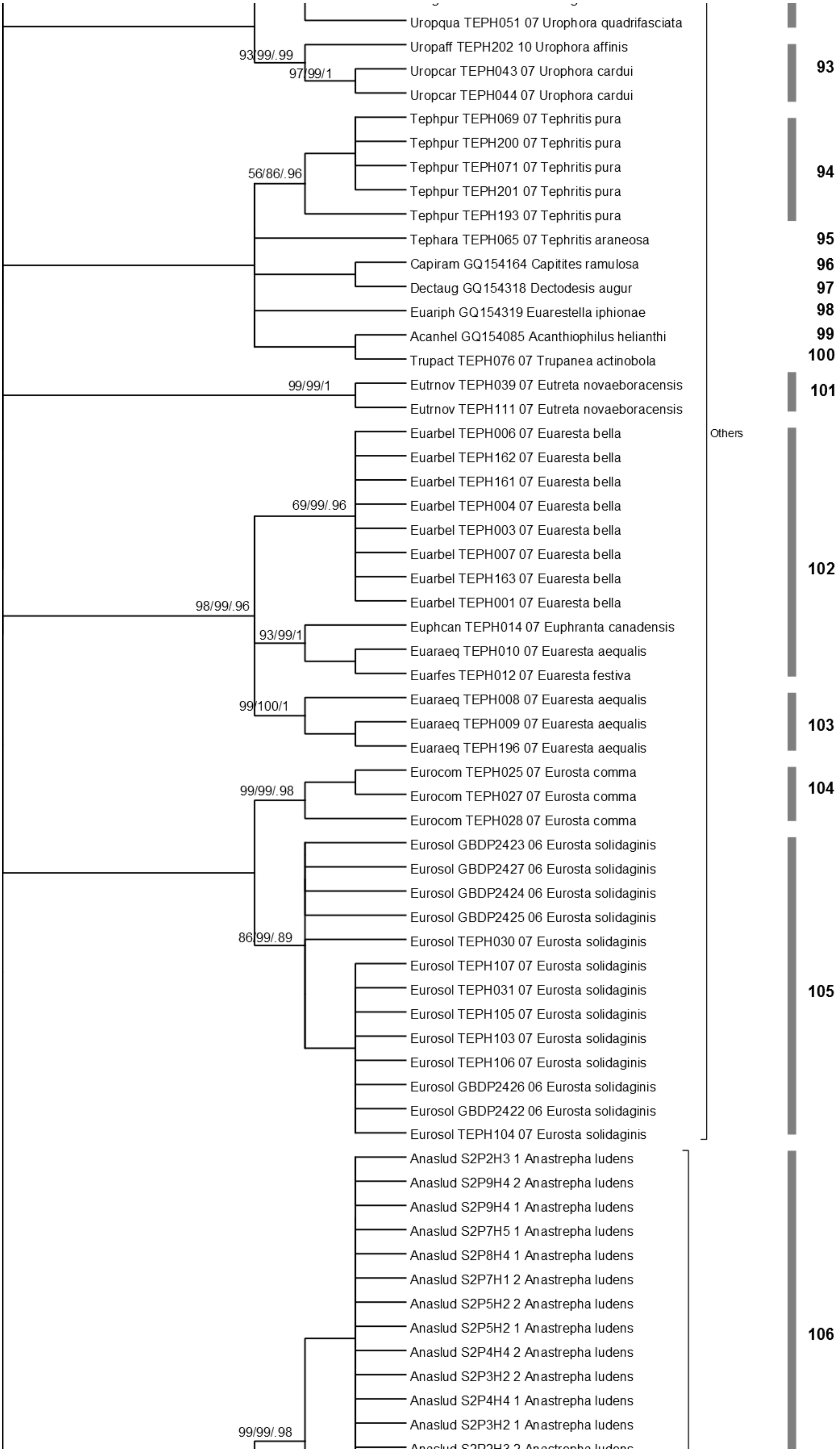
Uropqua TEPH051 07 Urophora quadrifasciata
93/99/.99
Uropaff TEPH202 10 Urophora affinis
97/99/1
Uropcar TEPH043 07 Urophora cardui
Uropcar TEPH044 07 Urophora cardui
93
Tephpur TEPH069 07 Tephritis pura
Tephpur TEPH200 07 Tephritis pura
56/86/.96
Tephpur TEPH071 07 Tephritis pura
Tephpur TEPH201 07 Tephritis pura
Tephpur TEPH193 07 Tephritis pura
94
Tephara TEPH065 07 Tephritis araneosa
95
Capiram GQ154164 Capitites ramulosa
96
Dectaug GQ154318 Dectodesis augur
97
Euariph GQ154319 Euarestella iphionae
98
Acanhel GQ154085 Acanthiophilus helianthi
99
Trupact TEPH076 07 Trupanea actinobola
100
99/99/1
Eutrnov TEPH039 07 Eutreta novaeboracensis
Eutrnov TEPH111 07 Eutreta novaeboracensis
101
Others
Euarbel TEPH006 07 Euaresta bella
Euarbel TEPH162 07 Euaresta bella
Euarbel TEPH161 07 Euaresta bella
69/99/.96
Euarbel TEPH004 07 Euaresta bella
Euarbel TEPH003 07 Euaresta bella
Euarbel TEPH007 07 Euaresta bella
Euarbel TEPH163 07 Euaresta bella
98/99/.96
Euarbel TEPH001 07 Euaresta bella
93/99/1
Euphcan TEPH014 07 Euphranta canadensis
Euaraeq TEPH010 07 Euaresta aequalis
Euarfes TEPH012 07 Euaresta festiva
102
99/100/1
Euaraeq TEPH008 07 Euaresta aequalis
Euaraeq TEPH009 07 Euaresta aequalis
Euaraeq TEPH196 07 Euaresta aequalis
103
99/99/.98
Eurocom TEPH025 07 Eurosta comma
Eurocom TEPH027 07 Eurosta comma
Eurocom TEPH028 07 Eurosta comma
104
Eurosol GBDP2423 06 Eurosta solidaginis
Eurosol GBDP2427 06 Eurosta solidaginis
Eurosol GBDP2424 06 Eurosta solidaginis
Eurosol GBDP2425 06 Eurosta solidaginis
86/99/.89
Eurosol TEPH030 07 Eurosta solidaginis
Eurosol TEPH107 07 Eurosta solidaginis
Eurosol TEPH031 07 Eurosta solidaginis
Eurosol TEPH105 07 Eurosta solidaginis
Eurosol TEPH103 07 Eurosta solidaginis
Eurosol TEPH106 07 Eurosta solidaginis
Eurosol GBDP2426 06 Eurosta solidaginis
Eurosol GBDP2422 06 Eurosta solidaginis
Eurosol TEPH104 07 Eurosta solidaginis
105
Anaslud S2P2H3 1 Anastrepha ludens
Anaslud S2P9H4 2 Anastrepha ludens
Anaslud S2P9H4 1 Anastrepha ludens
Anaslud S2P7H5 1 Anastrepha ludens
Anaslud S2P8H4 1 Anastrepha ludens
Anaslud S2P7H1 2 Anastrepha ludens
Anaslud S2P5H2 2 Anastrepha ludens
Anaslud S2P5H2 1 Anastrepha ludens
Anaslud S2P4H4 2 Anastrepha ludens
Anaslud S2P3H2 2 Anastrepha ludens
Anaslud S2P4H4 1 Anastrepha ludens
Anaslud S2P3H2 1 Anastrepha ludens
99/99/.98
106

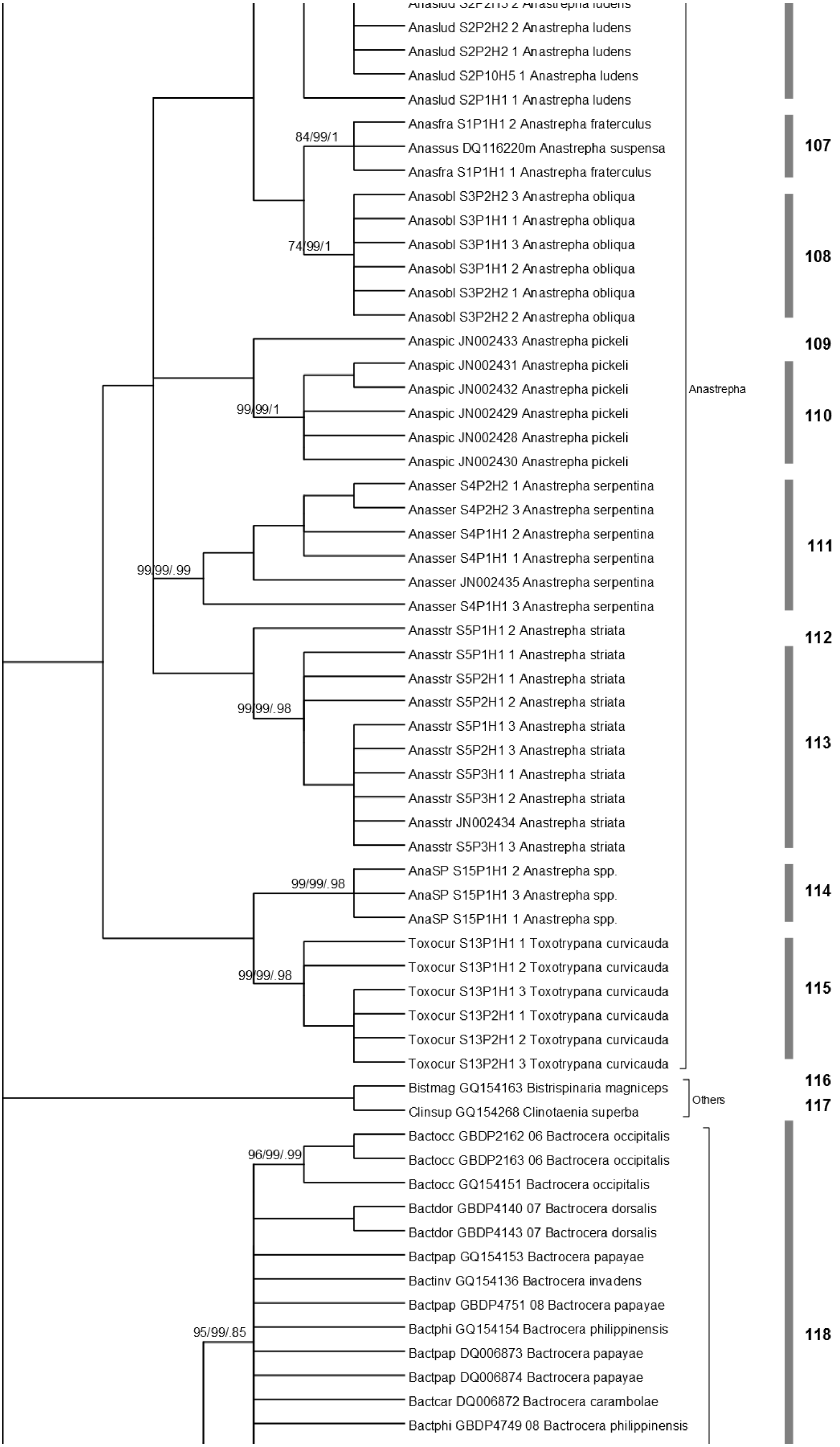

Anaslud S2P2H2 2 Anastrepha ludens
Anaslud S2P2H2 1 Anastrepha ludens
Anaslud S2P10H5 1 Anastrepha ludens
Anaslud S2P1H1 1 Anastrepha ludens
84/99/1
Anasfra S1P1H1 2 Anastrepha fraterculus
Anassus DQ116220m Anastrepha suspensa
Anasfra S1P1H1 1 Anastrepha fraterculus
107
Anasobl S3P2H2 3 Anastrepha obliqua
Anasobl S3P1H1 1 Anastrepha obliqua
74/99/1
Anasobl S3P1H1 3 Anastrepha obliqua
Anasobl S3P1H1 2 Anastrepha obliqua
Anasobl S3P2H2 1 Anastrepha obliqua
Anasobl S3P2H2 2 Anastrepha obliqua
108
Anaspic JN002433 Anastrepha pickeli
109
Anaspic JN002431 Anastrepha pickeli
Anaspic JN002432 Anastrepha pickeli
Anastrepha
99/99/1
Anaspic JN002429 Anastrepha pickeli
Anaspic JN002428 Anastrepha pickeli
Anaspic JN002430 Anastrepha pickeli
110
Anasser S4P2H2 1 Anastrepha serpentina
Anasser S4P2H2 3 Anastrepha serpentina
Anasser S4P1H1 2 Anastrepha serpentina
Anasser S4P1H1 1 Anastrepha serpentina
99/99/.99
Anasser JN002435 Anastrepha serpentina
Anasser S4P1H1 3 Anastrepha serpentina
111
Anasstr S5P1H1 2 Anastrepha striata
112
Anasstr S5P1H1 1 Anastrepha striata
Anasstr S5P2H1 1 Anastrepha striata
99/99/.98
Anasstr S5P2H1 2 Anastrepha striata
Anasstr S5P1H1 3 Anastrepha striata
Anasstr S5P2H1 3 Anastrepha striata
Anasstr S5P3H1 1 Anastrepha striata
Anasstr S5P3H1 2 Anastrepha striata
Anasstr JN002434 Anastrepha striata
Anasstr S5P3H1 3 Anastrepha striata
113
99/99/.98
AnaSP S15P1H1 2 Anastrepha spp.
AnaSP S15P1H1 3 Anastrepha spp.
AnaSP S15P1H1 1 Anastrepha spp.
114
Toxocur S13P1H1 1 Toxotrypana curvicauda
99/99/.98
Toxocur S13P1H1 2 Toxotrypana curvicauda
Toxocur S13P1H1 3 Toxotrypana curvicauda
Toxocur S13P2H1 1 Toxotrypana curvicauda
Toxocur S13P2H1 2 Toxotrypana curvicauda
Toxocur S13P2H1 3 Toxotrypana curvicauda
115
Bistmag GQ154163 Bistrispinaria magniceps
116
Others
Clinsup GQ154268 Clinotaenia superba
117
96/99/.99
Bactocc GBDP2162 06 Bactrocera occipitalis
Bactocc GBDP2163 06 Bactrocera occipitalis
Bactocc GQ154151 Bactrocera occipitalis
Bactdor GBDP4140 07 Bactrocera dorsalis
Bactdor GBDP4143 07 Bactrocera dorsalis
Bactpap GQ154153 Bactrocera papayae
Bactinv GQ154136 Bactrocera invadens
Bactpap GBDP4751 08 Bactrocera papayae
95/99/.85
Bactphi GQ154154 Bactrocera philippinensis
Bactpap DQ006873 Bactrocera papayae
Bactpap DQ006874 Bactrocera papayae
Bactcar DQ006872 Bactrocera carambolae
Bactphi GBDP4749 08 Bactrocera philippinensis
118

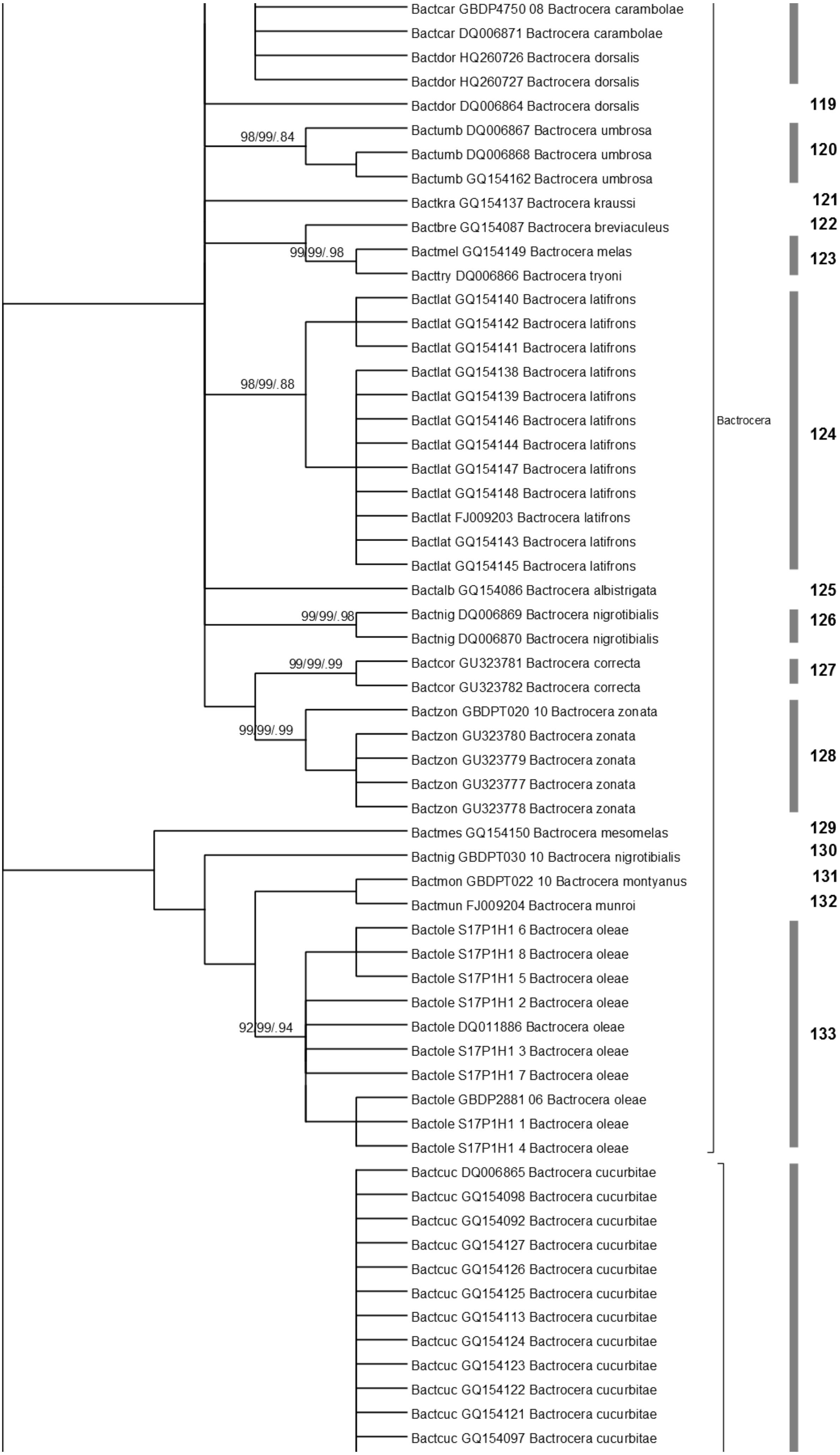
Bactcar GBDP4750 08 Bactrocera carambolae
Bactcar DQ006871 Bactrocera carambolae
Bactdor HQ260726 Bactrocera dorsalis
Bactdor HQ260727 Bactrocera dorsalis
Bactdor DQ006864 Bactrocera dorsalis
119
98/99/.84
Bactumb DQ006867 Bactrocera umbrosa
Bactumb DQ006868 Bactrocera umbrosa
Bactumb GQ154162 Bactrocera umbrosa
120
Bactkra GQ154137 Bactrocera kraussi
121
Bactbre GQ154087 Bactrocera breviaculeus
122
99/99/.98
Bactmel GQ154149 Bactrocera melas
Bacttry DQ006866 Bactrocera tryoni
123
Bactlat GQ154140 Bactrocera latifrons
Bactlat GQ154142 Bactrocera latifrons
Bactlat GQ154141 Bactrocera latifrons
98/99/.88
Bactlat GQ154138 Bactrocera latifrons
Bactlat GQ154139 Bactrocera latifrons
Bactlat GQ154146 Bactrocera latifrons
Bactlat GQ154144 Bactrocera latifrons
Bactlat GQ154147 Bactrocera latifrons
Bactlat GQ154148 Bactrocera latifrons
Bactlat FJ009203 Bactrocera latifrons
Bactlat GQ154143 Bactrocera latifrons
Bactlat GQ154145 Bactrocera latifrons
Bactrocera
124
Bactalb GQ154086 Bactrocera albistrigata
125
99/99/.98
Bactnig DQ006869 Bactrocera nigrotibialis
Bactnig DQ006870 Bactrocera nigrotibialis
126
99/99/.99
Bactcor GU323781 Bactrocera correcta
Bactcor GU323782 Bactrocera correcta
127
99/99/.99
Bactzon GBDPT020 10 Bactrocera zonata
Bactzon GU323780 Bactrocera zonata
Bactzon GU323779 Bactrocera zonata
Bactzon GU323777 Bactrocera zonata
Bactzon GU323778 Bactrocera zonata
128
Bactmes GQ154150 Bactrocera mesomelas
129
Bactnig GBDPT030 10 Bactrocera nigrotibialis
130
Bactmon GBDPT022 10 Bactrocera montyanus
131
Bactmun FJ009204 Bactrocera munroi
132
Bactole S17P1H1 6 Bactrocera oleae
Bactole S17P1H1 8 Bactrocera oleae
Bactole S17P1H1 5 Bactrocera oleae
Bactole S17P1H1 2 Bactrocera oleae
92/99/.94
Bactole DQ011886 Bactrocera oleae
Bactole S17P1H1 3 Bactrocera oleae
Bactole S17P1H1 7 Bactrocera oleae
Bactole GBDP2881 06 Bactrocera oleae
Bactole S17P1H1 1 Bactrocera oleae
Bactole S17P1H1 4 Bactrocera oleae
133
Bactcuc DQ006865 Bactrocera cucurbitae
Bactcuc GQ154098 Bactrocera cucurbitae
Bactcuc GQ154092 Bactrocera cucurbitae
Bactcuc GQ154127 Bactrocera cucurbitae
Bactcuc GQ154126 Bactrocera cucurbitae
Bactcuc GQ154125 Bactrocera cucurbitae
Bactcuc GQ154113 Bactrocera cucurbitae
Bactcuc GQ154124 Bactrocera cucurbitae
Bactcuc GQ154123 Bactrocera cucurbitae
Bactcuc GQ154122 Bactrocera cucurbitae
Bactcuc GQ154121 Bactrocera cucurbitae
Bactcuc GQ154097 Bactrocera cucurbitae

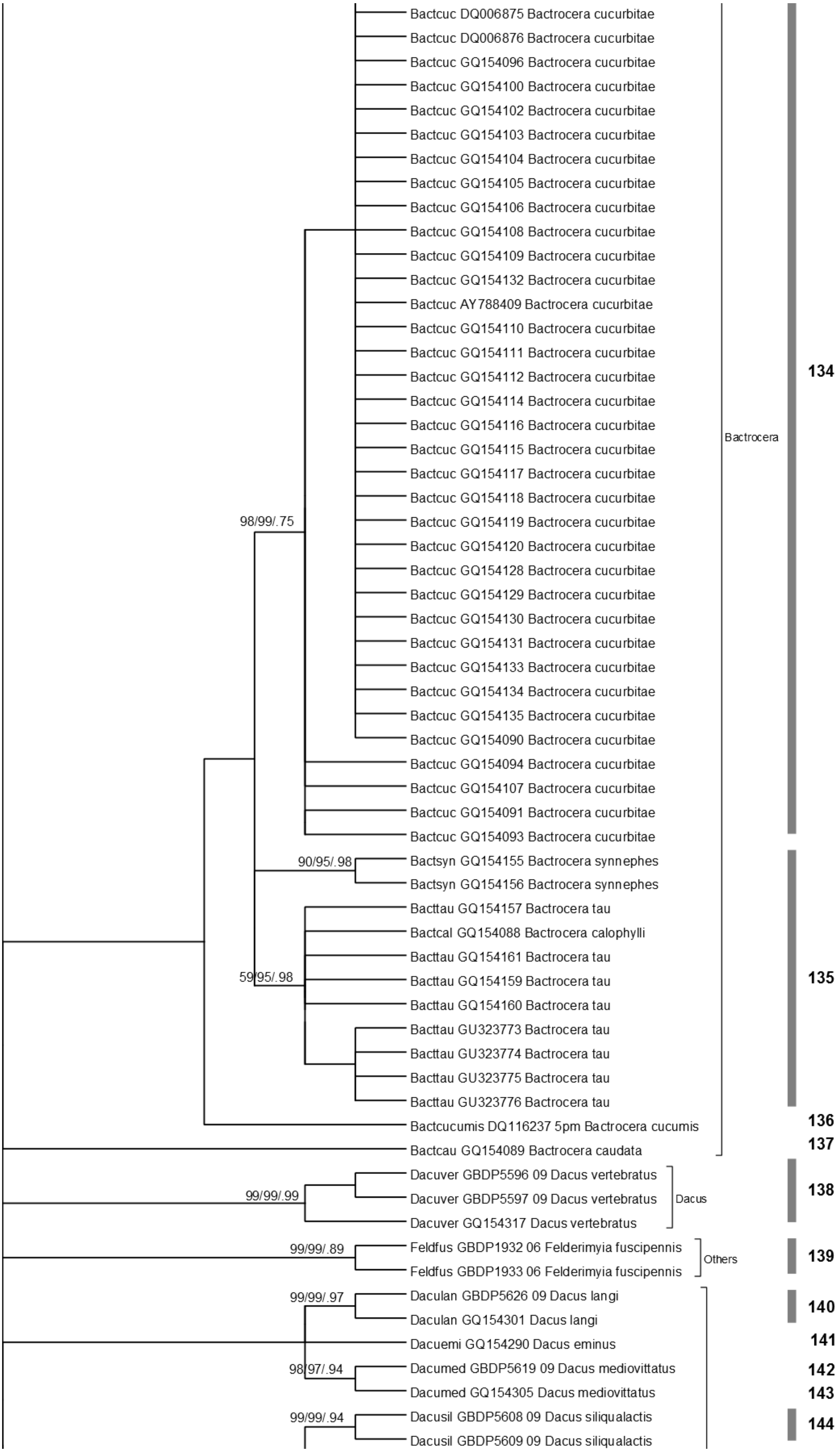
Bactcuc DQ006875 Bactrocera cucurbitae
Bactcuc DQ006876 Bactrocera cucurbitae
Bactcuc GQ154096 Bactrocera cucurbitae
Bactcuc GQ154100 Bactrocera cucurbitae
Bactcuc GQ154102 Bactrocera cucurbitae
Bactcuc GQ154103 Bactrocera cucurbitae
Bactcuc GQ154104 Bactrocera cucurbitae
Bactcuc GQ154105 Bactrocera cucurbitae
Bactcuc GQ154106 Bactrocera cucurbitae
Bactcuc GQ154108 Bactrocera cucurbitae
Bactcuc GQ154109 Bactrocera cucurbitae
Bactcuc GQ154132 Bactrocera cucurbitae
Bactcuc AY788409 Bactrocera cucurbitae
Bactcuc GQ154110 Bactrocera cucurbitae
Bactcuc GQ154111 Bactrocera cucurbitae
Bactcuc GQ154112 Bactrocera cucurbitae
Bactcuc GQ154114 Bactrocera cucurbitae
Bactcuc GQ154116 Bactrocera cucurbitae
Bactcuc GQ154115 Bactrocera cucurbitae
Bactcuc GQ154117 Bactrocera cucurbitae
Bactcuc GQ154118 Bactrocera cucurbitae
Bactcuc GQ154119 Bactrocera cucurbitae
Bactcuc GQ154120 Bactrocera cucurbitae
Bactcuc GQ154128 Bactrocera cucurbitae
Bactcuc GQ154129 Bactrocera cucurbitae
Bactcuc GQ154130 Bactrocera cucurbitae
Bactcuc GQ154131 Bactrocera cucurbitae
Bactcuc GQ154133 Bactrocera cucurbitae
Bactcuc GQ154134 Bactrocera cucurbitae
Bactcuc GQ154135 Bactrocera cucurbitae
Bactcuc GQ154090 Bactrocera cucurbitae
Bactcuc GQ154094 Bactrocera cucurbitae
Bactcuc GQ154107 Bactrocera cucurbitae
Bactcuc GQ154091 Bactrocera cucurbitae
Bactcuc GQ154093 Bactrocera cucurbitae
98/99/.75
Bactrocera
134
90/95/.98
Bactsyn GQ154155 Bactrocera synnephes
Bactsyn GQ154156 Bactrocera synnephes
Bacttau GQ154157 Bactrocera tau
Bactcal GQ154088 Bactrocera calophylli
Bacttau GQ154161 Bactrocera tau
59/95/.98
Bacttau GQ154159 Bactrocera tau
Bacttau GQ154160 Bactrocera tau
Bacttau GU323773 Bactrocera tau
Bacttau GU323774 Bactrocera tau
Bacttau GU323775 Bactrocera tau
Bacttau GU323776 Bactrocera tau
135
Bactcucumis DQ116237 5pm Bactrocera cucumis
136
Bactcau GQ154089 Bactrocera caudata
137
99/99/.99
Dacuver GBDP5596 09 Dacus vertebratus
Dacuver GBDP5597 09 Dacus vertebratus
Dacuver GQ154317 Dacus vertebratus
Dacus
138
99/99/.89
Feldfus GBDP1932 06 Felderimyia fuscipennis
Feldfus GBDP1933 06 Felderimyia fuscipennis
Others
139
99/99/.97
Daculan GBDP5626 09 Dacus langi
Daculan GQ154301 Dacus langi
140
Dacuemi GQ154290 Dacus eminus
141
98/97/.94
Dacumed GBDP5619 09 Dacus mediovittatus
142
Dacumed GQ154305 Dacus mediovittatus
143
99/99/.94
Dacusil GBDP5608 09 Dacus siliqualactis
Dacusil GBDP5609 09 Dacus siliqualactis
144

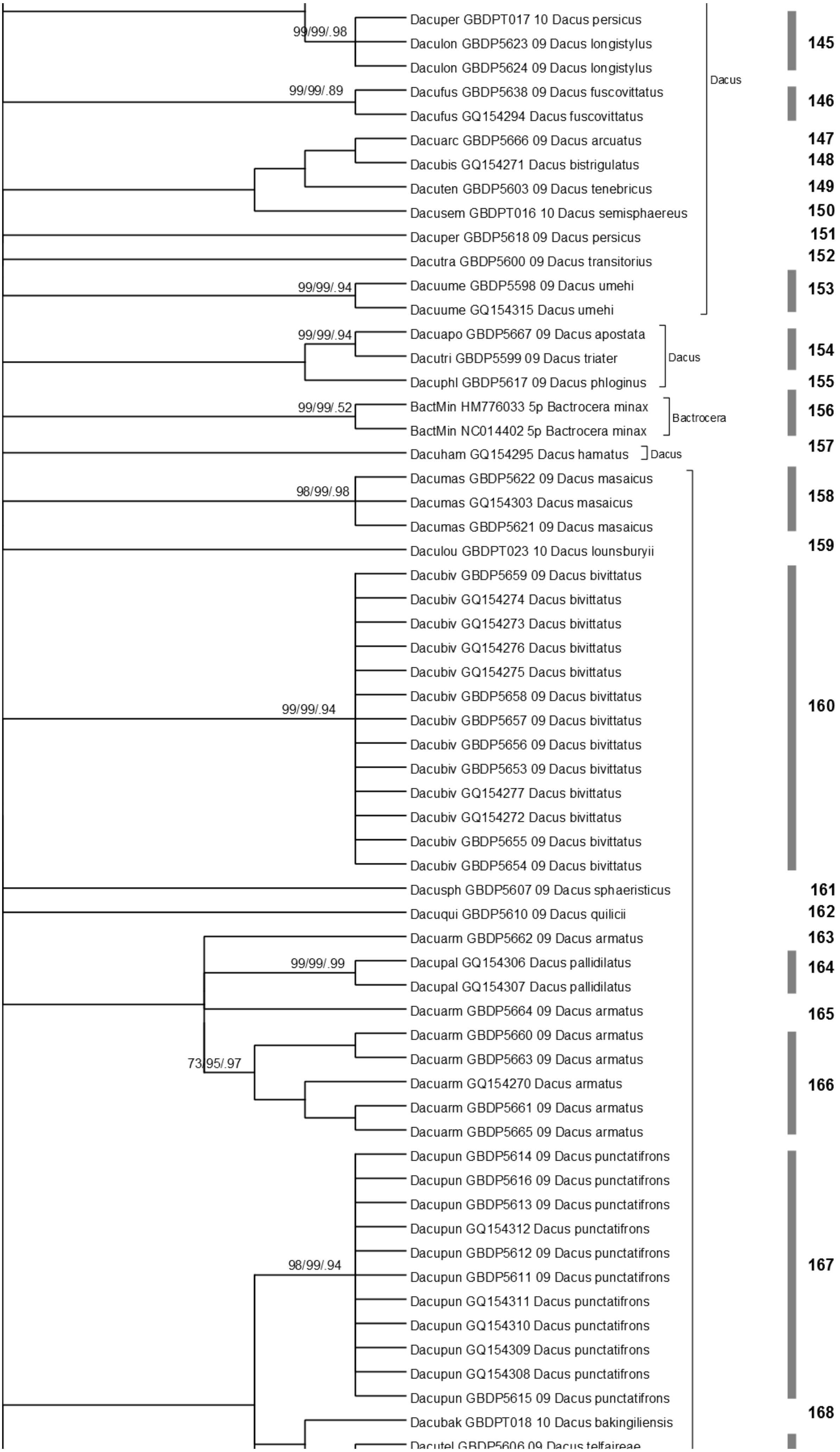
99/99/.98
Dacuper GBDPT017 10 Dacus persicus
Daculon GBDP5623 09 Dacus longistylus
Daculon GBDP5624 09 Dacus longistylus
145
99/99/.89
Dacufus GBDP5638 09 Dacus fuscovittatus
Dacufus GQ154294 Dacus fuscovittatus
146
Dacuarc GBDP5666 09 Dacus arcuatus
147
Dacubis GQ154271 Dacus bistrigulatus
148
Dacuten GBDP5603 09 Dacus tenebricus
149
Dacusem GBDPT016 10 Dacus semisphaereus
150
Dacuper GBDP5618 09 Dacus persicus
151
Dacutra GBDP5600 09 Dacus transitorius
152
99/99/.94
Dacuume GBDP5598 09 Dacus umehi
Dacuume GQ154315 Dacus umehi
153
Dacus
99/99/.94
Dacuapo GBDP5667 09 Dacus apostata
Dacutri GBDP5599 09 Dacus triater
154
Dacuphl GBDP5617 09 Dacus phloginus
155
Dacus
99/99/.52
BactMin HM776033 5p Bactrocera minax
BactMin NC014402 5p Bactrocera minax
Bactrocera
156
Dacuham GQ154295 Dacus hamatus
Dacus
157
98/99/.98
Dacumas GBDP5622 09 Dacus masaicus
Dacumas GQ154303 Dacus masaicus
Dacumas GBDP5621 09 Dacus masaicus
158
Daculou GBDPT023 10 Dacus lounsburyii
159
99/99/.94
Dacubiv GBDP5659 09 Dacus bivittatus
Dacubiv GQ154274 Dacus bivittatus
Dacubiv GQ154273 Dacus bivittatus
Dacubiv GQ154276 Dacus bivittatus
Dacubiv GQ154275 Dacus bivittatus
Dacubiv GBDP5658 09 Dacus bivittatus
Dacubiv GBDP5657 09 Dacus bivittatus
Dacubiv GBDP5656 09 Dacus bivittatus
Dacubiv GBDP5653 09 Dacus bivittatus
Dacubiv GQ154277 Dacus bivittatus
Dacubiv GQ154272 Dacus bivittatus
Dacubiv GBDP5655 09 Dacus bivittatus
Dacubiv GBDP5654 09 Dacus bivittatus
160
Dacusph GBDP5607 09 Dacus sphaeristicus
161
Dacuqui GBDP5610 09 Dacus quilicii
162
Dacuarm GBDP5662 09 Dacus armatus
163
99/99/.99
Dacupal GQ154306 Dacus pallidilatus
Dacupal GQ154307 Dacus pallidilatus
164
Dacuarm GBDP5664 09 Dacus armatus
165
73/95/.97
Dacuarm GBDP5660 09 Dacus armatus
Dacuarm GBDP5663 09 Dacus armatus
Dacuarm GQ154270 Dacus armatus
Dacuarm GBDP5661 09 Dacus armatus
Dacuarm GBDP5665 09 Dacus armatus
166
98/99/.94
Dacupun GBDP5614 09 Dacus punctatifrons
Dacupun GBDP5616 09 Dacus punctatifrons
Dacupun GBDP5613 09 Dacus punctatifrons
Dacupun GQ154312 Dacus punctatifrons
Dacupun GBDP5612 09 Dacus punctatifrons
Dacupun GBDP5611 09 Dacus punctatifrons
Dacupun GQ154311 Dacus punctatifrons
Dacupun GQ154310 Dacus punctatifrons
Dacupun GQ154309 Dacus punctatifrons
Dacupun GQ154308 Dacus punctatifrons
Dacupun GBDP5615 09 Dacus punctatifrons
167
Dacubak GBDPT018 10 Dacus bakingiliensis
168
Dacutel GBDP5606 09 Dacus telfaireae

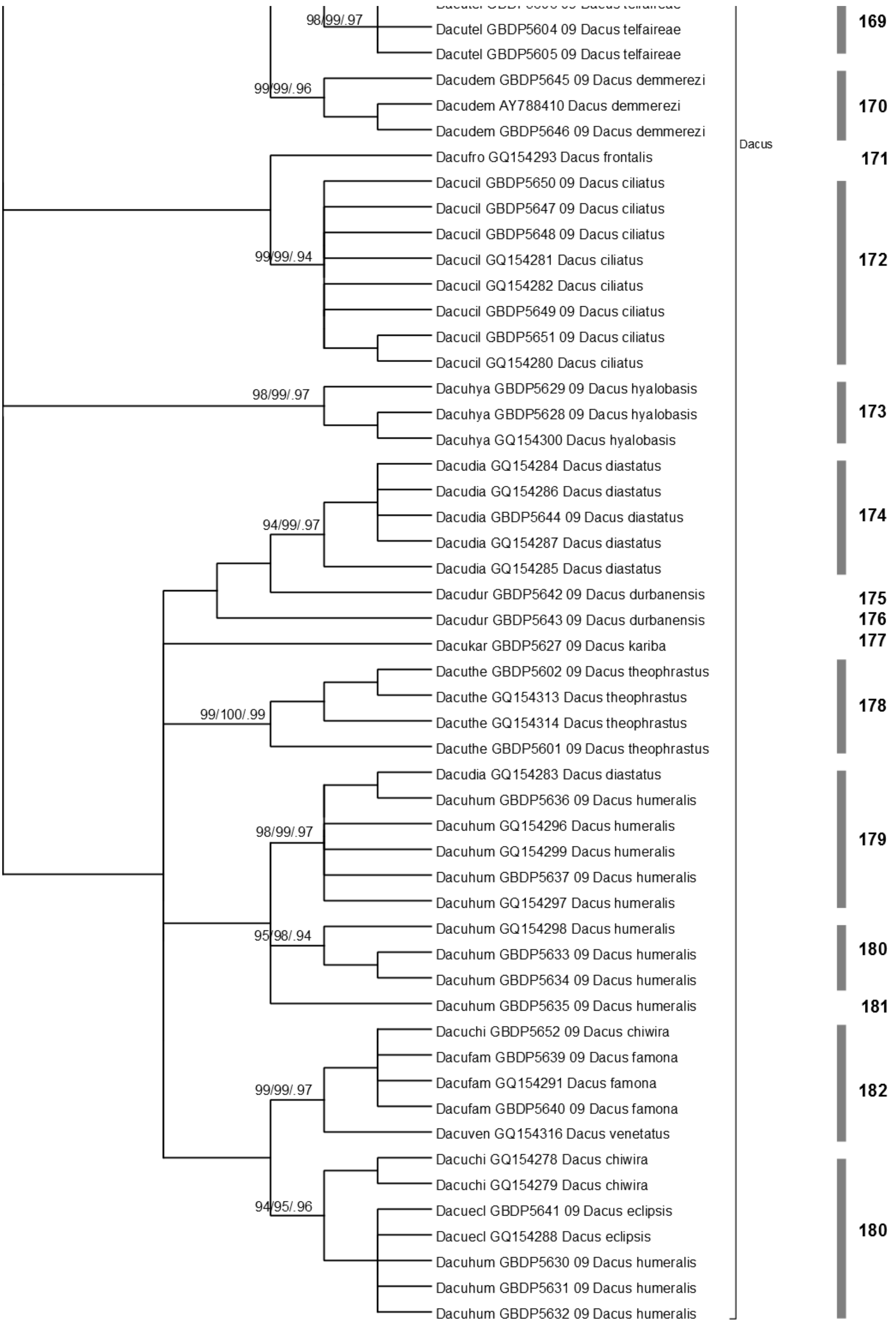

98/99/.97
Dacutel GBDP5604 09 Dacus telfaireae
Dacutel GBDP5605 09 Dacus telfaireae
169
99/99/.96
Dacudem GBDP5645 09 Dacus demmerezi
Dacudem AY788410 Dacus demmerezi
Dacudem GBDP5646 09 Dacus demmerezi
170
Dacus
Dacufro GQ154293 Dacus frontalis
171
99/99/.94
Dacucil GBDP5650 09 Dacus ciliatus
Dacucil GBDP5647 09 Dacus ciliatus
Dacucil GBDP5648 09 Dacus ciliatus
Dacucil GQ154281 Dacus ciliatus
Dacucil GQ154282 Dacus ciliatus
Dacucil GBDP5649 09 Dacus ciliatus
Dacucil GBDP5651 09 Dacus ciliatus
Dacucil GQ154280 Dacus ciliatus
172
98/99/.97
Dacuhya GBDP5629 09 Dacus hyalobasis
Dacuhya GBDP5628 09 Dacus hyalobasis
Dacuhya GQ154300 Dacus hyalobasis
173
94/99/.97
Dacudia GQ154284 Dacus diastatus
Dacudia GQ154286 Dacus diastatus
Dacudia GBDP5644 09 Dacus diastatus
Dacudia GQ154287 Dacus diastatus
Dacudia GQ154285 Dacus diastatus
174
Dacudur GBDP5642 09 Dacus durbanensis
175
Dacudur GBDP5643 09 Dacus durbanensis
176
Dacukar GBDP5627 09 Dacus kariba
177
99/100/.99
Dacuthe GBDP5602 09 Dacus theophrastus
Dacuthe GQ154313 Dacus theophrastus
Dacuthe GQ154314 Dacus theophrastus
Dacuthe GBDP5601 09 Dacus theophrastus
178
98/99/.97
Dacudia GQ154283 Dacus diastatus
Dacuhum GBDP5636 09 Dacus humeralis
Dacuhum GQ154296 Dacus humeralis
Dacuhum GQ154299 Dacus humeralis
Dacuhum GBDP5637 09 Dacus humeralis
Dacuhum GQ154297 Dacus humeralis
179
95/98/.94
Dacuhum GQ154298 Dacus humeralis
Dacuhum GBDP5633 09 Dacus humeralis
Dacuhum GBDP5634 09 Dacus humeralis
180
Dacuhum GBDP5635 09 Dacus humeralis
181
99/99/.97
Dacuchi GBDP5652 09 Dacus chiwira
Dacufam GBDP5639 09 Dacus famona
Dacufam GQ154291 Dacus famona
Dacufam GBDP5640 09 Dacus famona
Dacuven GQ154316 Dacus venetatus
182
94/95/.96
Dacuchi GQ154278 Dacus chiwira
Dacuchi GQ154279 Dacus chiwira
Dacuecl GBDP5641 09 Dacus eclipsis
Dacuecl GQ154288 Dacus eclipsis
Dacuhum GBDP5630 09 Dacus humeralis
Dacuhum GBDP5631 09 Dacus humeralis
Dacuhum GBDP5632 09 Dacus humeralis
180
