# Supplementary material for: Developing diagnostic SNP panels for the identification of true fruit flies (Diptera: Tephritidae) within the limits of COI-based species delimitation
Source: BMC Evol Biol. 2013 May 29;13:106. doi: 10.1186/1471-2148-13-106 (PMC3682933; doi:10.1186/1471-2148-13-106)
Supplement: Additional file 3: Table S3 — SNP panel established with the singleton excluding thresholds (182 OTUs) and with a minimum of three SNPs difference in all pairwise comparisons. The table is split into the five genera Anastrepha, Bactrocera, Ceratitis, Dacus and Rhagoletis and in a sixth group containing all other species, but the analysis was performed over all data, i.e., the three SNPs difference holds for pairwise comparisons of all OTUs. Variable SNPs for each group are in bold. OTU Nr is the same as in the Additional file 2: Figure S1. [file 1471-2148-13-106-S3.doc]

Table 4: SNP panel established with the singleton excluding thresholds (182 OTUs) and with a minimum of three SNPs difference in all pairwise comparisons. The table is split into the five genera *Anastrepha, Bactrocera, Ceratitis, Dacus* and *Rhagoletis* and in a sixth group containing all other species, but the analysis was performed over all data, i.e., the three SNPs difference holds for pairwise comparisons of all OTUs. Variable SNPs for each group are in bold. OTU Nr is the same as in the Supplementary Figure 1.

a)

| Group | OTU | OTU Nr | N | 103 | 108 | **111** | **129** | 131 | **132** | **141** | 142 | 151 | **156** | **174** | **180** | 198 | **222** | 242 | **243** | **246** | **253** | **261** | **312** | **315** | **319** | **321** | **324** | **336** | **354** | **423** | **433** | **448** | **453** | 465 | **469** | **477** | 490 | **492** | 515 | 525 | 533 | **540** | 547 | **562** | **570** | **571** | **577** | **579** | **582** | 585 | **598** | **600** | **606** | **621** | 633 |
| --- | --- | --- | --- | --- | --- | --- | --- | --- | --- | --- | --- | --- | --- | --- | --- | --- | --- | --- | --- | --- | --- | --- | --- | --- | --- | --- | --- | --- | --- | --- | --- | --- | --- | --- | --- | --- | --- | --- | --- | --- | --- | --- | --- | --- | --- | --- | --- | --- | --- | --- | --- | --- | --- | --- | --- |
| Anastrepha | *Anastrepha fraterculus / A. suspensa* | 107 | 3 | A | A | **T** | **A** | C | **C** | **A** | G | A | **T** | **T** | **A** | T | **A** | T | **A** | **T** | **T** | **A** | **T** | **T** | **C** | **T** | **A** | **A** | **C** | **A** | **A** | **T** | **T** | A | **C** | **A** | A | **A** | C | A | G | **T** | G | **T** | **A** | **C** | **C** | **A** | **C** | A | **C** | **A** | **T** | **A** | A |
|  | *Anastrepha ludens* | 106 | 17 | A | A | **T** | **G** | C | **C** | **A** | G | A | **C** | **T** | **G** | T | **A** | T | **A** | **T** | **T** | **A** | **C** | **T** | **C** | **T** | **R** | **A** | **C** | **A** | **A** | **T** | **T** | A | **C** | **A** | A | **A** | C | A | G | **T** | G | **T** | **A** | **C** | **T** | **A** | **T** | A | **C** | **A** | **T** | **A** | A |
|  | *Anastrepha obliqua* | 108 | 6 | A | A | **T** | **A** | C | **C** | **A** | G | A | **T** | **T** | **A** | T | **G** | T | **A** | **T** | **T** | **A** | **C** | **T** | **C** | **T** | **A** | **A** | **C** | **A** | **A** | **T** | **T** | A | **C** | **A** | A | **A** | C | A | G | **T** | G | **T** | **A** | **C** | **T** | **A** | **T** | A | **C** | **A** | **T** | **A** | A |
|  | *Anastrepha pickeli* | 109 | 1 | A | A | **T** | **A** | C | **A** | **A** | G | A | **T** | **T** | **A** | T | **A** | T | **A** | **T** | **C** | **A** | **T** | **C** | **C** | **T** | **A** | **A** | **T** | **A** | **A** | **T** | **A** | A | **T** | **A** | A | **T** | C | A | G | **T** | G | **T** | **A** | **C** | **T** | **A** | **T** | A | **C** | **A** | **T** | **G** | A |
|  | *Anastrepha pickeli* | 110 | 5 | A | A | **T** | **A** | C | **A** | **A** | G | A | **T** | **T** | **A** | T | **A** | T | **A** | **A** | **T** | **A** | **C** | **C** | **C** | **T** | **A** | **A** | **C** | **A** | **A** | **C** | **R** | A | **C** | **A** | A | **A** | C | A | G | **A** | G | **T** | **C** | **C** | **T** | **A** | **T** | A | **C** | **A** | **T** | **A** | A |
|  | *Anastrepha serpentina* | 111 | 6 | A | A | **A** | **A** | C | **C** | **A** | G | A | **T** | **T** | **A** | T | **A** | T | **T** | **T** | **T** | **A** | **Y** | **T** | **C** | **T** | **T** | **A** | **T** | **A** | **A** | **C** | **C** | A | **C** | **A** | A | **A** | C | A | G | **A** | G | **Y** | **T** | **T** | **T** | **A** | **T** | A | **T** | **A** | **T** | **A** | A |
|  | *Anastrepha spp.* | 114 | 3 | A | A | **T** | **A** | C | **C** | **T** | G | A | **C** | **T** | **A** | T | **A** | T | **T** | **T** | **C** | **T** | **C** | **T** | **T** | **A** | **T** | **T** | **C** | **T** | **A** | **T** | **A** | A | **C** | **T** | A | **A** | C | A | G | **A** | G | **T** | **A** | **T** | **C** | **A** | **T** | A | **C** | **T** | **A** | **A** | A |
|  | *Anastrepha striata* | 112 | 1 | A | A | **T** | **A** | C | **A** | **A** | G | A | **C** | **T** | **A** | T | **T** | T | **A** | **C** | **T** | **A** | **T** | **T** | **C** | **T** | **A** | **A** | **C** | **A** | **T** | **C** | **C** | A | **C** | **G** | A | **G** | C | A | G | **A** | G | **T** | **A** | **C** | **T** | **A** | **T** | A | **C** | **A** | **T** | **G** | A |
|  | *Anastrepha striata* | 113 | 9 | A | A | **T** | **A** | C | **C** | **A** | G | A | **C** | **W** | **A** | T | **T** | T | **A** | **C** | **T** | **A** | **T** | **T** | **C** | **T** | **A** | **A** | **C** | **A** | **A** | **C** | **T** | A | **C** | **G** | A | **A** | C | A | G | **A** | G | **T** | **A** | **C** | **T** | **A** | **T** | A | **C** | **A** | **T** | **G** | A |
|  | *Toxotrypana curvicauda* | 115 | 6 | A | A | **T** | **G** | C | **T** | **A** | G | A | **C** | **A** | **T** | T | **A** | T | **A** | **T** | **T** | **T** | **T** | **T** | **T** | **A** | **C** | **T** | **C** | **T** | **A** | **C** | **A** | A | **T** | **T** | A | **T** | C | A | G | **A** | G | **T** | **T** | **T** | **C** | **T** | **A** | A | **C** | **T** | **A** | **A** | A |

b)

| Group | OTU | OTU Nr | N | **103** | **108** | **111** | **129** | 131 | **132** | **141** | 142 | 151 | **156** | **174** | **180** | **198** | **222** | 242 | **243** | **246** | **253** | **261** | **312** | **315** | 319 | **321** | **324** | **336** | **354** | **423** | 433 | **448** | **453** | **465** | **469** | **477** | 490 | **492** | 515 | **525** | 533 | **540** | 547 | **562** | **570** | **571** | **577** | **579** | **582** | **585** | **598** | 600 | **606** | **621** | 633 |
| --- | --- | --- | --- | --- | --- | --- | --- | --- | --- | --- | --- | --- | --- | --- | --- | --- | --- | --- | --- | --- | --- | --- | --- | --- | --- | --- | --- | --- | --- | --- | --- | --- | --- | --- | --- | --- | --- | --- | --- | --- | --- | --- | --- | --- | --- | --- | --- | --- | --- | --- | --- | --- | --- | --- | --- |
| Bactrocera | *Bactrocera albistrigata* | 125 | 1 | **G** | **A** | **T** | **A** | C | **A** | **A** | G | A | **T** | **C** | **T** | **T** | **T** | T | **T** | **T** | **T** | **A** | **T** | **T** | C | **T** | **A** | **A** | **C** | **T** | A | **C** | **T** | **A** | **T** | **A** | A | **A** | C | **A** | G | **T** | G | **C** | **T** | **C** | **C** | **T** | **A** | **A** | **T** | A | **A** | **A** | A |
|  | *Bactrocera breviaculeus* | 122 | 1 | **G** | **A** | **T** | **A** | C | **A** | **A** | G | A | **T** | **C** | **T** | **T** | **T** | T | **T** | **T** | **C** | **A** | **C** | **T** | C | **T** | **A** | **A** | **C** | **T** | A | **T** | **C** | **A** | **T** | **A** | A | **G** | C | **A** | G | **T** | G | **T** | **C** | **C** | **C** | **T** | **A** | **A** | **T** | A | **A** | **A** | A |
|  | *Bactrocera caudata* | 137 | 1 | **G** | **A** | **A** | **A** | C | **C** | **A** | G | A | **T** | **T** | **A** | **T** | **G** | T | **A** | **T** | **C** | **C** | **C** | **T** | C | **T** | **A** | **G** | **T** | **T** | A | **T** | **T** | **T** | **T** | **C** | A | **T** | C | **T** | G | **G** | G | **T** | **C** | **C** | **C** | **T** | **A** | **T** | **T** | A | **A** | **A** | A |
|  | *Bactrocera correcta* | 127 | 2 | **G** | **T** | **C** | **A** | C | **A** | **A** | G | A | **T** | **A** | **T** | **T** | **A** | T | **C** | **C** | **C** | **T** | **C** | **T** | C | **T** | **A** | **A** | **T** | **T** | A | **T** | **T** | **A** | **T** | **A** | A | **A** | C | **A** | G | **T** | G | **T** | **T** | **T** | **C** | **T** | **A** | **A** | **T** | A | **A** | **A** | A |
|  | *Bactrocera cucumis* | 136 | 1 | **G** | **T** | **A** | **A** | C | **T** | **G** | G | A | **T** | **T** | **A** | **T** | **A** | T | **A** | **C** | **T** | **C** | **A** | **T** | C | **T** | **A** | **A** | **C** | **T** | A | **T** | **T** | **A** | **T** | **C** | A | **A** | C | **A** | G | **T** | G | **C** | **C** | **C** | **T** | **A** | **T** | **A** | **C** | A | **A** | **T** | A |
|  | *Bactrocera cucurbitae* | 134 | 47 | **G** | **G** | **A** | **A** | C | **T** | **A** | G | A | **T** | **T** | **A** | **C** | **A** | T | **A** | **C** | **C** | **S** | **T** | **C** | C | **T** | **A** | **G** | **C** | **T** | A | **T** | **T** | **A** | **T** | **C** | A | **T** | C | **A** | G | **T** | G | **T** | **T** | **C** | **T** | **A** | **T** | **A** | **T** | A | **A** | **T** | A |
|  | *Bactrocera dorsalis* | 119 | 1 | **G** | **A** | **T** | **A** | C | **G** | **A** | G | A | **T** | **C** | **C** | **T** | **T** | T | **T** | **C** | **T** | **T** | **T** | **T** | C | **T** | **A** | **G** | **T** | **T** | A | **T** | **C** | **A** | **T** | **A** | A | **A** | C | **T** | G | **T** | G | **T** | **C** | **T** | **C** | **T** | **A** | **A** | **T** | A | **A** | **A** | A |
|  | *Bactrocera kraussi* | 121 | 1 | **G** | **A** | **T** | **A** | C | **A** | **A** | G | A | **T** | **C** | **T** | **T** | **T** | T | **T** | **C** | **T** | **A** | **T** | **T** | C | **T** | **A** | **A** | **C** | **T** | A | **T** | **T** | **A** | **T** | **A** | A | **A** | C | **A** | G | **C** | G | **T** | **T** | **N** | **C** | **T** | **A** | **A** | **T** | A | **A** | **A** | A |
|  | *Bactrocera latifrons* | 124 | 12 | **G** | **A** | **T** | **A** | C | **A** | **A** | G | A | **T** | **C** | **T** | **T** | **T** | T | **T** | **C** | **C** | **A** | **T** | **T** | C | **T** | **A** | **G** | **T** | **C** | A | **T** | **C** | **A** | **T** | **A** | A | **A** | C | **A** | G | **T** | G | **C** | **Y** | **C** | **C** | **C** | **A** | **G** | **T** | A | **A** | **A** | A |
|  | *Bactrocera melas / B. tryoni* | 123 | 2 | **G** | **A** | **T** | **A** | C | **A** | **A** | G | A | **T** | **C** | **T** | **T** | **T** | T | **T** | **T** | **T** | **R** | **Y** | **T** | C | **T** | **A** | **G** | **C** | **T** | A | **T** | **T** | **G** | **T** | **A** | A | **A** | C | **G** | G | **T** | G | **T** | **C** | **C** | **C** | **T** | **A** | **A** | **T** | A | **A** | **A** | A |
|  | *Bactrocera mesomelas* | 129 | 1 | **G** | **A** | **A** | **A** | C | **T** | **A** | G | A | **T** | **C** | **T** | **T** | **A** | T | **T** | **T** | **C** | **A** | **T** | **C** | C | **T** | **C** | **A** | **T** | **T** | A | **T** | **A** | **A** | **T** | **T** | A | **A** | C | **A** | G | **A** | G | **T** | **T** | **C** | **C** | **T** | **A** | **A** | **C** | A | **A** | **T** | A |
|  | *Bactrocera minax* | 156 | 2 | **G** | **A** | **A** | **A** | C | **C** | **A** | G | A | **T** | **C** | **T** | **C** | **A** | T | **A** | **C** | **C** | **C** | **A** | **A** | C | **T** | **C** | **C** | **C** | **C** | A | **C** | **A** | **A** | **C** | **A** | A | **T** | C | **A** | G | **C** | G | **C** | **T** | **C** | **C** | **C** | **A** | **A** | **C** | A | **A** | **T** | A |
|  | *Bactrocera montyanus* | 131 | 1 | **G** | **A** | **A** | **A** | C | **A** | **G** | G | A | **T** | **T** | **T** | **C** | **A** | T | **C** | **T** | **C** | **A** | **C** | **C** | C | **C** | **C** | **T** | **C** | **C** | A | **T** | **A** | **A** | **T** | **T** | A | **A** | C | **A** | G | **T** | G | **C** | **C** | **C** | **C** | **T** | **A** | **A** | **T** | A | **T** | **T** | A |
|  | *Bactrocera munroi* | 132 | 1 | **G** | **A** | **A** | **A** | C | **A** | **A** | G | A | **C** | **T** | **T** | **C** | **A** | T | **C** | **T** | **C** | **A** | **T** | **C** | C | **C** | **T** | **C** | **C** | **C** | A | **T** | **G** | **A** | **T** | **T** | A | **A** | C | **A** | G | **T** | G | **T** | **C** | **C** | **C** | **T** | **A** | **A** | **T** | A | **T** | **T** | A |
|  | *Bactrocera nigrotibialis* | 126 | 2 | **G** | **A** | **T** | **A** | C | **A** | **A** | G | A | **T** | **T** | **T** | **T** | **A** | T | **T** | **C** | **T** | **A** | **T** | **T** | C | **T** | **C** | **G** | **C** | **T** | A | **C** | **T** | **A** | **T** | **G** | A | **A** | C | **A** | G | **T** | G | **T** | **C** | **T** | **C** | **T** | **G** | **A** | **T** | A | **A** | **A** | A |
|  | *Bactrocera nigrotibialis* | 130 | 1 | **G** | **G** | **A** | **A** | C | **C** | **A** | G | A | **C** | **T** | **C** | **C** | **A** | T | **C** | **T** | **C** | **G** | **C** | **C** | C | **T** | **A** | **C** | **C** | **T** | A | **T** | **A** | **C** | **T** | **C** | A | **A** | C | **A** | G | **T** | G | **T** | **C** | **C** | **C** | **T** | **A** | **A** | **T** | A | **A** | **T** | A |
|  | *Bactrocera occipitalis / B. carambolae / B. dorsalis / B. invadens / B. papayae / B. philippinensis* | 118 | 17 | **G** | **A** | **T** | **R** | C | **T** | **T** | G | A | **T** | **T** | **T** | **T** | **T** | T | **T** | **Y** | **T** | **Y** | **T** | **T** | C | **T** | **W** | **A** | **C** | **T** | A | **T** | **R** | **A** | **T** | **A** | A | **A** | C | **C** | G | **Y** | G | **T** | **T** | **Y** | **C** | **T** | **A** | **A** | **T** | A | **R** | **A** | A |
|  | *Bactrocera oleae* | 133 | 10 | **R** | **A** | **A** | **A** | C | **A** | **A** | G | A | **T** | **T** | **T** | **C** | **G** | T | **C** | **T** | **T** | **A** | **C** | **Y** | C | **C** | **T** | **C** | **C** | **C** | A | **T** | **A** | **A** | **T** | **T** | A | **A** | C | **W** | G | **T** | G | **C** | **C** | **C** | **C** | **W** | **W** | **A** | **T** | A | **T** | **T** | A |
|  | *Bactrocera tau / B. synnephes / B. calophylli* | 135 | 11 | **G** | **A** | **A** | **A** | C | **T** | **A** | G | A | **T** | **T** | **A** | **C** | **A** | T | **R** | **T** | **T** | **M** | **Y** | **C** | C | **Y** | **A** | **G** | **C** | **T** | A | **T** | **T** | **A** | **T** | **T** | A | **T** | C | **R** | G | **T** | G | **Y** | **T** | **C** | **T** | **A** | **T** | **A** | **T** | A | **A** | **T** | A |
|  | *Bactrocera umbrosa* | 120 | 3 | **G** | **A** | **T** | **G** | C | **A** | **A** | G | A | **T** | **R** | **T** | **T** | **G** | T | **T** | **T** | **C** | **A** | **T** | **T** | C | **T** | **G** | **A** | **C** | **C** | A | **T** | **T** | **A** | **C** | **C** | A | **T** | C | **A** | G | **T** | G | **T** | **C** | **T** | **C** | **T** | **A** | **A** | **T** | A | **A** | **A** | A |
|  | *Bactrocera zonata* | 128 | 3 | **G** | **T** | **T** | **A** | C | **A** | **A** | G | A | **T** | **A** | **T** | **T** | **A** | T | **T** | **C** | **T** | **A** | **T** | **T** | C | **T** | **G** | **G** | **Y** | **T** | A | **T** | **Y** | **A** | **C** | **A** | A | **A** | C | **A** | G | **T** | G | **T** | **C** | **C** | **C** | **T** | **A** | **A** | **T** | A | **A** | **A** | A |

c)

| Group | OTU | OTU Nr | N | 103 | 108 | 111 | 129 | 131 | 132 | 141 | 142 | 151 | 156 | 174 | 180 | 198 | 222 | 242 | 243 | 246 | 253 | 261 | 312 | 315 | 319 | 321 | 324 | 336 | 354 | 423 | 433 | 448 | 453 | 465 | 469 | 477 | 490 | 492 | 515 | 525 | 533 | 540 | 547 | 562 | 570 | 571 | 577 | 579 | 582 | 585 | 598 | 600 | 606 | 621 | 633 |
| --- | --- | --- | --- | --- | --- | --- | --- | --- | --- | --- | --- | --- | --- | --- | --- | --- | --- | --- | --- | --- | --- | --- | --- | --- | --- | --- | --- | --- | --- | --- | --- | --- | --- | --- | --- | --- | --- | --- | --- | --- | --- | --- | --- | --- | --- | --- | --- | --- | --- | --- | --- | --- | --- | --- | --- |
| Ceratitis | *Ceratitis anonae / C. rosa / C. fasciventris* | 54 | 20 | A | A | T | A | C | A | A | G | A | T | Y | T | T | A | T | A | T | C | T | Y | T | C | T | A | R | N | T | A | T | A | T | T | T | A | N | C | A | G | T | G | C | A | T | T | A | A | T | T | A | A | A | A |
|  | *Ceratitis bremii* | 37 | 5 | A | R | T | A | C | A | Y | G | A | T | C | T | T | A | T | C | T | C | Y | Y | W | C | T | T | A | C | T | A | T | A | A | T | A | A | A | C | T | G | T | G | C | A | T | C | A | D | A | T | A | A | A | A |
|  | *Ceratitis bremii* | 38 | 1 | A | A | T | A | C | A | T | G | A | T | C | T | T | A | C | C | T | C | T | T | T | C | T | A | A | C | C | A | T | A | A | T | A | A | A | C | T | G | T | G | C | A | T | C | T | A | A | T | A | A | A | A |
|  | *Ceratitis capitata / C. caetrata* | 67 | 25 | A | A | T | A | C | A | A | G | A | T | C | T | C | A | T | R | T | C | A | T | T | C | T | A | A | T | W | A | T | T | T | W | T | A | A | C | A | G | T | G | C | A | C | C | A | A | A | W | A | A | A | A |
|  | *Ceratitis catoirii* | 65 | 2 | A | A | T | T | C | A | A | G | A | T | T | T | T | A | T | G | T | C | A | T | T | C | T | A | A | T | A | A | T | A | T | C | T | A | T | C | A | G | A | G | C | T | T | T | A | A | A | T | A | A | A | A |
|  | *Ceratitis contramedia* | 82 | 1 | A | A | T | A | C | C | A | G | A | T | T | T | T | T | T | C | C | T | C | C | T | C | T | A | A | T | T | A | T | T | A | T | C | A | G | C | A | G | C | G | T | T | C | C | A | A | T | T | A | A | A | T |
|  | *Ceratitis contramedia* | 83 | 1 | G | A | T | T | C | C | A | G | A | T | T | T | T | T | T | T | C | T | C | C | T | C | T | T | A | T | C | A | T | C | T | T | C | A | A | C | A | G | C | G | T | T | C | C | A | A | T | T | A | A | A | T |
|  | *Ceratitis copelandi* | 55 | 2 | A | A | T | A | C | A | A | G | A | T | T | T | T | A | T | A | T | C | T | T | T | C | T | A | G | T | T | A | T | A | A | T | T | A | C | C | A | G | T | G | C | A | C | T | A | T | A | C | A | A | A | A |
|  | *Ceratitis cornuta* | 60 | 1 | A | T | T | A | C | A | A | G | A | T | C | T | T | A | T | T | C | C | T | C | T | C | T | A | A | T | T | A | C | A | A | T | T | A | C | C | A | G | T | G | C | A | T | C | T | G | A | T | A | A | T | A |
|  | *Ceratitis cosyra* | 34 | 13 | A | A | T | T | C | A | A | G | A | T | T | T | T | A | T | T | T | C | C | T | T | C | C | A | A | Y | T | A | T | T | A | T | A | A | T | C | A | G | C | G | C | Y | C | T | G | T | T | T | A | A | A | A |
|  | *Ceratitis cosyra* | 31 | 1 | A | A | T | A | C | A | A | G | A | C | C | T | T | G | T | T | T | C | A | C | T | C | T | A | G | T | T | A | T | T | A | T | A | A | C | C | A | G | T | G | C | T | C | T | A | T | C | T | A | A | A | A |
|  | *Ceratitis cosyra* | 33 | 2 | A | A | T | G | C | A | A | G | A | C | C | T | T | A | T | T | T | C | A | T | T | C | T | A | R | C | T | A | T | C | A | T | A | A | T | C | A | G | T | G | T | T | C | T | A | T | T | T | A | A | A | A |
|  | *Ceratitis cristata* | 24 | 1 | A | A | A | G | C | T | T | G | A | T | C | T | T | A | T | A | C | T | C | C | T | C | T | A | G | C | T | A | T | T | A | T | A | A | T | C | A | G | T | G | C | C | C | T | A | A | T | C | A | A | A | A |
|  | *Ceratitis curvata* | 58 | 1 | A | A | T | T | C | A | A | G | A | T | T | T | T | A | T | A | T | T | T | T | T | C | T | A | A | T | T | A | T | A | A | T | T | A | T | C | A | G | A | G | C | A | C | T | A | A | A | T | A | A | T | A |
|  | *Ceratitis cuthbertsoni* | 43 | 1 | A | G | T | A | C | A | T | G | A | C | T | T | T | A | T | A | C | C | T | C | T | C | T | A | A | C | T | A | T | T | A | T | A | A | A | C | T | G | T | G | C | A | C | C | T | A | A | T | A | A | T | A |
|  | *Ceratitis discussa* | 32 | 1 | A | A | T | G | C | A | A | G | A | T | C | T | T | G | T | T | T | C | A | T | T | C | T | A | G | T | T | A | T | T | A | T | A | A | T | C | A | G | C | G | C | T | C | T | A | T | C | T | A | A | A | A |
|  | *Ceratitis ditissima* | 40 | 2 | A | A | T | A | C | A | T | G | A | T | A | T | T | A | T | G | T | C | A | T | T | C | C | A | C | T | T | A | T | T | A | T | A | A | A | C | T | G | T | G | C | A | C | T | G | A | A | T | A | R | A | A |
|  | *Ceratitis divaricata* | 61 | 1 | A | A | T | C | C | T | A | G | A | T | T | T | C | A | T | A | C | C | C | C | C | C | T | A | A | T | T | A | T | A | A | T | T | A | T | C | A | G | T | G | C | C | T | C | T | A | G | T | A | A | T | A |
|  | *Ceratitis edwardsi* | 41 | 1 | A | A | T | A | C | A | T | G | A | C | T | T | T | G | T | A | A | C | A | T | T | C | T | A | A | C | T | A | T | T | A | T | A | A | A | C | T | G | T | G | C | A | C | C | T | A | A | T | A | A | A | A |
|  | *Ceratitis flexuosa / C. colae* | 57 | 4 | A | A | T | A | C | C | R | G | A | T | T | Y | T | R | T | R | T | Y | T | T | T | C | T | A | A | T | T | A | T | A | A | T | C | A | C | C | A | G | Y | G | C | A | T | T | R | A | R | C | R | A | A | A |
|  | *Ceratitis hamata* | 39 | 1 | A | A | T | A | C | A | T | G | A | C | T | T | T | A | T | G | T | C | A | T | T | C | T | A | A | C | T | A | T | T | A | T | A | A | A | C | A | G | T | G | T | A | T | T | G | A | A | T | A | A | A | A |
|  | *Ceratitis lentigera* | 45 | 1 | A | A | T | A | C | A | A | G | A | T | A | T | T | A | T | A | C | C | A | T | C | C | T | A | A | T | T | A | T | T | A | T | T | A | T | C | A | G | A | G | T | A | T | C | A | A | G | T | A | T | A | A |
|  | *Ceratitis lobata* | 59 | 1 | A | A | T | T | C | A | A | G | A | T | T | T | T | A | T | T | T | T | C | T | T | C | T | A | A | T | T | A | T | A | A | T | T | A | A | C | A | G | A | G | C | A | C | T | A | G | A | T | A | A | T | A |
|  | *Ceratitis malgassa* | 66 | 2 | A | A | C | T | C | A | A | G | A | T | T | C | T | A | T | C | T | C | A | Y | T | C | T | A | A | T | A | A | T | A | T | T | T | A | A | C | A | G | A | G | C | A | T | T | A | A | A | T | A | A | A | A |
|  | *Ceratitis marriotti* | 28 | 1 | A | A | T | T | C | A | T | A | A | T | A | T | T | A | T | A | C | C | A | T | T | C | T | A | G | T | T | A | T | A | T | T | A | A | T | C | A | G | T | G | C | A | T | T | A | A | T | T | A | A | A | A |
|  | *Ceratitis millicentae* | 44 | 5 | A | A | T | R | C | A | T | G | A | T | T | T | T | A | T | R | A | C | A | T | T | C | T | A | A | T | T | A | T | C | A | T | A | A | R | C | T | G | C | G | C | R | Y | C | T | T | A | T | A | A | A | A |
|  | *Ceratitis oraria* | 36 | 1 | A | A | G | A | C | A | T | G | A | C | C | T | T | A | T | T | T | C | C | T | C | C | T | A | G | C | T | A | T | C | A | T | C | A | T | C | A | G | T | G | C | T | T | T | G | T | T | T | A | A | A | A |
|  | *Ceratitis pedestris* | 63 | 2 | A | A | T | T | C | T | A | G | A | T | T | T | C | A | T | A | T | C | T | C | T | C | T | A | G | C | T | A | C | A | A | T | C | A | T | C | A | G | T | G | C | A | T | T | A | A | G | T | A | A | T | R |
|  | *Ceratitis perseus* | 62 | 1 | A | A | C | T | C | A | A | A | A | T | T | T | T | A | T | A | T | C | T | T | T | C | T | A | G | T | T | A | T | A | A | T | C | A | C | C | A | G | T | G | C | A | T | T | A | T | A | C | A | A | T | A |
|  | *Ceratitis podocarpi* | 64 | 3 | A | A | T | T | C | A | A | G | A | T | T | C | T | A | T | A | T | C | T | T | T | C | T | A | A | C | T | A | T | A | A | T | T | A | C | C | A | G | A | G | C | A | T | T | A | A | A | T | A | A | A | A |
|  | *Ceratitis punctata* | 42 | 1 | A | A | T | A | C | A | T | G | A | C | C | T | T | A | T | A | A | C | A | A | C | C | T | A | A | C | T | A | T | T | A | T | A | A | A | C | A | G | T | G | C | G | C | C | T | A | G | T | A | A | A | A |
|  | *Ceratitis quinaria / C. silvestrii* | 30 | 10 | A | A | T | A | C | A | R | G | A | T | C | T | T | A | T | T | T | C | A | T | T | C | T | A | A | C | T | A | T | T | A | T | A | A | T | C | A | G | T | G | C | C | C | T | A | T | T | T | A | A | A | A |
|  | *Ceratitis rubivora* | 56 | 2 | A | A | T | A | C | A | A | G | A | T | T | T | T | A | T | A | T | C | T | T | T | C | T | A | A | C | T | A | T | A | A | T | T | A | T | C | A | G | T | G | C | A | C | T | A | A | A | T | A | G | A | A |
|  | *Ceratitis striatella* | 35 | 2 | A | A | T | A | C | A | A | G | A | T | T | T | T | A | T | T | T | C | A | T | T | C | T | A | S | T | T | A | T | T | T | T | A | A | A | C | A | G | T | G | C | T | C | T | A | T | T | T | A | A | A | A |
|  | *Ceratitis venusta* | 29 | 1 | A | A | T | T | C | A | T | A | A | C | A | T | T | A | T | A | C | C | A | T | T | C | T | A | A | T | T | A | T | A | T | T | A | A | C | C | A | G | T | G | A | A | C | T | A | A | T | T | A | A | A | A |

d)

| Group | OTU | OTU Nr | N | **103** | **108** | **111** | **129** | 131 | **132** | **141** | 142 | 151 | **156** | **174** | **180** | **198** | **222** | **242** | **243** | **246** | **253** | **261** | **312** | **315** | **319** | **321** | **324** | **336** | **354** | **423** | 433 | **448** | **453** | **465** | **469** | **477** | 490 | **492** | 515 | **525** | 533 | **540** | 547 | **562** | **570** | **571** | **577** | **579** | **582** | **585** | **598** | **600** | **606** | **621** | 633 |
| --- | --- | --- | --- | --- | --- | --- | --- | --- | --- | --- | --- | --- | --- | --- | --- | --- | --- | --- | --- | --- | --- | --- | --- | --- | --- | --- | --- | --- | --- | --- | --- | --- | --- | --- | --- | --- | --- | --- | --- | --- | --- | --- | --- | --- | --- | --- | --- | --- | --- | --- | --- | --- | --- | --- | --- |
| Dacus | *Dacus apostata / D. triater* | 154 | 2 | **A** | **T** | **T** | **A** | C | **T** | **A** | G | A | **T** | **C** | **A** | **C** | **A** | **T** | **A** | **T** | **C** | **C** | **C** | **A** | **C** | **T** | **C** | **C** | **C** | **T** | A | **T** | **A** | **T** | **T** | **C** | A | **T** | C | **A** | G | **T** | G | **T** | **T** | **T** | **T** | **G** | **C** | **T** | **T** | **A** | **A** | **A** | A |
|  | *Dacus arcuatus* | 147 | 1 | **G** | **A** | **C** | **A** | C | **A** | **A** | G | A | **T** | **T** | **A** | **C** | **A** | **T** | **A** | **A** | **C** | **C** | **C** | **T** | **C** | **A** | **C** | **C** | **C** | **T** | A | **C** | **T** | **C** | **T** | **T** | A | **T** | C | **A** | G | **T** | G | **T** | **T** | **C** | **C** | **A** | **A** | **G** | **T** | **A** | **A** | **A** | A |
|  | *Dacus armatus* | 166 | 5 | **G** | **A** | **T** | **A** | C | **T** | **A** | G | A | **C** | **C** | **A** | **T** | **A** | **T** | **A** | **C** | **C** | **C** | **A** | **A** | **C** | **C** | **C** | **C** | **C** | **T** | A | **T** | **R** | **T** | **T** | **C** | A | **T** | C | **A** | G | **T** | G | **T** | **Y** | **T** | **C** | **A** | **A** | **T** | **T** | **A** | **A** | **A** | A |
|  | *Dacus armatus* | 163 | 1 | **A** | **A** | **T** | **A** | C | **T** | **C** | G | A | **C** | **T** | **A** | **T** | **A** | **T** | **A** | **C** | **C** | **T** | **C** | **A** | **C** | **A** | **C** | **C** | **C** | **C** | A | **T** | **A** | **T** | **T** | **C** | A | **C** | C | **A** | G | **C** | G | **T** | **C** | **T** | **C** | **A** | **A** | **T** | **T** | **A** | **A** | **G** | A |
|  | *Dacus armatus* | 165 | 1 | **G** | **A** | **T** | **A** | C | **T** | **A** | G | A | **C** | **C** | **A** | **T** | **A** | **T** | **A** | **C** | **C** | **C** | **C** | **A** | **C** | **A** | **C** | **C** | **C** | **T** | A | **T** | **A** | **T** | **C** | **T** | A | **T** | C | **A** | G | **T** | G | **T** | **C** | **T** | **C** | **A** | **A** | **T** | **C** | **A** | **A** | **A** | A |
|  | *Dacus bakingiliensis* | 168 | 1 | **G** | **A** | **A** | **A** | C | **C** | **T** | G | A | **T** | **T** | **A** | **T** | **A** | **T** | **G** | **C** | **C** | **C** | **C** | **A** | **C** | **A** | **C** | **C** | **C** | **A** | A | **T** | **T** | **A** | **T** | **A** | A | **T** | C | **T** | G | **T** | G | **T** | **C** | **C** | **T** | **A** | **A** | **T** | **C** | **A** | **T** | **A** | A |
|  | *Dacus bistrigulatus* | 148 | 1 | **G** | **A** | **C** | **A** | C | **A** | **A** | G | A | **T** | **T** | **A** | **C** | **A** | **T** | **C** | **T** | **C** | **C** | **C** | **T** | **C** | **A** | **A** | **C** | **C** | **T** | A | **C** | **C** | **G** | **T** | **T** | A | **T** | C | **G** | G | **T** | G | **T** | **A** | **T** | **T** | **A** | **G** | **G** | **C** | **A** | **A** | **A** | A |
|  | *Dacus bivittatus* | 160 | 13 | **G** | **A** | **T** | **A** | C | **T** | **A** | G | A | **T** | **C** | **A** | **T** | **A** | **T** | **R** | **C** | **C** | **C** | **C** | **A** | **C** | **A** | **C** | **C** | **C** | **T** | A | **T** | **R** | **A** | **T** | **T** | A | **C** | C | **A** | G | **T** | G | **C** | **T** | **T** | **T** | **A** | **A** | **T** | **T** | **A** | **A** | **A** | A |
|  | *Dacus ciliatus* | 172 | 8 | **G** | **T** | **T** | **A** | C | **T** | **A** | G | A | **T** | **Y** | **A** | **T** | **A** | **T** | **A** | **A** | **T** | **Y** | **T** | **A** | **C** | **T** | **C** | **C** | **C** | **A** | A | **T** | **A** | **A** | **T** | **T** | A | **A** | C | **C** | G | **Y** | G | **T** | **A** | **T** | **T** | **A** | **T** | **T** | **C** | **A** | **A** | **A** | A |
|  | *Dacus demmerezi* | 170 | 3 | **G** | **A** | **A** | **A** | C | **C** | **A** | G | A | **T** | **T** | **A** | **T** | **A** | **T** | **A** | **C** | **C** | **C** | **C** | **A** | **C** | **A** | **C** | **C** | **T** | **A** | A | **T** | **G** | **A** | **T** | **T** | A | **T** | C | **T** | G | **T** | G | **T** | **T** | **C** | **C** | **A** | **A** | **T** | **T** | **A** | **A** | **A** | A |
|  | *Dacus diastatus* | 174 | 5 | **G** | **R** | **T** | **A** | C | **C** | **T** | G | A | **T** | **C** | **C** | **T** | **A** | **T** | **R** | **C** | **C** | **Y** | **T** | **A** | **C** | **Y** | **C** | **C** | **T** | **A** | A | **T** | **A** | **C** | **T** | **T** | A | **C** | C | **A** | G | **T** | G | **T** | **A** | **C** | **T** | **A** | **G** | **T** | **C** | **G** | **A** | **T** | A |
|  | *Dacus durbanensis* | 175 | 1 | **G** | **A** | **T** | **A** | C | **C** | **T** | G | A | **T** | **T** | **C** | **T** | **A** | **T** | **G** | **C** | **C** | **T** | **A** | **A** | **C** | **C** | **C** | **C** | **T** | **A** | A | **T** | **A** | **C** | **T** | **T** | A | **C** | C | **A** | G | **T** | G | **T** | **A** | **C** | **T** | **A** | **G** | **T** | **T** | **A** | **A** | **T** | A |
|  | *Dacus durbanensis* | 176 | 1 | **G** | **A** | **T** | **A** | C | **C** | **C** | G | A | **T** | **T** | **A** | **T** | **A** | **T** | **A** | **T** | **C** | **C** | **C** | **A** | **C** | **C** | **C** | **C** | **T** | **A** | A | **T** | **C** | **T** | **T** | **T** | A | **C** | C | **A** | G | **T** | G | **C** | **A** | **T** | **T** | **A** | **G** | **C** | **C** | **G** | **A** | **T** | A |
|  | *Dacus eminus* | 141 | 1 | **G** | **T** | **T** | **A** | C | **C** | **A** | G | A | **T** | **C** | **A** | **T** | **A** | **T** | **A** | **T** | **C** | **C** | **T** | **T** | **C** | **T** | **G** | **A** | **T** | **T** | A | **T** | **A** | **A** | **C** | **A** | A | **T** | C | **C** | G | **T** | G | **C** | **T** | **C** | **T** | **A** | **A** | **T** | **T** | **A** | **A** | **A** | A |
|  | *Dacus famona / D. chiwira / D. venetatus* | 182 | 5 | **G** | **Y** | **T** | **A** | C | **C** | **T** | G | A | **T** | **C** | **A** | **T** | **A** | **T** | **T** | **R** | **C** | **A** | **C** | **A** | **C** | **A** | **C** | **C** | **T** | **A** | A | **T** | **A** | **A** | **T** | **C** | A | **T** | C | **T** | G | **C** | G | **C** | **A** | **C** | **T** | **A** | **A** | **A** | **T** | **G** | **A** | **T** | A |
|  | *Dacus frontalis* | 171 | 1 | **A** | **T** | **T** | **A** | C | **C** | **A** | G | A | **T** | **T** | **A** | **C** | **G** | **T** | **A** | **T** | **C** | **C** | **T** | **A** | **C** | **T** | **C** | **C** | **C** | **A** | A | **T** | **A** | **A** | **T** | **C** | A | **A** | C | **T** | G | **T** | G | **C** | **C** | **C** | **C** | **A** | **A** | **A** | **C** | **A** | **G** | **A** | A |
|  | *Dacus fuscovittatus* | 146 | 2 | **G** | **A** | **C** | **T** | C | **T** | **A** | G | A | **C** | **G** | **A** | **T** | **A** | **T** | **T** | **A** | **T** | **C** | **C** | **T** | **C** | **T** | **G** | **A** | **T** | **C** | A | **T** | **T** | **A** | **T** | **C** | A | **C** | C | **C** | G | **T** | G | **C** | **A** | **C** | **C** | **T** | **T** | **T** | **T** | **A** | **A** | **A** | A |
|  | *Dacus hamatus* | 157 | 1 | **A** | **T** | **T** | **A** | C | **C** | **A** | G | A | **C** | **T** | **A** | **T** | **A** | **T** | **A** | **C** | **C** | **C** | **C** | **A** | **C** | **T** | **C** | **A** | **C** | **T** | A | **T** | **G** | **T** | **T** | **A** | A | **T** | C | **A** | G | **C** | G | **C** | **T** | **T** | **C** | **A** | **A** | **T** | **T** | **G** | **A** | **T** | A |
|  | *Dacus humeralis* | 181 | 1 | **G** | **C** | **T** | **G** | C | **C** | **T** | G | A | **T** | **C** | **G** | **T** | **A** | **T** | **A** | **C** | **C** | **C** | **C** | **A** | **C** | **A** | **C** | **C** | **C** | **A** | A | **T** | **G** | **C** | **T** | **T** | A | **T** | C | **A** | G | **T** | G | **C** | **A** | **C** | **T** | **A** | **A** | **T** | **T** | **G** | **A** | **T** | A |
|  | *Dacus humeralis / D. chiwira / D. eclipsis* | 180 | 10 | **G** | **A** | **T** | **R** | C | **A** | **T** | G | A | **Y** | **T** | **A** | **T** | **A** | **T** | **C** | **A** | **C** | **C** | **Y** | **A** | **C** | **A** | **C** | **C** | **T** | **A** | A | **T** | **A** | **C** | **T** | **T** | A | **C** | C | **C** | G | **T** | G | **C** | **A** | **C** | **T** | **A** | **R** | **M** | **T** | **A** | **A** | **T** | A |
|  | *Dacus humeralis / D. diastatus* | 179 | 6 | **R** | **T** | **C** | **A** | C | **C** | **T** | G | A | **T** | **C** | **A** | **T** | **A** | **T** | **A** | **T** | **C** | **C** | **C** | **A** | **C** | **A** | **A** | **C** | **C** | **A** | A | **T** | **A** | **C** | **T** | **T** | A | **T** | C | **A** | G | **A** | G | **T** | **A** | **Y** | **T** | **A** | **A** | **T** | **T** | **A** | **A** | **T** | A |
|  | *Dacus hyalobasis* | 173 | 3 | **G** | **A** | **T** | **A** | C | **C** | **R** | G | A | **T** | **C** | **A** | **C** | **A** | **T** | **A** | **C** | **C** | **C** | **T** | **A** | **C** | **C** | **T** | **C** | **C** | **A** | A | **T** | **A** | **T** | **C** | **T** | A | **A** | C | **A** | G | **T** | G | **C** | **C** | **C** | **C** | **A** | **A** | **T** | **C** | **A** | **A** | **T** | A |
|  | *Dacus kariba* | 177 | 1 | **G** | **A** | **T** | **A** | C | **T** | **A** | G | A | **T** | **T** | **A** | **T** | **A** | **T** | **A** | **C** | **C** | **C** | **C** | **A** | **C** | **C** | **C** | **C** | **C** | **A** | A | **T** | **T** | **T** | **C** | **C** | A | **C** | C | **A** | G | **T** | G | **C** | **T** | **C** | **T** | **A** | **A** | **A** | **T** | **A** | **A** | **T** | A |
|  | *Dacus langi* | 140 | 2 | **G** | **C** | **C** | **G** | C | **T** | **A** | G | A | **T** | **T** | **A** | **T** | **A** | **T** | **G** | **T** | **C** | **C** | **T** | **C** | **C** | **T** | **A** | **G** | **T** | **T** | A | **T** | **A** | **A** | **C** | **A** | A | **C** | C | **T** | G | **T** | G | **C** | **A** | **C** | **T** | **G** | **A** | **A** | **T** | **A** | **A** | **A** | A |
|  | *Dacus longistylus / D. persicus* | 145 | 3 | **G** | **T** | **T** | **A** | C | **C** | **A** | G | A | **T** | **T** | **A** | **C** | **A** | **T** | **T** | **T** | **T** | **A** | **T** | **C** | **C** | **T** | **G** | **G** | **T** | **C** | A | **T** | **A** | **T** | **T** | **C** | A | **T** | C | **T** | G | **T** | G | **C** | **A** | **C** | **T** | **A** | **A** | **C** | **T** | **A** | **R** | **A** | A |
|  | *Dacus lounsburyii* | 159 | 1 | **G** | **A** | **T** | **C** | C | **C** | **A** | G | A | **T** | **T** | **A** | **C** | **A** | **T** | **A** | **C** | **C** | **C** | **A** | **A** | **C** | **C** | **C** | **C** | **T** | **T** | A | **T** | **C** | **T** | **T** | **T** | A | **C** | C | **A** | G | **T** | G | **C** | **C** | **C** | **T** | **G** | **A** | **A** | **T** | **A** | **A** | **T** | A |
|  | *Dacus masaicus* | 158 | 3 | **G** | **A** | **C** | **A** | C | **C** | **T** | G | A | **C** | **T** | **A** | **T** | **A** | **T** | **A** | **C** | **C** | **C** | **T** | **A** | **C** | **T** | **C** | **C** | **T** | **T** | A | **T** | **C** | **T** | **C** | **T** | A | **T** | C | **C** | G | **T** | G | **C** | **T** | **C** | **C** | **A** | **A** | **A** | **C** | **A** | **A** | **A** | A |
|  | *Dacus mediovittatus* | 142 | 1 | **G** | **T** | **T** | **T** | C | **T** | **T** | G | A | **T** | **C** | **A** | **T** | **A** | **T** | **C** | **T** | **C** | **T** | **T** | **A** | **C** | **T** | **A** | **C** | **C** | **T** | A | **C** | **A** | **A** | **C** | **T** | A | **T** | C | **A** | G | **T** | G | **C** | **A** | **T** | **T** | **A** | **T** | **C** | **T** | **A** | **A** | **A** | A |
|  | *Dacus mediovittatus* | 143 | 1 | **G** | **T** | **T** | **T** | C | **T** | **A** | G | A | **T** | **T** | **A** | **T** | **A** | **T** | **T** | **T** | **C** | **T** | **C** | **C** | **C** | **T** | **A** | **C** | **T** | **T** | A | **C** | **A** | **A** | **C** | **T** | A | **C** | C | **T** | G | **T** | G | **C** | **A** | **T** | **C** | **A** | **C** | **C** | **T** | **A** | **A** | **A** | A |
|  | *Dacus pallidilatus* | 164 | 2 | **G** | **A** | **T** | **A** | C | **T** | **A** | G | A | **C** | **C** | **A** | **T** | **A** | **T** | **A** | **C** | **C** | **C** | **C** | **A** | **C** | **C** | **C** | **C** | **C** | **T** | A | **T** | **A** | **T** | **T** | **A** | A | **T** | C | **A** | G | **T** | G | **T** | **T** | **C** | **C** | **A** | **A** | **T** | **C** | **A** | **A** | **A** | A |
|  | *Dacus persicus* | 151 | 1 | **G** | **T** | **T** | **A** | C | **A** | **A** | G | A | **T** | **C** | **A** | **C** | **A** | **T** | **A** | **T** | **T** | **C** | **T** | **T** | **C** | **T** | **A** | **G** | **C** | **A** | A | **T** | **A** | **A** | **T** | **A** | A | **C** | C | **A** | G | **T** | G | **T** | **A** | **T** | **T** | **A** | **A** | **A** | **T** | **A** | **G** | **A** | A |
|  | *Dacus phloginus* | 155 | 1 | **A** | **T** | **T** | **A** | C | **T** | **A** | G | A | **C** | **C** | **A** | **T** | **A** | **T** | **A** | **C** | **C** | **C** | **T** | **A** | **C** | **T** | **T** | **C** | **C** | **T** | A | **T** | **A** | **T** | **T** | **T** | A | **G** | C | **A** | G | **C** | G | **T** | **C** | **T** | **C** | **A** | **T** | **T** | **T** | **A** | **A** | **A** | A |
|  | *Dacus punctatifrons* | 167 | 11 | **G** | **A** | **A** | **T** | C | **C** | **T** | G | A | **T** | **C** | **A** | **T** | **A** | **T** | **T** | **T** | **C** | **C** | **C** | **A** | **C** | **A** | **C** | **C** | **T** | **A** | A | **T** | **C** | **A** | **T** | **T** | A | **A** | C | **A** | G | **T** | G | **T** | **T** | **C** | **C** | **T** | **A** | **T** | **C** | **A** | **A** | **A** | A |
|  | *Dacus quilicii* | 162 | 1 | **G** | **A** | **T** | **A** | C | **T** | **A** | G | A | **T** | **T** | **A** | **T** | **G** | **T** | **A** | **C** | **C** | **T** | **T** | **A** | **C** | **T** | **C** | **C** | **C** | **T** | A | **T** | **T** | **T** | **T** | **C** | A | **T** | C | **A** | G | **C** | G | **C** | **C** | **C** | **T** | **A** | **T** | **T** | **C** | **G** | **A** | **A** | A |
|  | *Dacus semisphaereus* | 150 | 1 | **G** | **A** | **T** | **A** | C | **A** | **A** | G | A | **T** | **C** | **A** | **C** | **G** | **T** | **A** | **T** | **C** | **C** | **A** | **T** | **C** | **T** | **C** | **C** | **C** | **C** | A | **T** | **A** | **A** | **T** | **A** | A | **T** | C | **A** | G | **T** | G | **T** | **C** | **C** | **T** | **G** | **T** | **A** | **C** | **A** | **A** | **A** | A |
|  | *Dacus siliqualactis* | 144 | 2 | **G** | **T** | **T** | **G** | C | **T** | **A** | G | A | **T** | **C** | **A** | **C** | **A** | **T** | **A** | **T** | **T** | **G** | **C** | **T** | **C** | **T** | **A** | **G** | **T** | **T** | A | **T** | **A** | **T** | **T** | **C** | A | **T** | C | **A** | G | **T** | G | **T** | **A** | **T** | **T** | **A** | **A** | **C** | **T** | **A** | **A** | **A** | A |
|  | *Dacus sphaeristicus* | 161 | 1 | **G** | **A** | **T** | **A** | C | **C** | **T** | G | A | **T** | **T** | **A** | **C** | **A** | **T** | **C** | **C** | **C** | **T** | **A** | **A** | **C** | **C** | **C** | **C** | **C** | **T** | A | **T** | **C** | **T** | **T** | **T** | A | **T** | C | **T** | G | **T** | G | **T** | **T** | **C** | **T** | **A** | **A** | **T** | **T** | **A** | **A** | **A** | A |
|  | *Dacus telfaireae* | 169 | 3 | **G** | **A** | **A** | **A** | C | **C** | **T** | G | A | **C** | **T** | **A** | **T** | **A** | **T** | **A** | **C** | **C** | **C** | **C** | **A** | **T** | **A** | **C** | **C** | **C** | **A** | A | **T** | **T** | **A** | **T** | **A** | A | **T** | C | **T** | G | **T** | G | **T** | **C** | **C** | **T** | **A** | **A** | **T** | **C** | **A** | **T** | **A** | A |
|  | *Dacus tenebricus* | 149 | 1 | **G** | **A** | **C** | **C** | C | **A** | **A** | G | A | **T** | **T** | **A** | **C** | **A** | **T** | **A** | **C** | **C** | **C** | **T** | **T** | **C** | **A** | **C** | **C** | **C** | **T** | A | **C** | **T** | **A** | **T** | **T** | A | **C** | C | **A** | G | **T** | G | **T** | **T** | **T** | **C** | **A** | **A** | **A** | **T** | **A** | **A** | **A** | A |
|  | *Dacus theophrastus* | 178 | 4 | **G** | **A** | **A** | **R** | C | **C** | **C** | G | A | **T** | **T** | **A** | **T** | **T** | **T** | **G** | **G** | **C** | **C** | **C** | **T** | **C** | **A** | **C** | **C** | **C** | **A** | A | **T** | **A** | **A** | **T** | **T** | A | **C** | C | **A** | G | **Y** | G | **T** | **A** | **T** | **C** | **A** | **A** | **T** | **T** | **R** | **W** | **T** | A |
|  | *Dacus transitorius* | 152 | 1 | **G** | **A** | **C** | **A** | C | **A** | **A** | G | A | **T** | **A** | **A** | **C** | **G** | **T** | **T** | **T** | **T** | **C** | **A** | **T** | **C** | **T** | **A** | **C** | **T** | **A** | A | **C** | **A** | **T** | **C** | **C** | A | **C** | C | **A** | G | **T** | G | **T** | **C** | **C** | **T** | **A** | **A** | **T** | **T** | **A** | **A** | **A** | A |
|  | *Dacus umehi* | 153 | 2 | **G** | **T** | **T** | **G** | C | **A** | **A** | G | A | **C** | **T** | **A** | **C** | **A** | **T** | **C** | **T** | **C** | **T** | **C** | **T** | **C** | **T** | **A** | **C** | **C** | **A** | A | **T** | **C** | **A** | **C** | **C** | A | **C** | C | **A** | G | **C** | G | **T** | **C** | **C** | **C** | **A** | **A** | **C** | **C** | **C** | **A** | **A** | A |
|  | *Dacus vertebratus* | 138 | 3 | **G** | **A** | **T** | **A** | C | **A** | **T** | G | A | **T** | **C** | **T** | **C** | **A** | **T** | **A** | **T** | **T** | **A** | **Y** | **C** | **C** | **T** | **G** | **A** | **C** | **A** | A | **C** | **T** | **T** | **T** | **C** | A | **T** | C | **A** | G | **A** | G | **T** | **T** | **T** | **T** | **A** | **T** | **T** | **T** | **A** | **A** | **A** | A |

e)

| Group | OTU | OTU Nr | N | 103 | 108 | **111** | **129** | 131 | **132** | **141** | 142 | 151 | **156** | **174** | **180** | **198** | **222** | 242 | **243** | **246** | **253** | **261** | **312** | 315 | **319** | **321** | **324** | **336** | **354** | **423** | 433 | 448 | **453** | **465** | **469** | **477** | 490 | **492** | **515** | 525 | 533 | **540** | 547 | **562** | **570** | **571** | **577** | **579** | **582** | **585** | **598** | **600** | 606 | 621 | 633 |
| --- | --- | --- | --- | --- | --- | --- | --- | --- | --- | --- | --- | --- | --- | --- | --- | --- | --- | --- | --- | --- | --- | --- | --- | --- | --- | --- | --- | --- | --- | --- | --- | --- | --- | --- | --- | --- | --- | --- | --- | --- | --- | --- | --- | --- | --- | --- | --- | --- | --- | --- | --- | --- | --- | --- | --- |
| Rhagoletis | *Rhagoletis basiola* | 12 | 4 | A | A | **T** | **A** | C | **C** | **T** | G | A | **T** | **T** | **T** | **T** | **A** | T | **T** | **T** | **T** | **A** | **C** | T | **C** | **T** | **A** | **T** | **T** | **T** | A | T | **A** | **T** | **T** | **A** | A | **A** | **C** | A | G | **A** | G | **T** | **A** | **T** | **T** | **A** | **A** | **A** | **T** | **A** | A | T | A |
|  | *Rhagoletis cerasi* | 13 | 13 | A | A | **T** | **T** | C | **T** | **T** | G | A | **C** | **C** | **C** | **T** | **A** | T | **T** | **C** | **C** | **A** | **T** | T | **C** | **T** | **A** | **T** | **K** | **T** | A | T | **A** | **A** | **T** | **A** | A | **T** | **C** | A | G | **A** | G | **T** | **T** | **T** | **T** | **A** | **A** | **A** | **T** | **G** | A | T | A |
|  | *Rhagoletis cingulata / R. turpiniae* | 3 | 27 | A | A | **T** | **T** | C | **T** | **T** | G | A | **T** | **T** | **T** | **T** | **T** | T | **A** | **C** | **T** | **Y** | **A** | T | **C** | **T** | **A** | **A** | **T** | **T** | A | T | **A** | **T** | **T** | **A** | A | **A** | **C** | A | G | **A** | G | **T** | **A** | **C** | **C** | **T** | **R** | **R** | **T** | **A** | A | T | A |
|  | *Rhagoletis completa / R. ramosae / R. zoqui* | 1 | 33 | A | A | **T** | **A** | C | **T** | **T** | G | A | **T** | **T** | **T** | **T** | **T** | T | **G** | **T** | **T** | **C** | **T** | T | **C** | **T** | **A** | **T** | **C** | **Y** | A | T | **A** | **Y** | **T** | **A** | A | **A** | **C** | A | G | **A** | G | **T** | **A** | **T** | **C** | **T** | **A** | **A** | **T** | **A** | A | T | A |
|  | *Rhagoletis fausta* | 5 | 1 | A | A | **T** | **A** | C | **T** | **T** | G | A | **C** | **T** | **T** | **T** | **T** | T | **A** | **T** | **T** | **A** | **T** | T | **C** | **T** | **A** | **A** | **T** | **T** | A | T | **A** | **T** | **T** | **A** | A | **A** | **C** | A | G | **T** | G | **T** | **A** | **T** | **T** | **A** | **A** | **A** | **T** | **A** | A | T | A |
|  | *Rhagoletis juniperina* | 4 | 8 | A | A | **T** | **A** | C | **T** | **T** | G | A | **T** | **T** | **T** | **T** | **T** | T | **G** | **T** | **T** | **A** | **T** | T | **C** | **T** | **A** | **G** | **T** | **T** | A | T | **A** | **T** | **T** | **A** | A | **A** | **C** | A | G | **C** | G | **T** | **A** | **T** | **T** | **A** | **A** | **A** | **T** | **A** | A | T | A |
|  | *Rhagoletis pomonella* | 7 | 4 | A | A | **T** | **A** | C | **T** | **T** | G | A | **T** | **A** | **T** | **C** | **T** | T | **A** | **T** | **T** | **R** | **C** | T | **C** | **T** | **A** | **T** | **T** | **T** | A | T | **A** | **T** | **T** | **C** | A | **T** | **C** | A | G | **C** | G | **T** | **A** | **A** | **T** | **A** | **A** | **A** | **T** | **A** | A | T | A |
|  | *Rhagoletis pomonella / R. mendax* | 8 | 9 | A | A | **T** | **A** | C | **T** | **T** | G | A | **T** | **A** | **T** | **C** | **T** | T | **A** | **T** | **T** | **R** | **C** | T | **C** | **T** | **A** | **T** | **T** | **T** | A | T | **G** | **T** | **C** | **A** | A | **A** | **T** | A | G | **C** | G | **T** | **A** | **T** | **T** | **G** | **G** | **A** | **T** | **A** | A | T | A |
|  | *Rhagoletis solanophaga* | 9 | 3 | A | A | **A** | **A** | C | **T** | **A** | G | A | **T** | **T** | **T** | **T** | **A** | T | **T** | **A** | **C** | **A** | **T** | T | **C** | **C** | **A** | **T** | **C** | **T** | A | T | **T** | **A** | **A** | **T** | A | **A** | **C** | A | G | **A** | G | **C** | **A** | **T** | **C** | **T** | **T** | **A** | **T** | **A** | A | T | A |
|  | *Rhagoletis striatella* | 10 | 2 | A | A | **C** | **A** | C | **A** | **A** | G | A | **T** | **T** | **T** | **T** | **A** | T | **T** | **A** | **T** | **A** | **A** | T | **C** | **T** | **A** | **T** | **T** | **T** | A | T | **A** | **A** | **C** | **A** | A | **A** | **C** | A | G | **A** | G | **T** | **A** | **T** | **C** | **T** | **T** | **A** | **C** | **G** | A | T | A |
|  | *Rhagoletis suavis* | 2 | 5 | A | A | **T** | **A** | C | **T** | **C** | G | A | **T** | **T** | **T** | **T** | **T** | T | **A** | **T** | **T** | **T** | **T** | T | **Y** | **W** | **W** | **T** | **C** | **T** | A | T | **A** | **T** | **T** | **A** | A | **C** | **C** | A | G | **A** | G | **T** | **A** | **C** | **C** | **T** | **A** | **A** | **T** | **A** | A | T | A |
|  | *Rhagoletis tabellaria* | 6 | 8 | A | A | **T** | **A** | C | **T** | **T** | G | A | **T** | **T** | **T** | **T** | **T** | T | **G** | **T** | **T** | **A** | **T** | T | **C** | **T** | **A** | **T** | **C** | **T** | A | T | **A** | **T** | **T** | **A** | A | **A** | **C** | A | G | **C** | G | **T** | **A** | **T** | **T** | **A** | **T** | **A** | **T** | **A** | A | T | A |

f)

| Group | OTU | OTU Nr | N | 103 | **108** | **111** | **129** | **131** | **132** | **141** | **142** | **151** | **156** | **174** | **180** | **198** | **222** | 242 | **243** | **246** | **253** | **261** | **312** | **315** | **319** | **321** | **324** | **336** | **354** | **423** | 433 | **448** | **453** | **465** | **469** | **477** | **490** | **492** | **515** | **525** | **533** | **540** | **547** | **562** | **570** | **571** | **577** | **579** | **582** | **585** | **598** | 600 | **606** | **621** | 633 |
| --- | --- | --- | --- | --- | --- | --- | --- | --- | --- | --- | --- | --- | --- | --- | --- | --- | --- | --- | --- | --- | --- | --- | --- | --- | --- | --- | --- | --- | --- | --- | --- | --- | --- | --- | --- | --- | --- | --- | --- | --- | --- | --- | --- | --- | --- | --- | --- | --- | --- | --- | --- | --- | --- | --- | --- |
| Others | *Acanthiophilus helianthi* | 99 | 1 | A | **A** | **T** | **A** | **C** | **T** | **A** | **A** | **A** | **T** | **T** | **A** | **T** | **A** | T | **T** | **T** | **T** | **T** | **T** | **T** | **C** | **T** | **A** | **C** | **T** | **T** | A | **T** | **A** | **A** | **T** | **A** | **A** | **A** | **C** | **T** | **G** | **T** | **G** | **T** | **C** | **A** | **T** | **A** | **A** | **A** | **T** | A | **T** | **A** | A |
|  | *Acanthonevra vaga* | 81 | 1 | A | **A** | **T** | **A** | **C** | **C** | **T** | **G** | **A** | **T** | **C** | **A** | **T** | **A** | T | **T** | **A** | **T** | **C** | **C** | **T** | **C** | **T** | **A** | **A** | **T** | **T** | A | **T** | **T** | **A** | **T** | **T** | **A** | **A** | **C** | **A** | **G** | **T** | **G** | **C** | **T** | **T** | **C** | **C** | **A** | **A** | **C** | A | **A** | **A** | A |
|  | *Acroceratitis nigrifacies* | 68 | 1 | A | **A** | **T** | **A** | **C** | **C** | **T** | **G** | **A** | **T** | **T** | **T** | **T** | **T** | T | **T** | **A** | **C** | **C** | **C** | **T** | **C** | **T** | **T** | **A** | **T** | **T** | A | **T** | **T** | **T** | **T** | **A** | **A** | **T** | **C** | **T** | **G** | **A** | **G** | **T** | **T** | **C** | **T** | **A** | **A** | **A** | **T** | A | **A** | **A** | A |
|  | *Bistrispinaria magniceps* | 116 | 1 | A | **A** | **T** | **A** | **C** | **T** | **A** | **G** | **A** | **T** | **C** | **T** | **T** | **T** | T | **C** | **C** | **C** | **C** | **C** | **C** | **C** | **A** | **T** | **C** | **T** | **T** | A | **T** | **A** | **A** | **T** | **A** | **A** | **C** | **C** | **T** | **G** | **T** | **G** | **T** | **T** | **C** | **T** | **A** | **A** | **C** | **T** | A | **A** | **G** | A |
|  | *Campiglossa albiceps* | 17 | 3 | A | **A** | **T** | **A** | **C** | **T** | **T** | **A** | **A** | **T** | **T** | **A** | **T** | **T** | T | **C** | **T** | **T** | **T** | **C** | **T** | **C** | **T** | **T** | **C** | **T** | **A** | A | **T** | **A** | **A** | **T** | **A** | **A** | **A** | **C** | **A** | **G** | **T** | **G** | **T** | **T** | **C** | **C** | **T** | **A** | **A** | **T** | A | **A** | **A** | A |
|  | *Campiglossa farinata* | 18 | 1 | A | **A** | **A** | **A** | **C** | **T** | **A** | **A** | **A** | **T** | **T** | **A** | **T** | **A** | T | **T** | **T** | **C** | **C** | **C** | **T** | **C** | **T** | **T** | **C** | **C** | **A** | A | **T** | **A** | **A** | **T** | **A** | **A** | **A** | **C** | **A** | **G** | **T** | **G** | **T** | **T** | **C** | **C** | **T** | **A** | **A** | **T** | A | **A** | **A** | A |
|  | *Campiglossa sabroskyi* | 19 | 1 | A | **A** | **T** | **A** | **C** | **T** | **T** | **A** | **A** | **T** | **T** | **A** | **T** | **T** | T | **T** | **T** | **T** | **C** | **C** | **T** | **C** | **T** | **T** | **C** | **T** | **A** | A | **T** | **A** | **A** | **T** | **A** | **A** | **A** | **C** | **A** | **G** | **T** | **G** | **T** | **T** | **C** | **C** | **T** | **A** | **A** | **T** | A | **T** | **A** | A |
|  | *Capitites ramulosa* | 96 | 1 | A | **A** | **A** | **A** | **C** | **T** | **A** | **A** | **A** | **T** | **T** | **A** | **T** | **T** | T | **T** | **T** | **T** | **T** | **C** | **T** | **C** | **T** | **A** | **A** | **C** | **T** | A | **T** | **T** | **A** | **T** | **A** | **A** | **T** | **C** | **T** | **G** | **T** | **G** | **T** | **T** | **A** | **C** | **T** | **A** | **A** | **T** | A | **G** | **A** | A |
|  | *Capparimyia aenigma* | 84 | 1 | A | **A** | **T** | **A** | **C** | **T** | **A** | **G** | **A** | **C** | **T** | **T** | **T** | **T** | T | **T** | **C** | **T** | **C** | **C** | **T** | **T** | **A** | **C** | **G** | **C** | **T** | A | **T** | **T** | **A** | **T** | **T** | **A** | **A** | **C** | **A** | **G** | **T** | **G** | **T** | **T** | **C** | **C** | **A** | **T** | **G** | **T** | A | **A** | **A** | A |
|  | *Capparimyia bipustulata* | 22 | 1 | A | **A** | **T** | **T** | **C** | **T** | **T** | **G** | **A** | **T** | **C** | **T** | **T** | **A** | T | **T** | **T** | **T** | **T** | **T** | **T** | **C** | **T** | **A** | **G** | **T** | **T** | A | **T** | **T** | **A** | **T** | **T** | **A** | **T** | **C** | **A** | **G** | **A** | **G** | **T** | **A** | **T** | **T** | **A** | **T** | **A** | **T** | A | **A** | **A** | A |
|  | *Capparimyia melanaspis* | 85 | 2 | A | **A** | **T** | **A** | **C** | **T** | **A** | **G** | **A** | **T** | **T** | **T** | **T** | **T** | T | **T** | **T** | **T** | **A** | **C** | **T** | **T** | **A** | **C** | **G** | **C** | **T** | A | **T** | **Y** | **T** | **T** | **T** | **A** | **T** | **C** | **A** | **G** | **T** | **G** | **T** | **T** | **C** | **T** | **A** | **T** | **A** | **T** | A | **A** | **A** | A |
|  | *Capparimyia savastani* | 23 | 1 | A | **A** | **T** | **T** | **C** | **T** | **T** | **G** | **A** | **T** | **T** | **T** | **T** | **A** | T | **T** | **T** | **T** | **C** | **T** | **T** | **C** | **T** | **A** | **A** | **C** | **T** | A | **T** | **T** | **A** | **T** | **A** | **A** | **T** | **C** | **A** | **G** | **A** | **G** | **T** | **G** | **C** | **T** | **A** | **A** | **A** | **T** | A | **A** | **A** | A |
|  | *Carpophthoromyia dimidiata* | 77 | 1 | A | **A** | **C** | **G** | **C** | **C** | **A** | **G** | **A** | **T** | **C** | **T** | **T** | **A** | T | **C** | **C** | **C** | **C** | **T** | **T** | **C** | **T** | **A** | **A** | **T** | **T** | A | **T** | **C** | **A** | **C** | **T** | **A** | **A** | **C** | **A** | **G** | **A** | **G** | **T** | **T** | **C** | **C** | **T** | **A** | **A** | **C** | A | **A** | **A** | A |
|  | *Carpophthoromyia pseudotritea* | 80 | 1 | A | **A** | **A** | **T** | **C** | **C** | **A** | **G** | **A** | **T** | **T** | **C** | **T** | **A** | T | **T** | **T** | **T** | **T** | **A** | **T** | **C** | **T** | **A** | **C** | **T** | **T** | A | **T** | **T** | **A** | **C** | **T** | **A** | **A** | **C** | **A** | **G** | **T** | **G** | **T** | **T** | **C** | **C** | **A** | **A** | **A** | **T** | A | **A** | **A** | A |
|  | *Carpophthoromyia vittata* | 78 | 1 | A | **A** | **C** | **A** | **C** | **C** | **A** | **G** | **A** | **T** | **C** | **T** | **T** | **A** | T | **A** | **C** | **C** | **A** | **T** | **T** | **C** | **T** | **A** | **A** | **T** | **T** | A | **T** | **C** | **A** | **C** | **A** | **A** | **A** | **C** | **A** | **G** | **A** | **G** | **T** | **C** | **C** | **C** | **T** | **A** | **A** | **C** | A | **G** | **A** | A |
|  | *Celidodacus obnubilus* | 71 | 1 | A | **A** | **T** | **A** | **C** | **C** | **T** | **G** | **A** | **C** | **T** | **T** | **T** | **A** | T | **T** | **C** | **T** | **T** | **T** | **T** | **C** | **T** | **A** | **G** | **T** | **T** | A | **C** | **T** | **A** | **T** | **A** | **A** | **A** | **C** | **A** | **G** | **T** | **G** | **T** | **T** | **C** | **C** | **T** | **A** | **A** | **T** | A | **A** | **A** | A |
|  | *Clinotaenia superba* | 117 | 1 | A | **A** | **A** | **A** | **C** | **C** | **A** | **G** | **A** | **C** | **A** | **C** | **T** | **T** | T | **T** | **A** | **T** | **A** | **G** | **T** | **T** | **A** | **T** | **T** | **N** | **T** | A | **T** | **G** | **A** | **T** | **A** | **A** | **A** | **C** | **C** | **G** | **T** | **G** | **T** | **T** | **C** | **T** | **A** | **T** | **A** | **T** | A | **T** | **A** | A |
|  | *Cyrtostola limbata* | 69 | 1 | A | **A** | **T** | **A** | **C** | **T** | **G** | **G** | **A** | **T** | **T** | **T** | **C** | **T** | T | **T** | **T** | **C** | **C** | **A** | **T** | **C** | **T** | **T** | **C** | **T** | **T** | A | **T** | **A** | **A** | **T** | **C** | **A** | **T** | **C** | **T** | **G** | **A** | **G** | **C** | **T** | **C** | **T** | **A** | **A** | **T** | **T** | A | **A** | **A** | A |
|  | *Dectodesis augur* | 97 | 1 | A | **A** | **T** | **A** | **C** | **T** | **A** | **A** | **A** | **T** | **T** | **A** | **T** | **T** | T | **T** | **T** | **T** | **T** | **A** | **T** | **C** | **T** | **A** | **G** | **C** | **T** | A | **T** | **T** | **A** | **T** | **A** | **A** | **T** | **C** | **T** | **G** | **T** | **G** | **T** | **T** | **A** | **C** | **T** | **A** | **A** | **T** | A | **A** | **A** | A |
|  | *Dioxyna picciola* | 20 | 5 | A | **A** | **T** | **A** | **C** | **T** | **A** | **A** | **A** | **T** | **T** | **A** | **T** | **T** | T | **T** | **T** | **T** | **T** | **C** | **T** | **C** | **T** | **T** | **C** | **T** | **A** | A | **T** | **A** | **A** | **T** | **A** | **A** | **A** | **C** | **T** | **G** | **T** | **G** | **T** | **T** | **C** | **C** | **T** | **A** | **A** | **T** | A | **A** | **A** | A |
|  | *Euaresta aequalis* | 103 | 3 | A | **A** | **T** | **T** | **C** | **T** | **N** | **A** | **A** | **T** | **T** | **A** | **T** | **T** | T | **C** | **Y** | **Y** | **C** | **T** | **T** | **C** | **T** | **A** | **T** | **T** | **A** | A | **T** | **T** | **A** | **T** | **A** | **A** | **T** | **C** | **T** | **G** | **C** | **G** | **T** | **T** | **T** | **Y** | **T** | **A** | **A** | **T** | A | **A** | **A** | A |
|  | *Euaresta bella / E. aequalis / E. festiva / Euphranta canadensis* | 102 | 11 | A | **A** | **Y** | **T** | **C** | **A** | **W** | **A** | **A** | **T** | **Y** | **A** | **T** | **T** | T | **C** | **T** | **T** | **C** | **T** | **T** | **C** | **T** | **A** | **C** | **T** | **A** | A | **T** | **T** | **W** | **T** | **A** | **A** | **Y** | **C** | **T** | **G** | **T** | **G** | **T** | **T** | **T** | **C** | **T** | **A** | **A** | **T** | A | **A** | **A** | A |
|  | *Euarestella iphionae* | 98 | 1 | A | **A** | **T** | **A** | **C** | **T** | **A** | **A** | **A** | **T** | **C** | **A** | **T** | **T** | T | **T** | **A** | **T** | **T** | **T** | **T** | **T** | **A** | **A** | **A** | **C** | **T** | A | **C** | **A** | **A** | **T** | **T** | **A** | **T** | **C** | **T** | **G** | **T** | **G** | **T** | **T** | **A** | **T** | **A** | **A** | **A** | **T** | A | **A** | **A** | A |
|  | *Euleia fratria* | 73 | 2 | A | **A** | **T** | **A** | **C** | **T** | **A** | **G** | **A** | **T** | **T** | **T** | **T** | **G** | T | **T** | **T** | **T** | **C** | **C** | **T** | **T** | **A** | **A** | **T** | **C** | **T** | A | **T** | **T** | **A** | **T** | **T** | **A** | **T** | **C** | **A** | **G** | **T** | **G** | **T** | **A** | **T** | **T** | **A** | **A** | **A** | **T** | A | **A** | **A** | A |
|  | *Euphranta canadensis* | 21 | 1 | A | **A** | **C** | **A** | **C** | **C** | **A** | **G** | **A** | **T** | **C** | **T** | **C** | **A** | T | **A** | **T** | **T** | **T** | **T** | **T** | **C** | **T** | **A** | **A** | **C** | **A** | A | **T** | **A** | **T** | **T** | **A** | **A** | **A** | **C** | **G** | **G** | **T** | **G** | **T** | **T** | **C** | **C** | **C** | **T** | **A** | **T** | A | **A** | **T** | A |
|  | *Eurosta comma* | 104 | 3 | A | **A** | **T** | **A** | **A** | **T** | **A** | **A** | **A** | **T** | **T** | **T** | **T** | **T** | T | **A** | **T** | **T** | **C** | **T** | **A** | **C** | **T** | **A** | **T** | **T** | **T** | A | **T** | **G** | **T** | **T** | **A** | **A** | **T** | **C** | **A** | **A** | **T** | **G** | **T** | **T** | **A** | **C** | **T** | **T** | **G** | **C** | A | **A** | **A** | A |
|  | *Eurosta solidaginis* | 105 | 13 | A | **A** | **T** | **T** | **A** | **C** | **D** | **A** | **A** | **T** | **T** | **T** | **T** | **A** | T | **A** | **C** | **C** | **T** | **C** | **A** | **C** | **T** | **A** | **C** | **T** | **T** | A | **T** | **C** | **T** | **T** | **A** | **A** | **T** | **C** | **A** | **G** | **T** | **G** | **T** | **T** | **C** | **C** | **T** | **A** | **A** | **C** | A | **A** | **A** | A |
|  | *Eutreta novaeboracensis* | 101 | 2 | A | **A** | **T** | **A** | **C** | **T** | **T** | **A** | **A** | **T** | **C** | **A** | **T** | **A** | T | **T** | **T** | **T** | **T** | **T** | **T** | **A** | **T** | **A** | **C** | **T** | **A** | A | **T** | **A** | **T** | **T** | **A** | **A** | **T** | **C** | **T** | **G** | **T** | **A** | **C** | **T** | **A** | **C** | **T** | **A** | **A** | **T** | A | **A** | **A** | A |
|  | *Felderimyia fuscipennis* | 139 | 2 | A | **A** | **T** | **A** | **C** | **R** | **T** | **G** | **A** | **T** | **C** | **T** | **C** | **A** | T | **A** | **T** | **T** | **Y** | **Y** | **A** | **T** | **A** | **A** | **A** | **T** | **T** | A | **C** | **R** | **T** | **T** | **A** | **A** | **T** | **C** | **A** | **G** | **T** | **G** | **T** | **T** | **C** | **C** | **T** | **T** | **T** | **T** | A | **T** | **A** | A |
|  | *Icterica seriata / I. circinata* | 15 | 3 | A | **A** | **T** | **T** | **C** | **T** | **A** | **A** | **A** | **T** | **C** | **A** | **T** | **T** | T | **T** | **A** | **T** | **Y** | **T** | **T** | **C** | **T** | **A** | **T** | **C** | **A** | A | **T** | **A** | **T** | **T** | **T** | **A** | **T** | **C** | **A** | **G** | **T** | **G** | **T** | **T** | **T** | **C** | **T** | **A** | **A** | **T** | A | **A** | **A** | A |
|  | *Neaspilota albidipennis* | 14 | 4 | A | **A** | **T** | **A** | **C** | **T** | **A** | **A** | **A** | **T** | **T** | **A** | **T** | **A** | T | **A** | **C** | **C** | **C** | **C** | **T** | **C** | **T** | **A** | **Y** | **T** | **A** | A | **T** | **A** | **T** | **T** | **A** | **A** | **T** | **C** | **T** | **G** | **A** | **G** | **T** | **T** | **C** | **C** | **T** | **A** | **A** | **T** | A | **A** | **A** | A |
|  | *Neoceratitis cyanescens* | 47 | 1 | A | **A** | **C** | **T** | **C** | **A** | **A** | **G** | **A** | **T** | **A** | **T** | **T** | **A** | T | **T** | **T** | **C** | **A** | **A** | **T** | **C** | **T** | **A** | **A** | **T** | **T** | A | **T** | **G** | **A** | **C** | **A** | **A** | **T** | **C** | **T** | **G** | **T** | **G** | **C** | **T** | **T** | **C** | **C** | **A** | **A** | **T** | A | **A** | **A** | A |
|  | *Perilampsis curta / P. mirathrix* | 25 | 2 | A | **A** | **T** | **T** | **C** | **A** | **A** | **G** | **A** | **T** | **T** | **T** | **T** | **A** | T | **T** | **T** | **T** | **T** | **Y** | **T** | **T** | **A** | **A** | **A** | **T** | **T** | A | **C** | **T** | **A** | **C** | **A** | **A** | **T** | **C** | **A** | **G** | **T** | **G** | **C** | **C** | **C** | **T** | **A** | **A** | **A** | **T** | A | **A** | **A** | A |
|  | *Perilampsis diademata* | 26 | 1 | A | **A** | **T** | **T** | **C** | **A** | **A** | **G** | **A** | **T** | **T** | **C** | **T** | **A** | T | **C** | **T** | **C** | **T** | **T** | **T** | **T** | **A** | **A** | **A** | **T** | **T** | A | **T** | **T** | **A** | **T** | **A** | **A** | **C** | **C** | **A** | **G** | **A** | **G** | **C** | **C** | **T** | **T** | **A** | **A** | **A** | **C** | A | **A** | **A** | A |
|  | *Perilampsis woodi* | 27 | 1 | A | **A** | **T** | **T** | **C** | **A** | **A** | **G** | **A** | **T** | **T** | **T** | **T** | **A** | T | **T** | **C** | **T** | **C** | **T** | **C** | **T** | **A** | **G** | **A** | **C** | **T** | A | **T** | **C** | **A** | **T** | **A** | **A** | **T** | **C** | **A** | **G** | **T** | **G** | **C** | **C** | **T** | **T** | **A** | **A** | **G** | **T** | A | **A** | **A** | A |
|  | *Procecidochares atra* | 89 | 2 | A | **A** | **T** | **A** | **C** | **T** | **A** | **A** | **T** | **T** | **T** | **T** | **T** | **A** | T | **A** | **A** | **A** | **T** | **T** | **T** | **A** | **T** | **A** | **T** | **T** | **A** | A | **T** | **A** | **T** | **T** | **A** | **G** | **A** | **C** | **T** | **G** | **T** | **G** | **T** | **T** | **T** | **C** | **T** | **A** | **G** | **T** | A | **T** | **A** | A |
|  | *Procecidochares atra* | 90 | 1 | A | **A** | **T** | **A** | **C** | **T** | **A** | **A** | **T** | **T** | **C** | **T** | **T** | **A** | T | **T** | **A** | **C** | **A** | **T** | **T** | **A** | **T** | **A** | **T** | **T** | **A** | A | **T** | **A** | **T** | **T** | **A** | **A** | **A** | **C** | **T** | **G** | **T** | **G** | **T** | **T** | **T** | **C** | **T** | **A** | **A** | **T** | A | **T** | **A** | A |
|  | *Rhagoletotrypeta rohweri* | 11 | 1 | A | **A** | **T** | **A** | **C** | **T** | **T** | **G** | **A** | **T** | **T** | **T** | **T** | **A** | T | **T** | **A** | **C** | **A** | **T** | **C** | **C** | **T** | **A** | **C** | **T** | **T** | A | **T** | **A** | **T** | **T** | **A** | **A** | **A** | **C** | **A** | **G** | **A** | **G** | **T** | **A** | **T** | **T** | **A** | **A** | **A** | **T** | A | **A** | **T** | A |
|  | *Strauzia longipennis* | 76 | 3 | A | **A** | **Y** | **A** | **C** | **T** | **A** | **G** | **A** | **T** | **A** | **T** | **T** | **A** | T | **T** | **A** | **C** | **C** | **T** | **T** | **T** | **A** | **M** | **T** | **C** | **A** | A | **C** | **A** | **R** | **T** | **C** | **A** | **T** | **C** | **A** | **G** | **T** | **G** | **T** | **A** | **T** | **T** | **A** | **A** | **A** | **T** | A | **A** | **A** | A |
|  | *Strauzia longipennis* | 74 | 1 | A | **A** | **T** | **A** | **C** | **T** | **A** | **G** | **A** | **T** | **A** | **T** | **T** | **A** | T | **T** | **A** | **C** | **C** | **C** | **T** | **T** | **A** | **A** | **T** | **C** | **A** | A | **C** | **A** | **A** | **T** | **C** | **A** | **T** | **C** | **A** | **G** | **T** | **G** | **T** | **A** | **T** | **T** | **A** | **T** | **T** | **C** | A | **A** | **A** | A |
|  | *Strauzia perfecta* | 75 | 1 | A | **A** | **A** | **G** | **C** | **T** | **A** | **G** | **A** | **T** | **A** | **A** | **T** | **A** | T | **T** | **A** | **C** | **C** | **C** | **T** | **T** | **A** | **A** | **T** | **C** | **A** | A | **C** | **A** | **A** | **T** | **C** | **A** | **C** | **C** | **A** | **G** | **T** | **G** | **T** | **A** | **T** | **T** | **A** | **A** | **A** | **C** | A | **A** | **A** | A |
|  | *Taeniostola vittigera* | 70 | 2 | A | **A** | **T** | **T** | **C** | **T** | **A** | **G** | **A** | **T** | **T** | **T** | **T** | **A** | T | **T** | **T** | **T** | **C** | **T** | **T** | **C** | **T** | **A** | **C** | **T** | **A** | A | **T** | **T** | **A** | **T** | **A** | **A** | **A** | **C** | **C** | **G** | **T** | **G** | **T** | **A** | **C** | **T** | **A** | **A** | **T** | **T** | A | **A** | **A** | A |
|  | *Tephritis araneosa* | 95 | 1 | A | **A** | **T** | **A** | **C** | **T** | **A** | **A** | **A** | **T** | **T** | **A** | **T** | **T** | T | **T** | **T** | **T** | **T** | **A** | **T** | **C** | **T** | **A** | **G** | **T** | **T** | A | **T** | **T** | **A** | **T** | **A** | **A** | **T** | **C** | **T** | **G** | **T** | **G** | **T** | **T** | **T** | **C** | **T** | **G** | **A** | **T** | A | **A** | **A** | A |
|  | *Tephritis pura* | 94 | 5 | A | **A** | **T** | **A** | **C** | **T** | **R** | **A** | **A** | **T** | **T** | **A** | **T** | **A** | T | **T** | **T** | **T** | **C** | **A** | **T** | **C** | **T** | **A** | **R** | **Y** | **T** | A | **T** | **T** | **A** | **T** | **A** | **A** | **T** | **Y** | **T** | **G** | **T** | **G** | **T** | **T** | **Y** | **C** | **C** | **R** | **A** | **T** | A | **A** | **A** | A |
|  | *Terellia palposa* | 87 | 1 | A | **A** | **T** | **A** | **C** | **T** | **A** | **A** | **A** | **T** | **T** | **T** | **T** | **A** | T | **T** | **T** | **T** | **T** | **A** | **T** | **C** | **T** | **G** | **C** | **T** | **T** | A | **T** | **A** | **A** | **T** | **A** | **A** | **T** | **C** | **T** | **G** | **T** | **G** | **C** | **A** | **T** | **C** | **A** | **T** | **A** | **T** | A | **A** | **A** | A |
|  | *Terellia ruficauda* | 88 | 1 | A | **A** | **A** | **T** | **C** | **T** | **A** | **A** | **A** | **T** | **A** | **T** | **T** | **A** | T | **T** | **T** | **T** | **T** | **A** | **T** | **C** | **T** | **G** | **C** | **T** | **A** | A | **T** | **A** | **A** | **T** | **A** | **A** | **T** | **C** | **T** | **G** | **T** | **G** | **C** | **A** | **T** | **T** | **A** | **T** | **A** | **T** | A | **A** | **A** | A |
|  | *Tomoplagia obliqua* | 91 | 2 | A | **A** | **T** | **A** | **C** | **T** | **T** | **A** | **A** | **T** | **A** | **T** | **T** | **A** | T | **C** | **T** | **T** | **C** | **T** | **T** | **C** | **T** | **A** | **C** | **T** | **T** | A | **T** | **G** | **A** | **T** | **T** | **A** | **T** | **C** | **T** | **G** | **T** | **G** | **T** | **T** | **A** | **C** | **T** | **A** | **A** | **T** | A | **T** | **A** | A |
|  | *Trirhithrum coffeae* | 53 | 2 | A | **A** | **T** | **T** | **C** | **A** | **G** | **G** | **A** | **T** | **T** | **T** | **T** | **T** | T | **A** | **T** | **C** | **A** | **T** | **T** | **C** | **T** | **G** | **A** | **T** | **A** | A | **T** | **A** | **A** | **T** | **A** | **A** | **T** | **C** | **A** | **G** | **T** | **G** | **T** | **A** | **T** | **T** | **A** | **A** | **C** | **T** | A | **T** | **A** | A |
|  | *Trirhithrum culcasiae* | 48 | 1 | A | **A** | **T** | **T** | **C** | **T** | **A** | **G** | **A** | **T** | **T** | **T** | **T** | **A** | T | **A** | **T** | **C** | **T** | **A** | **T** | **C** | **T** | **A** | **A** | **T** | **T** | A | **T** | **A** | **A** | **T** | **A** | **A** | **C** | **C** | **A** | **G** | **T** | **G** | **C** | **T** | **T** | **C** | **A** | **A** | **C** | **T** | A | **A** | **A** | A |
|  | *Trirhithrum demeyeri* | 79 | 1 | A | **A** | **C** | **A** | **C** | **T** | **A** | **G** | **A** | **T** | **T** | **T** | **T** | **T** | T | **C** | **T** | **T** | **C** | **G** | **T** | **C** | **T** | **A** | **A** | **C** | **T** | A | **T** | **C** | **T** | **T** | **T** | **A** | **T** | **C** | **A** | **G** | **T** | **G** | **T** | **C** | **C** | **C** | **T** | **T** | **A** | **T** | A | **A** | **A** | A |
|  | *Trirhithrum meladiscum* | 49 | 1 | A | **A** | **T** | **A** | **C** | **A** | **A** | **G** | **A** | **T** | **C** | **T** | **T** | **A** | T | **G** | **T** | **T** | **A** | **T** | **T** | **C** | **C** | **A** | **A** | **T** | **T** | A | **T** | **A** | **A** | **T** | **A** | **A** | **T** | **C** | **A** | **G** | **T** | **G** | **T** | **A** | **T** | **T** | **G** | **A** | **T** | **T** | A | **A** | **A** | A |
|  | *Trirhithrum nigerrimum* | 51 | 2 | A | **A** | **T** | **A** | **C** | **A** | **A** | **G** | **A** | **T** | **C** | **T** | **T** | **T** | T | **A** | **T** | **C** | **A** | **T** | **T** | **C** | **T** | **A** | **A** | **C** | **A** | A | **T** | **A** | **A** | **T** | **A** | **A** | **T** | **C** | **A** | **G** | **T** | **G** | **T** | **A** | **T** | **T** | **A** | **A** | **T** | **T** | A | **T** | **A** | A |
|  | *Trirhithrum quadrimaculatum* | 52 | 1 | A | **A** | **T** | **A** | **C** | **A** | **A** | **G** | **A** | **T** | **C** | **T** | **T** | **T** | T | **A** | **T** | **C** | **A** | **T** | **T** | **C** | **T** | **A** | **G** | **T** | **A** | A | **T** | **G** | **A** | **T** | **A** | **A** | **T** | **C** | **A** | **G** | **T** | **G** | **T** | **T** | **T** | **T** | **A** | **A** | **T** | **C** | A | **T** | **A** | A |
|  | *Trirhithrum senex* | 50 | 1 | A | **A** | **T** | **A** | **C** | **A** | **A** | **G** | **A** | **T** | **C** | **T** | **T** | **A** | T | **A** | **T** | **T** | **T** | **T** | **C** | **C** | **T** | **A** | **G** | **C** | **T** | A | **T** | **A** | **A** | **T** | **A** | **A** | **T** | **C** | **T** | **G** | **T** | **G** | **T** | **A** | **T** | **T** | **A** | **A** | **C** | **T** | A | **A** | **A** | A |
|  | *Trirhithrum teres* | 46 | 1 | A | **A** | **T** | **A** | **C** | **A** | **A** | **G** | **A** | **T** | **T** | **T** | **T** | **A** | T | **A** | **T** | **C** | **A** | **A** | **T** | **C** | **T** | **A** | **A** | **T** | **T** | A | **T** | **A** | **A** | **C** | **A** | **A** | **T** | **C** | **A** | **G** | **T** | **G** | **T** | **A** | **T** | **T** | **A** | **A** | **G** | **T** | A | **A** | **A** | A |
|  | *Trupanea actinobola* | 100 | 1 | A | **A** | **T** | **A** | **C** | **T** | **T** | **A** | **A** | **C** | **T** | **A** | **T** | **T** | T | **T** | **T** | **T** | **C** | **T** | **T** | **C** | **T** | **G** | **C** | **C** | **T** | A | **T** | **A** | **A** | **T** | **A** | **A** | **A** | **C** | **T** | **G** | **T** | **G** | **T** | **T** | **A** | **C** | **T** | **A** | **A** | **T** | A | **T** | **A** | A |
|  | *Trypeta flaveola* | 72 | 2 | A | **A** | **T** | **A** | **C** | **Y** | **R** | **G** | **A** | **T** | **T** | **T** | **T** | **A** | T | **A** | **T** | **T** | **A** | **A** | **A** | **T** | **A** | **C** | **A** | **T** | **T** | A | **T** | **T** | **A** | **T** | **A** | **A** | **A** | **C** | **A** | **G** | **W** | **G** | **T** | **T** | **T** | **T** | **A** | **A** | **A** | **Y** | A | **A** | **A** | A |
|  | *Urophora cardui / U. affinis* | 93 | 3 | A | **T** | **A** | **A** | **C** | **A** | **A** | **A** | **A** | **T** | **T** | **T** | **Y** | **A** | T | **T** | **T** | **T** | **A** | **T** | **T** | **C** | **T** | **A** | **R** | **T** | **T** | A | **T** | **A** | **Y** | **A** | **A** | **A** | **T** | **C** | **T** | **G** | **T** | **G** | **T** | **T** | **A** | **C** | **T** | **A** | **A** | **T** | A | **A** | **A** | A |
|  | *Urophora quadrifasciata / Langatia setinerva* | 92 | 5 | A | **T** | **A** | **A** | **C** | **A** | **A** | **A** | **A** | **Y** | **T** | **T** | **T** | **T** | T | **T** | **T** | **T** | **A** | **T** | **A** | **C** | **T** | **A** | **T** | **T** | **T** | A | **T** | **A** | **T** | **A** | **A** | **A** | **T** | **C** | **T** | **G** | **T** | **G** | **T** | **T** | **A** | **C** | **T** | **A** | **A** | **T** | A | **A** | **A** | A |
|  | *Xanthaciura tetraspina* | 86 | 1 | A | **A** | **A** | **A** | **C** | **T** | **T** | **G** | **A** | **T** | **A** | **T** | **T** | **A** | T | **T** | **A** | **C** | **T** | **T** | **T** | **T** | **A** | **A** | **A** | **T** | **T** | A | **T** | **T** | **A** | **T** | **A** | **A** | **G** | **C** | **A** | **A** | **A** | **G** | **T** | **T** | **T** | **C** | **T** | **T** | **A** | **T** | A | **A** | **A** | A |
|  | *Xanthomyia platyptera* | 16 | 2 | A | **T** | **A** | **A** | **C** | **A** | **T** | **A** | **A** | **T** | **T** | **A** | **T** | **A** | T | **T** | **C** | **C** | **A** | **A** | **C** | **C** | **T** | **A** | **C** | **N** | **A** | A | **T** | **T** | **A** | **T** | **A** | **A** | **A** | **C** | **T** | **G** | **C** | **G** | **T** | **T** | **C** | **C** | **T** | **A** | **A** | **T** | A | **A** | **A** | A |
